# Integrative Analyses of Hepatic Differentially Expressed Genes and Blood Biomarkers during the Peripartal Period between Dairy Cows Overfed or Restricted-Fed Energy Prepartum

**DOI:** 10.1371/journal.pone.0099757

**Published:** 2014-06-10

**Authors:** Khuram Shahzad, Massimo Bionaz, Erminio Trevisi, Giuseppe Bertoni, Sandra L. Rodriguez-Zas, Juan J. Loor

**Affiliations:** 1 Department of Animal Sciences and Division of Nutritional Sciences, University of Illinois at Urbana-Champaign, Urbana, Illinois, United States of America; 2 Illinois Informatics Institute, University of Illinois at Urbana-Champaign, Urbana, Illinois, United States of America; 3 Department of Animal and Rangeland Sciences, Oregon State University, Corvallis, Oregon, United States of America; 4 Istituto di Zootecnica and Centro di ricerca sulla nutrigenomica, Universitá Cattolica del Sacro Cuore, Piacenza, Italy; 5 The Institute for Genomic Biology, University of Illinois at Urbana-Champaign, Urbana, Illinois, United States of America; Technische Universität Dresden, Medical Faculty, Germany

## Abstract

Using published dairy cattle liver transcriptomics dataset along with novel blood biomarkers of liver function, metabolism, and inflammation we have attempted an integrative systems biology approach applying the classical functional enrichment analysis using DAVID, a newly-developed Dynamic Impact Approach (DIA), and an upstream gene network analysis using Ingenuity Pathway Analysis (IPA). Transcriptome data was generated from experiments evaluating the impact of prepartal plane of energy intake [overfed (OF) or restricted (RE)] on liver of dairy cows during the peripartal period. Blood biomarkers uncovered that RE vs. OF led to greater prepartal liver distress accompanied by a low-grade inflammation and larger proteolysis (i.e., higher haptoglobin, bilirubin, and creatinine). Post-partum the greater bilirubinaemia and lipid accumulation in OF vs. RE indicated a large degree of liver distress. The re-analysis of microarray data revealed that expression of >4,000 genes was affected by diet × time. The bioinformatics analysis indicated that RE vs. OF cows had a liver with a greater lipid and amino acid catabolic capacity both pre- and post-partum while OF vs. RE cows had a greater activation of pathways/functions related to triglyceride synthesis. Furthermore, RE vs. OF cows had a larger (or higher capacity to cope with) ER stress likely associated with greater protein synthesis/processing, and a higher activation of inflammatory-related functions. Liver in OF vs. RE cows had a larger cell proliferation and cell-to-cell communication likely as a response to the greater lipid accumulation. Analysis of upstream regulators indicated a pivotal role of several lipid-related transcription factors (e.g., PPARs, SREBPs, and NFE2L2) in priming the liver of RE cows to better face the early postpartal metabolic and inflammatory challenges. An all-encompassing dynamic model was proposed based on the findings.

## Introduction

The liver performs essential functions in mammals. These include, but are not limited to, gluconeogenesis and glycogen synthesis, synthesis of several plasma proteins encompassing clotting factors and acute phase proteins (APP) (e.g., haptoglobin, albumin, and fibrinogen), metabolism of amino acids and lipids, and detoxification including ammonia removal [1], [2]. During the period around parturition in dairy cattle, also known as the peripartal or “transition period” [2], the importance of the liver becomes even more critical due to the greater metabolic demands imposed by the onset of lactation, particularly the need to increase gluconeogenesis, fatty acid metabolism, and to control the inflammatory response [3], [4].

The level of dietary energy fed prepartum can alter the physiological adaptations to the transition period both in dairy [5] and beef cattle [6], [7]. Transcriptome profiling studies of peripartal cattle also demonstrated molecular adaptations in this organ some of which could alter its function, e.g. immune response and lipid metabolism [8]–[10]. The limited bioinformatics analyses performed in previous studies [9], [10] indicated that moderate overfeeding of energy pre-partum (OF) results in transcriptional changes predisposing cows to fatty liver and potentially compromising liver health early postpartum. The aggregation of the transcriptomics dataset from cows fed recommended [9] or different levels of energy prepartum [10] revealed an extremely large effect of OF or restricting energy (RE) prepartum on the transcriptome adaptations during the transition period with a very modest effect observed when cows were fed recommended levels of energy [11].

Due to the more pronounced effect on the liver transcriptome of prepartal OF or RE relative to feeding to requirements, in the present work we took advantage of the advancements in bioinformatics and statistical tools to re-analyze microarray data from the liver of OF and RE cows from the previously published study from Loor et al. [10]. The functional analysis of DEG between the two treatments at each time point during the transition period was performed using both the Dynamic Impact Approach (**DIA**), a novel bioinformatics approach developed by Bionaz et al. [12], and the classical enrichment analysis approach by means of Database for Annotation, Visualization and Integrated Discovery (**DAVID**) [13] coupled with previously published [10] and new blood biomarkers. In addition, we used Ingenuity Pathway Analysis (**IPA**) to study the upstream regulators of transcriptomics differences. The primary aim of the present study was to propose an all-encompassing dynamic model to explain the main effects of prepartal dietary management approaches on the physiological adaptations of transition dairy cattle.

## Results and Discussion

### Blood profiling and overall metabolism

#### Summary of previous data

We reported previously that prepartal high dietary energy (OF) vs. feed restriction (RE) markedly increased prepartal insulin concentration and early postpartum concentration of NEFA, BHBA, total protein, and liver TAG. The metabolism of palmitate in liver tissue slices also was more pronounced in OF vs. RE prepartum with no significant differences postpartum [10]. Those data clearly indicated that the higher energy prepartum had a strong metabolic effect during dietary treatment (i.e., pre-partum) and a carry-over effect during early post-partum, considering that in the postpartum the diet was the same for the two groups.

#### New blood biomarkers

**Figure 1:**
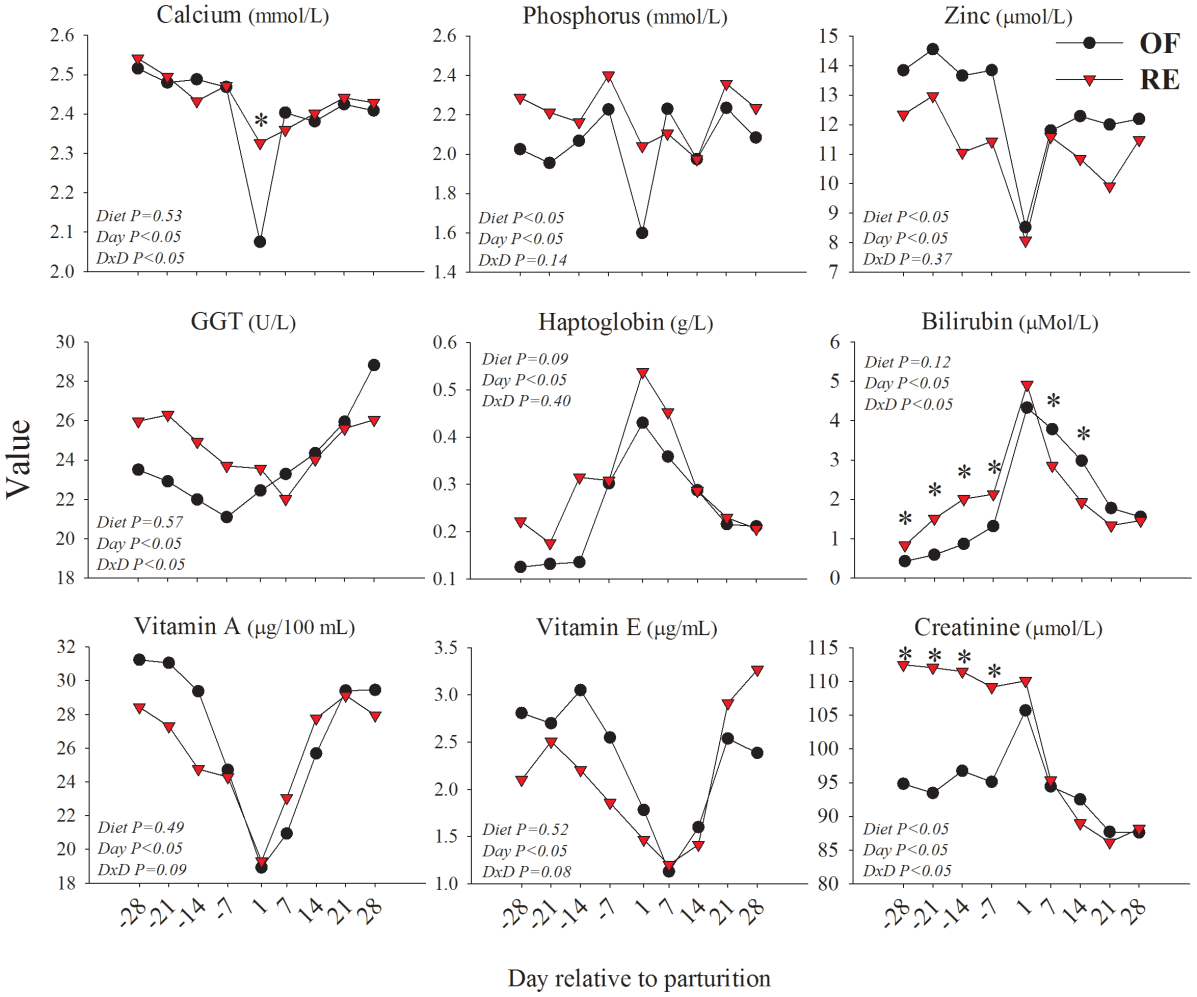
Results of plasma parameters significantly affected (*P*<0.05 or tendency *P*<0.10) by prepartal dietary energy level (OF  =  overfed energy and RE  =  restricted-fed energy prepartum) or diet × time interaction. *Difference between treatments at each time point. The GGT and Haptoglobin were log_2_-transformed while Bilirubin and Vitamin E were square root transformed before statistical analysis and back-transformed for plotting due to non-normal distribution.

To further understand the differential effects on metabolism and inflammation status between the two groups and to better interpret the transcriptomics differences in liver, we have performed analysis of an additional 17 blood biomarkers plus a re-analysis of total protein concentration (see Materials and Methods for details). Among the additional blood biomarkers reported in the present study those with an overall statistically significant (*p*<0.05) effect due to diet or diet × time or with tendency (*p*<0.10) are included in Figure 1. Results of other biomarkers that were unaffected by dietary treatment, are included in Figure S1. Among those was total protein, for which the statistical effect of the diet × time differed from the previous analysis [10]. This is likely due to the different assay used and the larger number of data points in the previous analysis [10] (Figure S1). However, the pattern of protein concentration over time was overall similar between the two analyses.

The results indicated that the concentration of phosphorus was overall lower and zinc overall greater in OF vs. RE. Calcium, gamma-glutamyl transpeptidase (GGT), bilirubin, and creatinine concentrations had a significant diet × time interaction while vitamin A and vitamin E concentrations tended to have a significant interaction. The concentration of haptoglobin tended to be overall greater in RE vs. OF.

As indicated by the numerically higher GGT and significantly higher bilirubin, the data suggest that during the prepartal period the liver from cows in RE experienced a more pronounced state of distress. This might be partly due to the inflammatory-like conditions in RE vs. OF, as indicated by the greater concentration of haptoglobin and lower concentration of zinc [14]. However, in our study the inflammatory-like conditions did not seem to be pronounced as indicated by the lack of differences in the plasma concentration of indices of negative APP [14] such as albumin, paraoxonase, and cholesterol [14], [15] (Figure S1). The moderately higher inflammatory-like conditions prepartum in RE vs. OF cows might have been a consequence of a dietary protein deficiency because the cows in this group were only allowed to consume feed to meet 80% of the overall dietary requirements including protein. This conclusion is partly supported by a previous study in rats, where an acute protein deficiency induced a low-grade inflammation [16]. In contrast, during the early postpartum period the blood biomarkers indicated that liver from cows in OF experienced a more pronounced state of distress, i.e., numerically greater GGT at 28 d and a larger increase soon after parturition, and larger bilirubin [14], [15] (Figure 1). The lower concentration of haptoglobin in OF vs. RE postpartum and the lack of difference in negative APP suggests that the observed stress response postpartum in OF vs. RE was not a consequence of higher inflammation [14], [15] but potentially a consequence of greater TAG accumulation in liver [10].

The numerical difference between the two groups in prepartum concentrations of vitamin A, and partly, vitamin E, are likely related with the dry matter intake which was higher in OF vs. RE prepartum (see [10] for feed intake). The concentration of vitamin E tended to be lower after +14 days relative to parturition (**d**) in OF vs. RE despite the lack of difference in feed intake postpartum [10] (Figure 1).

The markedly greater plasma creatinine in RE vs. OF prepartum was striking (Figure 1). Creatinine is considered an index of muscle mass and/or renal function (i.e., used for the estimation of glomerular filtration rate) [17], [18]. In our case the greater concentration of creatinine in RE cows prepartum was not due to greater muscle mass because it decreased in proportion to the body weight when cows were energy-restricted [5]. An increase of plasma creatinine due to feed restriction have been observed previously [19]–[21] but not always [22], [23]. A greater creatinine concentration in blood despite a lack of increase in muscle mass has been explained by a decrease in renal filtration and/or by an increase in muscle proteolysis [19]. In our study it is likely that RE cows had an increase in proteolysis due to a reduction in body weight [5]. This is supported by previous data from dairy cows where an increase of plasma creatinine was observed immediately before and after calving when proteolysis normally increases [24]. Creatinine also can be produced by the liver [18]. In our microarray we measured the expression of genes coding for 2 out of 3 enzymes involved in the formation of creatine phosphate from glycine and arginine, the guanidinoacetate N-methyltransferase (*GAMT*) and the creatine kinase, mitochondrial 1B (*CKMT1B*). The former was numerically and the latter was significantly more expressed in RE vs. OF cows (File S1). This might indicate an additional source of creatinine in RE vs. OF cows.

### Differentially expressed genes (DEG) between OF and RE

**Figure 2:**
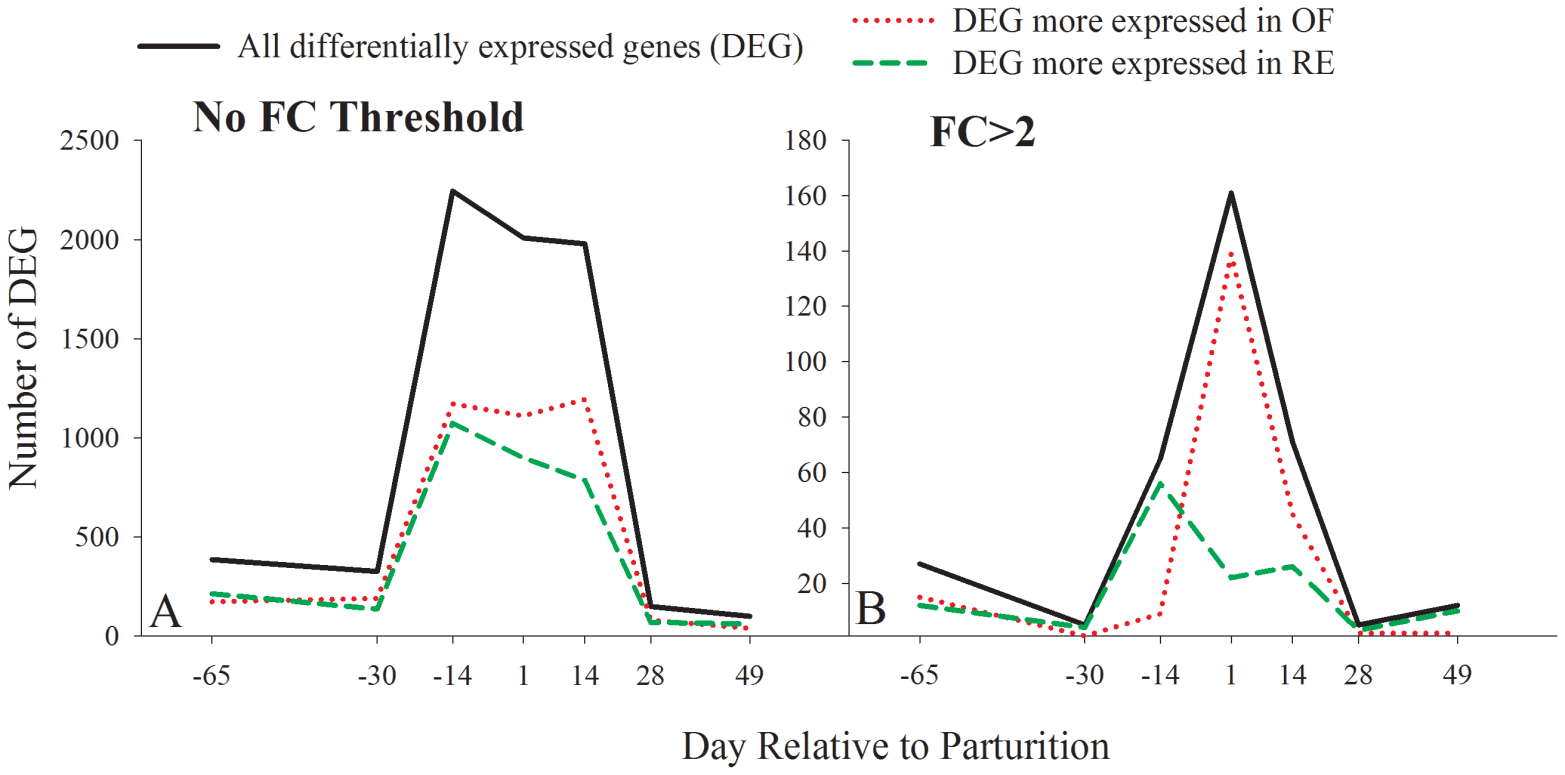
Number of differentially expressed genes (DEG) in liver of dairy cows fed restricted (RE) energy or receiving a higher energy diet (OF) prepaprtum. A). DEG with no fold change (FC) threshold. A larger number of DEG between the two diets were observed from two weeks prepartum to two weeks postpartum with a peak at −14 d. Except at 14 d, there was an overall similar number of genes with greater expression in OF vs. RE compared with those with greater expression in RE vs. OF. At 14 d there was an increase in the number of genes that were more expressed in OF vs. RE compared with RE vs. OF. B). DEG with 2-fold change (FC) cutoff. The total number of DEG between the two diets with >2-fold difference in expression was <7% of total DEG. Most of the differences in gene expression between OF *vs*. RE occurred during −14 d to 14 d. The larger number of DEG more expressed in RE vs. OF was observed at −14 d while the larger number of DEG more expressed in OF vs. RE was observed at +1 d.

A mixed model ANOVA with FDR correction resulted in 4,790 cDNA array ID with a time × diet interaction (FDR≤0.05). Out of these cDNA ID, 4,111 were annotated with bovine Entrez gene ID (3,460 unique Entrez gene ID). Among these only the genes with a significant difference (*p*≤0.05) between OF and RE at each time point were used for the analysis (Figure 2). The number of DEG in Figure 2 indicated that there was a marked difference in expression of genes due to dietary energy level prepartum. A large number of DEG was observed between −14 to +14 d. It is noteworthy that the number of DEG between OF and RE did not change (or even decreased) during the first 35 days of the dietary treatment but changed tremendously afterwards. The large number of DEG observed up to 2 weeks after the treatments ceased is indicative of a brief, but significant, carry-over effect, i.e. an effect lasting through a stage when the same diet was fed to both groups of cows [10].

From the above data, it is obvious that at +1 d and, more pronounced, at +14 d, there was a greater number of DEG more expressed in OF vs. RE compared with the DEG more expressed in RE vs. OF (Figure 2A), indicating a larger transcriptional sensitivity of the OF group compared with the RE group due to stimuli from parturition/initiation of lactation. By applying the >2-fold ratio expression threshold, the number of DEG was greater in RE vs. OF prepartum, but there were more DEG with a higher expression of >2-fold in OF vs. RE postpartum, particularly on day 1 post-partum (OF [n = 99] vs. RE [n = 21]) Figure 2B. The greater gene expression and fold difference at +1 d in OF vs. RE indicates an overall larger liver transcriptional activity due to higher dietary energy prepartum.

### Summary view of KEGG pathway analysis

**Figure 3:**
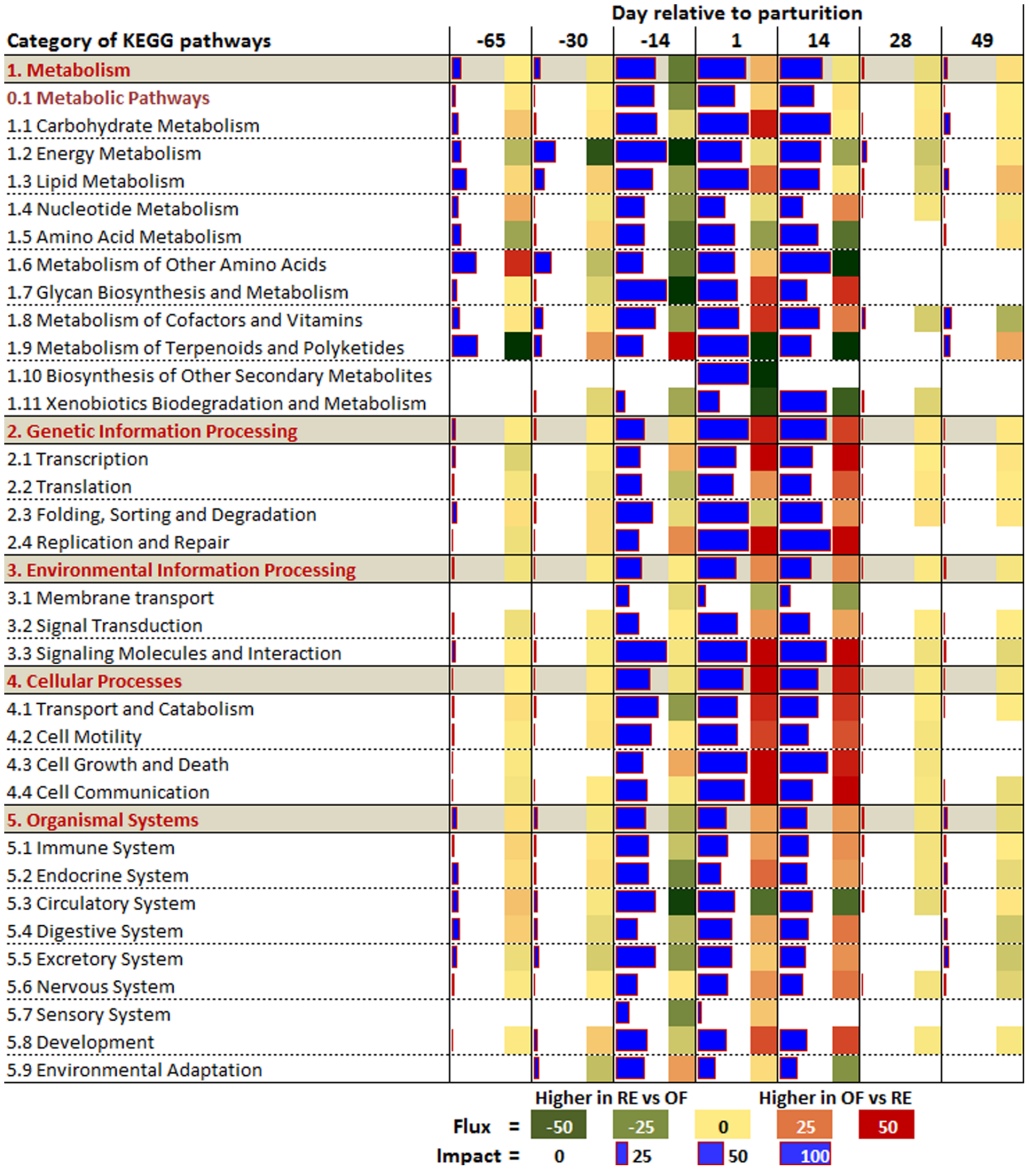
The summary of KEGG pathways encompassing categories and sub-categories of pathways as provided by the Dynamic Impact Approach (DIA). The ‘Impact’ is represented by the horizontal blue bars (larger the bar larger the impact) and the ‘Direction of the Impact’ (Flux) is represented by green (more induced in RE vs. OF) to red (more induced in OF vs. RE) rectangles.

The “Impact” value in the DIA analysis represents an estimate of the perturbation in a biological pathway, while the “Direction of the Impact” (or flux) value represents the overall direction of the perturbation (i.e., a pathway is either activated or inhibited) [12]. The DIA also provides a summary of the KEGG pathways in the form of categories and sub-categories (Figure 3), besides providing the details of each pathway (File S2: sheet “Details of pathways”).

In accordance with the number of DEG (Figure 2) all the KEGG pathway categories were more impacted from −14 d to +14 d in parallel with the other time comparisons. The category ‘Metabolism’ followed by the ‘Genetic Information Processing’ was the most-impacted (Figure 3). With exception of the subcategories of pathways within ‘Metabolism of Terpenoids and Polyketides’, ‘Transcription’, ‘Replication and Repair’, ‘Cell Growth and Dead’, and ‘Environmental Adaptation’, all other categories and sub-categories of pathways were more induced in RE vs. OF at −14 d.

Few of the subcategories of pathways were or remained more induced in RE vs. OF until 2 weeks postpartum (Figure 3). This was evident particularly for ‘Energy Metabolism’, ‘Amino Acid Metabolism’, ‘Metabolism of Other Amino Acids’, ‘Xenobiotics Biodegradation and Metabolism’, ‘Membrane Transport’ and ‘Circulatory System’. Almost all the other subcategories of pathways had a larger induction in OF vs. RE early postpartum which was more evident in non-metabolic related sub-categories of pathways with the exception of ‘Glycan Biosynthesis and Metabolism’ and ‘Metabolism of Cofactors and Vitamins’. A very large induction in OF vs. RE postpartum was observed for subcategories of pathways related to ‘Transcription’, ‘Translation’, cell proliferation (e.g., ‘Cell Growth and Death’ and ‘Replication and Repair’), signaling and cell-to-cell communication (e.g., ‘Signaling Molecules and Interaction’ and ‘Cell Communication’) (Figure 3).

The summary of the KEGG pathways indicated that after ca. 50 days of dietary treatment prepartum (coinciding with 2 weeks prepartum) the RE cows had an overall larger induction of the pathways, particularly metabolic-related. With the exception of few pathways, the postpartum carry-over effect was characterized by an overall large induction of non-metabolic related pathways in OF cows (Figure 3). In addition, the larger induction of transcription-related pathways in OF vs. RE is supportive of the greater number of DEG in OF vs. RE compared with RE vs. OF from +1 d to +14 d (Figure 2). Those results are suggestive of increased cell proliferation (i.e., larger induction of ‘Replication and Repair’ and ‘Cell Growth and Dead’ sub-categories of pathways) in OF vs. RE, as also discussed previously [11]. The ‘Human Disease’ KEGG pathways category was not included in the results and discussion section because of its low biological significance for the present study (however, the results can be found in File S2).

### Biological interpretation of KEGG pathways in combination with Gene Ontology and enrichment analyses

**Figure 4:**
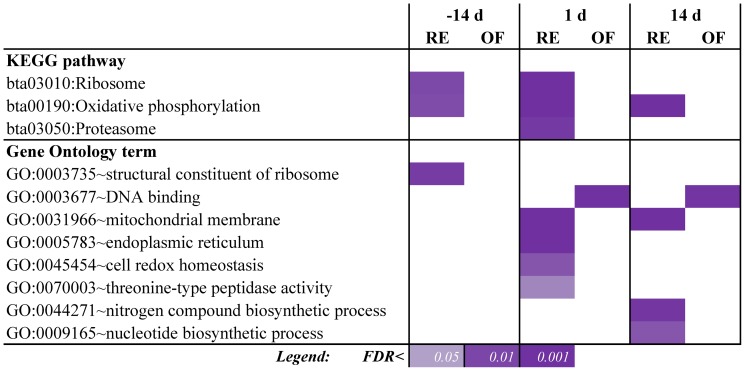
Summary of GO and KEGG results from the DAVID analysis for DEG in liver between cows fed restricted (RE) or receiving a higher energy diet (OF) prepaprtum. Reported are the unique significantly-enriched terms with a Benjamini FDR<0.05 for at the least one comparison at −14, 1, and/or 14 d. Purple shades denote enrichment with a FDR<0.05.

Here we provide an integral view of the pathways combining the DIA results (File S2) with the visual results of several pathways obtained via KegArray (File S3) plus DIA results of Gene Ontology (**GO**) biological process (Files S2 and S4) and enrichment analysis performed using DAVID (Figure 4 and File S5 for details). In order to simplify the interpretation of data, the discussion was separated between metabolic-related and non-metabolic related pathways. The pathways related to metabolism were discussed in combination with the blood profiling reported in Figure 1 and the data reported previously [10].

#### Dietary energy and protein restriction prepartum might prime the liver to face high metabolic demands

**Figure 5:**
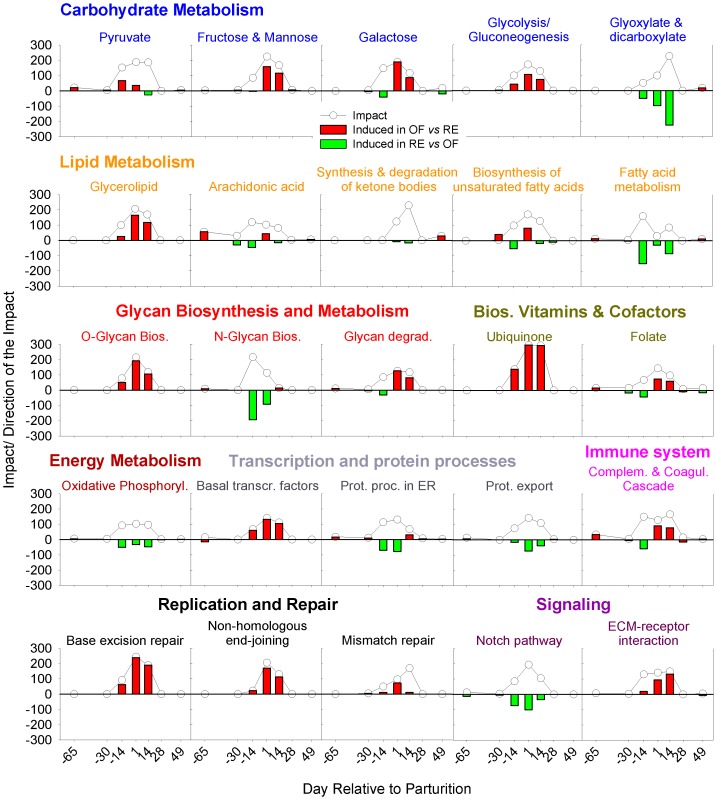
Dynamic Impact Approach (DIA) results (Impact and Direction of the Impact) for the 25 most impacted KEGG pathways grouped in sub-categories of pathways.

It has been clearly established that the metabolism of carbohydrate [25], energy, lipid [26]–[28], and nitrogen [29] is critical in the liver of transition dairy cows. Liver has a great flexibility to adapt to the metabolic changes occurring during the transition period [30]. Our data indicated that pathways related to the above metabolism categories were highly-impacted by the prepartal level of energy and protein intake in the diet (Figure 3). The details of the pathways (File S2) revealed a large effect of increased dietary energy and protein intake prepartum (i.e., high impact) on ‘Carbohydrate Metabolism’ (Figure 3). Considering the most-impacted pathways (Figure 5), the OF cows had a larger induction of several carbohydrate-related pathways between −14 d and +14 d, including ‘Glycolysis/Gluconeogenesis’. Considering only the ‘Glycolysis/Gluconeogenesis’ pathway it is difficult to ascertain if the glucose metabolism was more towards synthesis or utilization of glucose (File S3). In a previous experiment, liver from cows fed ad-libitum vs. restricted before parturition had only numerically higher glycogen concentration [5]. The larger plasma insulin prepartum in OF vs. RE [10] might have played a role in determining the expression of genes related to carbohydrate metabolism, and might have induce glycogenesis [31]. However, the higher glucose and insulin prepartum in OF vs. RE are indicative of a decrease in insulin sensitivity; in rats, insulin resistance has been associated with decreased liver glycogen content [32]. Therefore, the cause for the response in gene expression observed might be due to the contrasting effect of higher insulin but also insulin resistance.

The liver of RE cows had a higher activation both pre- and post-partum of energy production through the ‘Oxidative phosphorylation’ pathway (Figure 5). The data also indicated a higher induction of ‘Citrate cycle (TCA)’ and degradation of amino acids (AA; Files S2 and S3) in RE vs. OF during the peripartal period, which support the apparently greater proteolysis suggested by the higher creatinine (Figure 1). The AA can play an important role during early lactation to meet the glucose requirements through hepatic gluconeogenesis [33]. In addition certain AA such as alanine, aspartate and glutamate play a significant role in hepatic gluconeogenesis during starvation or nutrient restriction [34]. Overall, the AA metabolism was more induced in RE vs. OF (File S2, Figure 3). The data indicated a greater utilization of AA for gluconeogenesis in RE vs. OF, as is the case for ‘Alanine, aspartate and glutamate metabolism’ that was more induced at −14 d in RE vs. OF cows (File S3), likely due to greater glucose synthesis in this group as consequence of restricted energy and protein intake [35].

Among the energy-related pathways, as for the ‘Oxidative phosphorylation’, also the ‘Sulfur metabolism’ was more activated in RE vs. OF, particularly post-partum (File S2). The role of sulfur metabolism in the liver of periparturient dairy cows has not yet been investigated thoroughly. However, because of the anionic property of the sulfur compounds, this pathway appears essential in order to balance the cation-anion concentrations in the liver [36]. It may also be involved in the synthesis of sulfur containing AA [37]. The metabolism of sulfur in sulfur-containing compounds (e.g., enzymes, hormones and xenobiotics) can play an important role in the regulation of different cellular and metabolic processes in the liver such as the sulfate-conjugation of xenobiotics and steroid hormones which are needed for their metabolism, bioactivation and detoxification often resulting in a decrease in biological activity and an increase in their urinary excretion [38].

The pathways involved in lipid synthesis, especially ‘Glycerolipid metabolism’, were evidently more induced postpartum in OF vs. RE (Figure 5), which is consistent with a greater degree of esterification of fatty acids (FA) observed *in vitro* [10]. This mechanism also was supported by the GO BP analysis with DIA, which uncovered a higher activation in OF vs. RE of terms related to TAG synthesis and storage (File S4). The data also suggested that during the last month of being on diets (i.e. end of pregnancy) the RE vs. OF cows had a lower degree of sterol synthesis, which is consistent with the observed inhibition of cholesterol synthesis in cows feed-restricted both during mid-lactation [39] or in early postpartum [40]. However, in our experiment, the blood biomarker analyses did not reveal differences in blood cholesterol between the two groups. The data from DIA indicated that the RE cows compared with OF had an overall higher induction of sterol synthesis early postpartum (File S2) which also was supported by the DIA analysis of GO BP (File S4). At least in humans, cholesterol is essential for the synthesis of very-low density lipoproteins [41], and this is the only means for liver to export TAG to peripheral tissues. The above data indicate that the higher liver TAG observed in the OF vs. RE group might be due to a concomitant higher synthesis of TAG and lower VLDL formation (also due to lower cholesterol availability).

Despite having a greater NEFA concentration postpartum [10], the transcriptomics data suggest that cows in OF vs. RE had a lower degree of lipid catabolism (Figure 5). As previously proposed [11], a greater NEFA concentration rather than a change in gene expression appears more important in terms of a flux increment towards oxidation leading to ketone body production. This is supported by the lower overall induction of synthesis of ketone bodies in OF vs. RE during the first two weeks postpartum (Figure 5) despite the greater plasma BHBA [10]. However, the apparently greater fatty acid catabolism in RE vs. OF is not entirely supported by the *in vitro* oxidation of palmitate, which did not differ between groups [10]. Taken together, the modest induction of genes related to synthesis of cholesterol and lower fatty acid catabolism coupled with the higher induction of genes involved in synthesis of TAG in OF vs. RE might have contributed to the greater degree of liver TAG accumulation observed in OF vs. RE [10].

The observations highlighted above indicate that RE vs. OF induced a higher degree of degradation of molecules in the liver in order to obtain energy during the feed restriction period but, paradoxically, also when the cow was allowed to consume ad-libitum energy- and protein during the postpartal period. Together, the data indicate that the dietary treatment prepartum “primed” the liver of RE cows for higher metabolic capacity. In support of this in RE vs. OF cows there also was a greater induction of ‘Fatty acid metabolism’ and other energy-related pathways such as ‘Sulfur metabolism’ and ‘Oxidative phosphorylation’. The latter also was enriched in DAVID analysis together with enrichment of mitochondria and oxidative related terms (Figure 4 and File S5). Terms related to mitochondria and oxidation were also among the most-impacted GO BP terms in DIA, at least at −14 d (File S4).

#### Glycan biosynthesis and ER stress

Glycans are carbohydrate molecules that are linked with lipids and protein moieties to form glycolipids and glycoproteins and also act as signaling molecules [42]. The most-impacted glycan pathways in our experiment included ‘O-Glycan biosynthesis’, ‘Other glycan degradation’, and ‘N-Glycan Biosynthesis’ (Figure 5 and File S2). Except the latter, all were more induced in OF vs. RE (Figure 5). The potential role of N-Glycan biosynthesis in the liver is to handle the misfolded proteins in the endoplasmic reticulum (ER) during stress conditions [43]. During protein synthesis, the function of the ER is affected by both intracellular and extracellular stimuli leading to ER stress, which results in accumulation of misfolded proteins in the ER lumen [44]. It is interesting that the ‘Protein processing in the ER’ pathway was among the most impacted and had a similar induction as ‘N-Glycan Biosynthesis’ in RE vs. OF (Figure 5). This might be indicative of a greater degree of ER stress, likely related to the higher oxidative conditions in RE vs. OF cows during the last month of dietary treatments; however, our inference needs to be corroborated by quantitative measurements of ER stress prepartum. The data also could be indicative of an increased capacity of the liver prepartum in RE cows in order to handle ER stress postpartum. The ER stress response in RE cows appears to be partly under control of the transcription factor *XBP1* (X-box binding protein 1), as previously discussed [45].

#### Dietary energy and protein restriction prepartum might prime the liver for a better response to the inflammatory challenge of early lactation

Among the non-metabolic related pathways the most-impacted were related to transcription, translation, cell cycle and cell signaling/communication (Figure 5 and File S2). Overall, during the postpartum these categories of pathways were more induced in OF vs. RE (Figure 3). The details of these pathways (Files S2 and S3) clearly support a larger induction of transcription, replication and repair of DNA, and cell-to-cell interaction/communication in OF vs. RE postpartum; however, the ‘Ribosome’ pathway (i.e., protein synthesis machinery components) and pathways related to protein synthesis (e.g., ‘Protein export’ and ‘Proteasome’) were overall more induced in RE vs. OF (File S2). This also was supported by the significant enrichment of pathways and GO terms related to ribosome within the DEG related to greater expression in RE *vs.* OF at −14 d and +1 d (Figure 4). However, the “Proteasome” pathway was more induced in RE vs. OF at +14 d which was also supported by the enrichment analysis (Figure 4). These data indicate that, on the one hand, the overall amount of RNA (and/or number of different transcripts) was greater in OF vs. RE, as supported by the number of DEG (Figure 1), but, on the other hand, the overall degree of protein synthesis and export, particularly in the ER (File S2), was greater in RE vs. OF (Figure 5), as also discussed in the previous section.

The higher protein synthesis and protein export in liver of RE vs. OF cows may be associated with a greater capacity to produce and export proteins such as signaling molecules (e.g., IGFBPs and interleukins, see File S1) and positive APP. This is supported by the higher concentration of plasma haptoglobin in RE vs. OF cows (Figure 1) but also by the greater expression of some of the known positive APP measured by the microarray (Figure S2 and File S2). A greater production of positive APP is normally associated with a decrease of negative APP [14]. This phenomenon is associated with an impaired capacity of the liver to face metabolic challenges, as indicated by the detrimental effect of inflammation on the peroxisome proliferator-activated receptor (PPAR)[46], which (at least in non-ruminants) is involve in assuring the normal functions carried out by the liver [14], [15]. It is noteworthy that both the blood biomarker (Figure 1 and Figure S1) and gene expression analyses (Figure S2) indicated a more pronounced inflammatory-like conditions around parturition in RE vs. OF cows (Figure 1), which did not appear to elicit a detrimental effect on the “normal” liver function. There was no detectable lower plasma concentration of negative APP in RE vs. OF (Figure S1) or in their mRNA abundance in liver (Figure S2).

A higher capacity for or sensitivity to an inflammatory response in RE vs. OF also was suggested by the observed changes in the ‘Arachidonic acid metabolism’ pathway (Figure 5), particularly at the end of the dietary treatment phase (i.e. parturition). Arachidonic acid is a long-chain polyunsaturated fatty acid [47], and the larger induction of its metabolism may be indicative of an increased rate of inflammation via the production of pro-inflammatory lipids such as prostaglandins [48].

Overall, the immune-related pathways suggested a higher immune/inflammatory response in RE vs. OF by the end of pregnancy/dietary treatment phase but with a greater induction of the same pathways postpartum in OF vs. RE (Figure 3), especially for the ‘Complement and coagulation cascades’ (Figure 5). This pathway is part of the innate immune response and links the inflammatory response (i.e., complement) with coagulation [49]. The detailed visualization of the pathway (File S3) uncovered that the induction in OF vs. RE was exclusively due to the coagulation pathway.

The higher immune/inflammatory response in RE vs. OF also was suggested by the greater induction of ‘NOD-like receptor signaling pathway’ in RE vs. OF cows. This innate immune response pathway has been previously reported to be among the most-activated in several studies involving mammary gland bacterial infection [50]. Innate immune response-related pathways including ‘Toll-like receptor’ (File S2), but especially the pathways related to the immune cell migration or activity (e.g., ‘Leukocyte transendothelial migration’, and ‘T cell receptor signaling pathway’), were more induced in OF vs. RE, particularly at +14 d. The DIA analysis of GO BP indicated a larger activation of macrophage in OF vs. RE but, contrary to the KEGG pathway results, indicated a more induced chemotaxis of some of the immune cells in RE vs. OF at +14 d (File S4).

Overall, the data suggest that the liver in RE cows was likely more responsive to inflammatory-like conditions and also better able to handle them. Despite the apparently higher inflammatory response the liver did not have a decrease of negative APP or inhibition of metabolic or detoxification pathways. The greater detoxification capacity of liver of RE vs. OF cows was substantiated by the lower plasma bilirubin (e.g., higher clearance capacity) in early lactation (Figure 1) and inferred by the higher activation of ‘Drug metabolism – other enzymes’ and ‘ABC transporters’ (File S2). The ATP binding cassette (ABC) transporters play an important role in removal of xenobiotic compounds through their excretion in bile salts [51], [52].

The data suggest that the OF vs. RE had a higher activation of immune cells but there is some discordance about the capacity of the immune cells to perform chemotaxis. The suggested higher activation of the immune cells in the present analysis appears to be similar to what is observed in monogastrics during the occurrence of fatty liver [53]. It is noteworthy that our data suggest a positive role of moderate inflammatory-like conditions in the peripartal period to prime the liver for a better inflammatory and metabolic response post-partum. Those findings seem to support recent findings where inhibition of inflammatory-like conditions using salicylate in early postpartal cows had a negative effect on the homeorhetic adaptations of the liver [54].

It is important to stress the word “moderate” in the context of inflammation. Previous studies from some of the authors have clearly established that substantial and prolonged inflammatory-like conditions (i.e., haptoglobin >0.3 g/L and a significant decrease of negative APP for the first two weeks postpartum) negatively affect productive and reproductive performance and increase the likelihood of developing health disorders in dairy cows early postpartum [14], [15]. In addition, several studies have observed a positive effect of preventing/decreasing inflammation in peripartal cows [39], [56]. Overall, the present data support a positive role of moderate and likely diet-derived-stress-related response inflammatory-like conditions on the liver of transition dairy cows, especially post-partum.

#### Higher cell signaling and cell-to-cell communication in OF vs. RE: a response to liver fat accumulation?

The proliferation of hepatic cells appeared to be greater in OF vs. RE, as indicated by an overall higher induction of KEGG pathways and GO BP terms related to cell cycle (Figure 3 and Files S2 and S4), including the ‘DNA replication and repair’ pathway (Figure 5). Reduced proliferation due to an acute energy restriction has been observed previously [55]. It is noteworthy that the higher induction of pathways related to proliferation in liver of OF vs. RE was observed postpartum (i.e., carryover effect). This could have been due to the greater degree of lipid accumulation. Ethanol-induced steatosis in liver of rats increased hepatocyte proliferation as a mechanism to reduce the injury due to the large degree of lipid infiltration [56].

The potentially greater degree of liver proliferation also was accompanied by an overall greater degree of cell-to-cell communication in OF vs. RE, as suggested by a larger induction of signaling and cell communication-related pathways (Figure 3) such as ‘ECM [Extracellular matrix] receptor interaction’ (Figure 5) and GO terms related to ECM disassembly (File S4). The activation of the extracellular matrix is a common finding in non-ruminant liver steatosis [57] and it is determined by the interplay between several cell types but apparently initiated by stellate cells. In line with this previous observation, our data suggest that macrophages cells were more activated in OF vs. RE (e.g., more induced macrophage activation, see File S4; and several immune-related KEGG pathway, File S2).

The change in ECM deposition is an essential step for the onset of fibrosis, which is one marker of liver damage. It is challenging to conclude that the liver in OF cows was damaged compared with RE cows because a histological analysis was not performed; however, functional analysis of transcriptome differences between the two groups of cows, the numerically greater amount of GGT at 28 d, the higher increase in plasma bilirubin compared with pre-partum (although not pathological), and the greater hepatocellular TAG [10] could be considered reasonable indicators of a more injured liver in OF compared with RE.

With the exception of ‘Notch signaling’ and ‘Hedgehog signaling’ all the pathways involved in signaling communication were more induced in OF vs. RE (Figure 3) (Figure 5 and File S2). The inhibition of ‘Notch signaling’ in OF vs. RE is likely a consequence of the greater lipid accumulation. It has been clearly demonstrated that steatosis in mice inhibits Notch signaling [58]. It is noteworthy that the pathway was already more inhibited in OF vs. RE at −14 d, well-before the greater TAG concentration was observed (Figure 5 and [10]). This indicate that the larger inhibition of the Notch signaling pathway in OF vs. RE prior to the peak of NEFA might have allowed for a greater lipid accumulation postpartum. This idea is supported by the higher induction of ‘MAPK signaling’ in OF vs. RE particularly during early lactation (File S2). This pathway is mainly involved in the control of cellular growth and proliferation but it also is essential for the induction of liver steatosis in mouse by cross-talk with the peroxisome proliferator-activated receptor gamma (PPARγ; [59]). Although our data seem to support such a role, the expression of PPARγ in bovine liver is nearly non detectable relative to the alpha and delta isotypes [60].

The PPAR, particularly PPARα, is considered important in the whole economy of lipid metabolism in liver of mammals, including dairy cows during the transition period [60]. Our analysis indicated that the ‘PPAR signaling’ pathway (which includes all three PPAR subtypes) played a role in regulating aspects of lipid metabolism in response to different levels of dietary energy fed prepartum. As commonly observed in monogastrics [61], the ‘PPAR signaling’ pathway was more induced in liver of RE compared with OF cows (File S2). It is noteworthy that the larger activation of ‘PPAR signaling’ in RE vs. OF also was observed at +14 d, namely due to the higher expression of genes encoding for proteins involved in FA oxidation (File S3). These data support a role of PPAR in controlling lipid catabolism in response to prepartal dietary energy level. However, the higher activation of this pathway in RE vs. OF in spite of a lower concentration of plasma NEFA [10], which should increase its activation [60], also supports the idea of a liver in RE vs. OF that was better “primed” to respond to the increased NEFA flux postpartum potentially by having a higher metabolic capacity.

### Up-stream regulators

**Figure 6:**
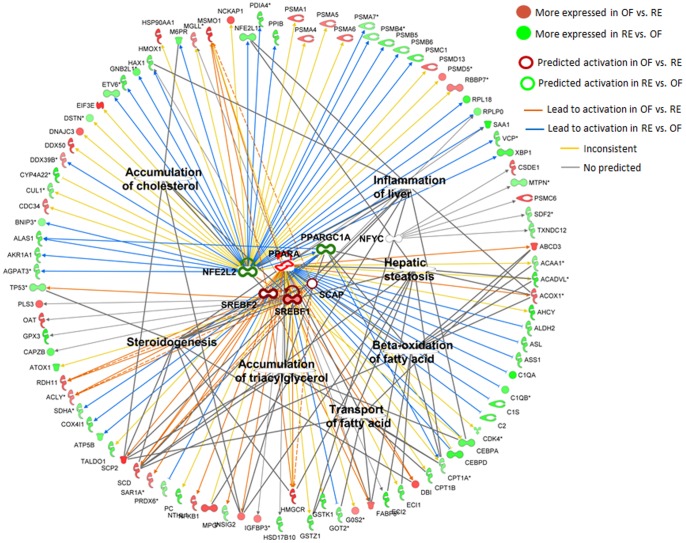
Ingenuity Pathway upstream network analysis of differentially expressed genes (DEG) between OF and RE at −14 d. Up-stream regulators are located at the center of the network and down-stream genes are located in the periphery. In the network are also reported the most enriched biological terms among down-stream genes.

The analysis of up-stream regulators among the DEG in the comparison of RE vs OF at −14, +1, and +14 d surprisingly revealed few gene targets overall and a higher number in the comparison on −14 d (Figure 6) compared with other time points (Figures S3 and S4). Among up-stream regulators at −14 d, several are related to lipid metabolism including PPARA, SREBF1, SREBF2, and SCAP, all more activated in OF vs. RE. The enrichment analysis revealed significant associations of down-stream genes with functions related to lipid metabolism and inflammation (Figure 6), among these ‘Hepatic steatosis’. Remarkably, an obvious increase in lipid accumulation in liver of OF cows was not evident until ca. 28 days later (i.e., +14 d) [10]. The transcription factor NFE2L2 was also uncovered to be an important up-stream regulator of the DEG between OF and RE at −14 d (Figure 6). The NFE2L2 is crucial regulator of the proper oxidative stress response and detoxification in liver [62]. This transcription factor was among the most important up-stream regulators also among DEG at +1 and +14 d (Figures S3 and S4). Interestingly, our data suggested that the higher liver capacity to respond to oxidative stress (including ER stress) and detoxification in RE vs. OF cows was more pronounced early post-partum compared to pre-partum (Figures 3 and 5). These data further support the idea that dietary energy prepartum “primed” the liver for a postpartum metabolic response.

Surprisingly, few up-stream regulators were uncovered by IPA at +1 d, with enrichment of lipid metabolism and proliferation related down-stream genes (Figure S3). At +14 d even fewer predicted up-stream regulators (and down-stream genes) were uncovered by IPA but there was an apparent prominent role and activation in OF vs. RE of osteopontin (SPP1) and metallopeptidase inhibitor 1 (TIMP1) both of which are related to induction of ECM formation [63], [64].

## Conclusions

**Figure 7:**
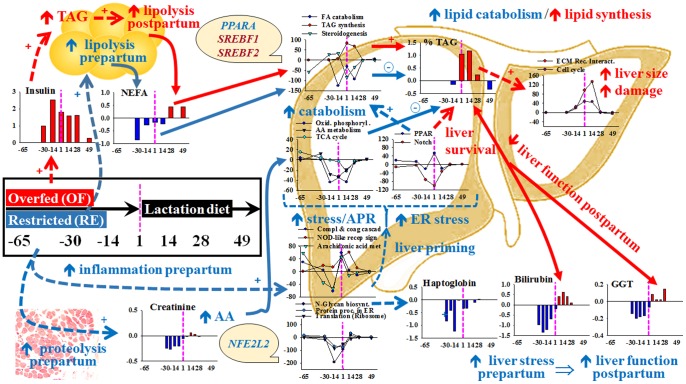
All-encompassing dynamic model proposed based on the main findings from the study. Reported are differential effects of the diet prepartum on adipose tissue, muscle, and liver (depicted by yellow round cells, image of a muscle section, and draw of a stylized liver, respectively). Red (text, lines, symbols and bars in graphs, and arrows) denotes facts that are more pronounced in cows overfed [OF] compared to cows underfed [RE] energy prepartum. Dark blue denotes facts that are more prominent in cows fed energy restricted diet prepartum compared to cows overfed energy prepartum. Data from representative plasma parameters are indicated by bars graphs with fold differences in overfed vs. restricted energy prepartum cows in Y-axis. Data from representative pathways from the Dynamic Impact Approach are shown as lines and scatter plots with the Direction of the Impact in Y-axis. In all cases the X-axis denotes the day relative to parturition. The dotted vertical purple line in all graphs denotes the end of treatment coinciding also with parturition. Solid lines and arrows denote flow of molecules/metabolites. Dotted lines and arrows denote effect. In all cases ⊕ denotes larger activation/amount and ⊖ denote larger inhibition. In text 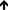/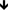 denote larger or lower in the treatment indicated by the font color (red  =  overfed and blue  =  feed restricted) compared to the other treatment. In the light-yellow background round shapes are reported main transcriptional factors potentially involved in controlling the transcriptomics adaptation of the indicated pathways. Summary explanation of the model is reported in the conclusion section of the paper.

The use of additional blood biomarkers and novel bioinformatics tools for the analysis of published transcriptomics data provided a more holistic understanding of the role of plane of nutrition during late-pregnancy on hepatic adaptations around the period of parturition. An all-encompassing dynamic model using these data is reported in Figure 7. Overfeeding energy prepartum enhances body fat deposition, partly in response to chronic hyperinsulinemia, which leads to more pronounced and sustained increase in blood NEFA postpartum and greater TAG accumulation in liver at least in part by reducing lipid catabolism and partly due to “dampened” PPARα activation. Despite such response, in overfed cows there was an attempt to counterbalance these negative effects by reducing Notch signaling and activating other cellular pathways of which cell cycle and ECM receptor interaction would likely help the liver repair from cellular damage (suggested by higher blood bilirubin and, numerically, GGT). On the contrary, although cows fed restricted energy appeared to catabolize substantially more muscle mass prepartum, their liver was able to adapt to the higher postpartal metabolic state well-ahead of parturition. This adaptation was likely driven by molecular processes partly controlled by transcription regulators such as PPARA and NFE2L2, of importance in fatty acid oxidation and cellular stress. As a result, restricted-fed cows had signs of greater metabolic flux and utilization of amino acids and fatty acids but also of a more pronounced cellular inflammatory and ER-stress response. Most of those cellular adaptations were confirmed by biomarker analysis specifically during the prepartal period, which strengthened the notion that restricted-energy helped “prime” the liver to cope with the change in physiological state at the onset of lactation.

Clearly, there is a carryover effect of plane of nutrition during late-pregnancy that will result in molecular and physiological adaptations during lactation. Our data support the view of a more robust liver in restricted-fed cows to face the metabolic and inflammatory challenges typical of the early postpartal period. As such, the transcriptomics data provide evidence that plane of dietary energy during late-pregnancy can help prime the liver for the onset of lactation.

## Methods

### Experimental design and ethic statement

All procedures were conducted under protocols approved by the University of Illinois Institutional Animal Care and Use Committee. The information about sampling, RNA extraction, and microarray data were published in the original study [10]. Briefly, the data used for this manuscript are from a subset of 8 Holstein dairy cows randomly selected from a larger study [5] fed either a higher-energy diet ad-libitum (>150% of net energy requirements; n = 4; OF) or fed a restricted energy diet (80% of net energy requirements; n = 4; RE) during the entire non-lactating period (∼last 65 days prior to parturition). For both groups of cows, the same level of energy and protein in the diet was provided from the day of parturition until the end of the study. The liver biopsies were harvested on day −65, −30, −14, +1, +14, +28, +49 relative to parturition. The microarray data were deposited in the National Center for Biotechnology Information (NCBI) Gene Expression Omnibus (GEO) database (http://www.ncbi.nlm.nih.gov/gds) with accession number GSE3331.

### Blood profiling

A large metabolic profiling including 18 parameters was performed in plasma collected every 7 days from −28 to +28 day relative to parturition. Parameters measured were: the indexes of acute phase reaction, such as the positive acute phase protein haptoglobin and ceruloplasmin; the negative acute phase protein albumin, paraoxonase and the other index related to the negative acute phase protein reaction such as cholesterol, β-carotene, and vitamin A; bilirubin; the liver enzymes aspartate aminotransferase (AST or **GOT**) and γ- glutamyl transpeptidase (**GGT**); minerals (calcium, magnesium, zinc, and phosphorous); creatinine; and vitamin E. The concentration of total proteins was analyzed also previously [10] and the data were re-analyzed in the present study in order to calculate accurately the concentration of globulins which are estimated by the difference between total proteins and albumin. Analysis of all parameters was performed as previously described [14], [15].

### Statistical analysis

Microarray spots with median intensity ≥3 standard deviation above the median of the background and GenePix 6 flag >100 were applied as filters to ensure high quality data. A total of 106 microarrays were adjusted for dye and array effect (Loess normalization and array centering), duplicated spot intensities were not averaged and were subsequently used for statistical analysis. A mixed model with repeated measures was then fitted to the normalized log_2_-transformed adjusted ratios (sample/reference standard) using Proc MIXED (SAS, SAS Inst. Inc., Cary, NC). The model included the fixed effects of time (−65, −30, −14, +1, +14, +28, +49 d), diet (OF and RE), and interactions of time × diet. Cow was considered as a random effect. The *p*-values were adjusted for the number of genes tested using Benjamini and Hochberg's false discovery rate (FDR) [65] to account for multiple comparisons. Differences in relative gene expression were considered significant at an FDR-adjusted *p*≤0.05 for time × diet. A post-hoc *p*≤0.05 was considered significant between diets at each time point.

For metabolic profiling parameters normal distribution was assessed using the procedure UNIVARIATE of SAS. Data not normally distributed were transformed into log scale. Data points with studentised residuals ≥2.5, analyzed by the SAS procedure REGRESSION, were considered outliers and excluded from the subsequent analysis (a total of 6 data points were removed out of ca. 4,000). A MIXED model procedure with a spatial power as a covariance structure was used. The model included diet, time, and diet × time as fixed effects, with cows as random variable. Means between treatments and time point were separated using the PDIFF. Data were deemed to be significant if overall diet × time interaction was *p*≤0.05 and tendencies at *p*≤0.10. Single point comparisons were determined to be significantly different if *p*≤0.05.

### Dynamic Impact Approach (DIA) and Enrichment Analysis

The detailed methodology for data analysis using DIA was previously described [12]. Briefly, the whole dataset (File S1) with Entrez gene ID, fold-change and the significance (*p*-values) of gene expression between the two diets at each time point, plus the overall FDR adjusted *p*-values were uploaded into DIA. The Kyoto Encyclopedia of Genes and Genomes (**KEGG**) pathways and Gene Ontology (**GO**) biological process category database were used for functional analysis with the DIA. For all the analyses a minimum of 20% genes in the annotated microarray vs. whole genome was used. The GO results from DIA were summarized using REVIGO [66]. For this analysis the GO ID along with the values of the direction of the impact as calculated by DIA were uploaded in REVIGO with analysis performed separately between GO terms induced in OF vs. RE and the one induced in RE vs. OF for −14, +1, and +14 d, the time points with larger number of DEG.

For enrichment analysis, the lists of DEG (overall time × diet FDR<0.05 and *p*-value) plus the whole annotated microarray as background were uploaded into DAVID bioinformatics resource database (http://david.abcc.ncifcrf.gov/). The Multi-List File function in the functional annotation tool of DAVID was selected to upload separately the list of DEG more expressed in OF vs. RE and the ones more expressed in RE vs. OF for −14, +1, and +14 d only, due to the very low number of DEG in other time points. The results from the default selected databases in DAVID were downloaded using “Functional annotation chart” with an adjusted *p*-value ≤0.10 (i.e., EASE score, which is defined as a conservative adjustment to the Fisher exact probability see [67]).

### KEGG pathway visualization

The most impacted KEGG pathways from DIA and enrichment analysis results were visualized using KegArray tool (http://www.kegg.jp/kegg/download/kegtools.html). For this purpose, the expression ratios between OF and RE along with Entrez gene ID were used as an input.

### Up-stream transcription regulator analysis via Ingenuity Pathway Analysis (IPA)

In order to uncover the main up-stream regulators of the DEG we have taken advantage of the upstream regulator analysis in IPA. The analysis uses an IPA Knowledge base to predict the expected causal effects between up-stream regulators and targets (i.e., DEG). The analysis provides the more plausible prediction of the status of the upstream regulator (i.e., activated or inhibited) by computing an overlap *p*-value and an activation z-score. For this purpose the whole dataset with Entrez-Gene ID, FDR of the diet × time effect, expression ratio, and *p*-values between each comparison were uploaded into IPA. All the predicted upstream and their targets were visualized in a network using the network tool. The analysis was performed for DEG at −14, +1, and +14 d due to the number of DEG observed in these comparisons.

## Supporting Information

Figure S1 **Results of plasma parameters, not significantly affected by prepartum dietary energy (OF  =  overfed energy and protein and RE  =  restricted-fed energy and protein prepartum).**
(TIF)

Figure S2 **Expression of several genes coding for positive and negative acute phase proteins during the whole duration of the study.** * indicate significant difference in expression at each time point between cows fed restricted (RE) energy and protein or receiving a higher energy and protein diet (OF) prepartum. Color of * is related to the color of symbol and lines for the gene.
(TIF)

Figure S3 **Ingenuity Pathway upstream network analysis of differentially expressed genes (DEG) between liver of cows fed restricted (RE) energy and protein or receiving a higher energy and protein diet (OF) prepartum at +1 d.** Up-stream regulators are located at the center of the network and down-stream genes are located in the periphery. Indicated are also the most enriched biological terms in the network.
(TIF)

Figure S4 **Ingenuity Pathway upstream network analysis of differentially expressed genes (DEG) between liver of cows fed restricted (RE) energy and protein or receiving a higher energy and protein diet (OF) prepartum at +14 d.** Up-stream regulators are located at the center of the network and down-stream genes are located in the periphery.
(TIF)

File S1 **Overall dataset with annotation and results of the statistical analysis.**
(XLSX)

File S2 **All results from the Dynamic Impact Approach (DIA) analysis.** Reported are the results for the Kyoto Encyclopedia of Genes and Genomes (KEGG) pathways (containing the summary or overall pathways, details of each pathway, and pathways sorted by impact in each time point comparison) and Gene Ontology biological process (GOBP, both details and sorted by impact in each time point comparison).
(XLSX)

File S3 **KegArray (** http://www.genome.jp/kegg/expression/ **) results of several of the most affected KEGG pathways by prepartum energy level in the diet for the −14, 1, and 14 d comparison.**
(DOCX)

File S4 **REVIGO (** http://revigo.irb.hr/ **) summary of the Dynamic Impact Apporach (DIA) analysis of the Gene Ontology biological processes (GO BP) affected in liver by prepartum dietary energy.** The results are shown as Treemaps separated between terms more activated in OF vs. RE and the ones more activated in RE vs. OF. The dimension of each term is directly proportional to the overal induction. Same color indicate semantic and functional association.
(DOCX)

File S5 **Complete results from Database for Annotation, Visualization and Integrated Discovery (DAVID) of DEG between liver of cows fed restricted (RE) energy and protein or receiving a higher energy and protein diet (OF) prepaprtum.** The analysis was performed separately between genes more expressed in OF vs. RE and genes more expressed in RE vs. OF at −14, 1, and 14 d relative to parturition.
(XLSX)
