# Supplementary figures and images for: Integrative Analyses of Hepatic Differentially Expressed Genes and Blood Biomarkers during the Peripartal Period between Dairy Cows Overfed or Restricted-Fed Energy Prepartum
Source: PLoS One. 2014 Jun 10;9(6):e99757. doi: 10.1371/journal.pone.0099757 (PMC4051754; doi:10.1371/journal.pone.0099757)

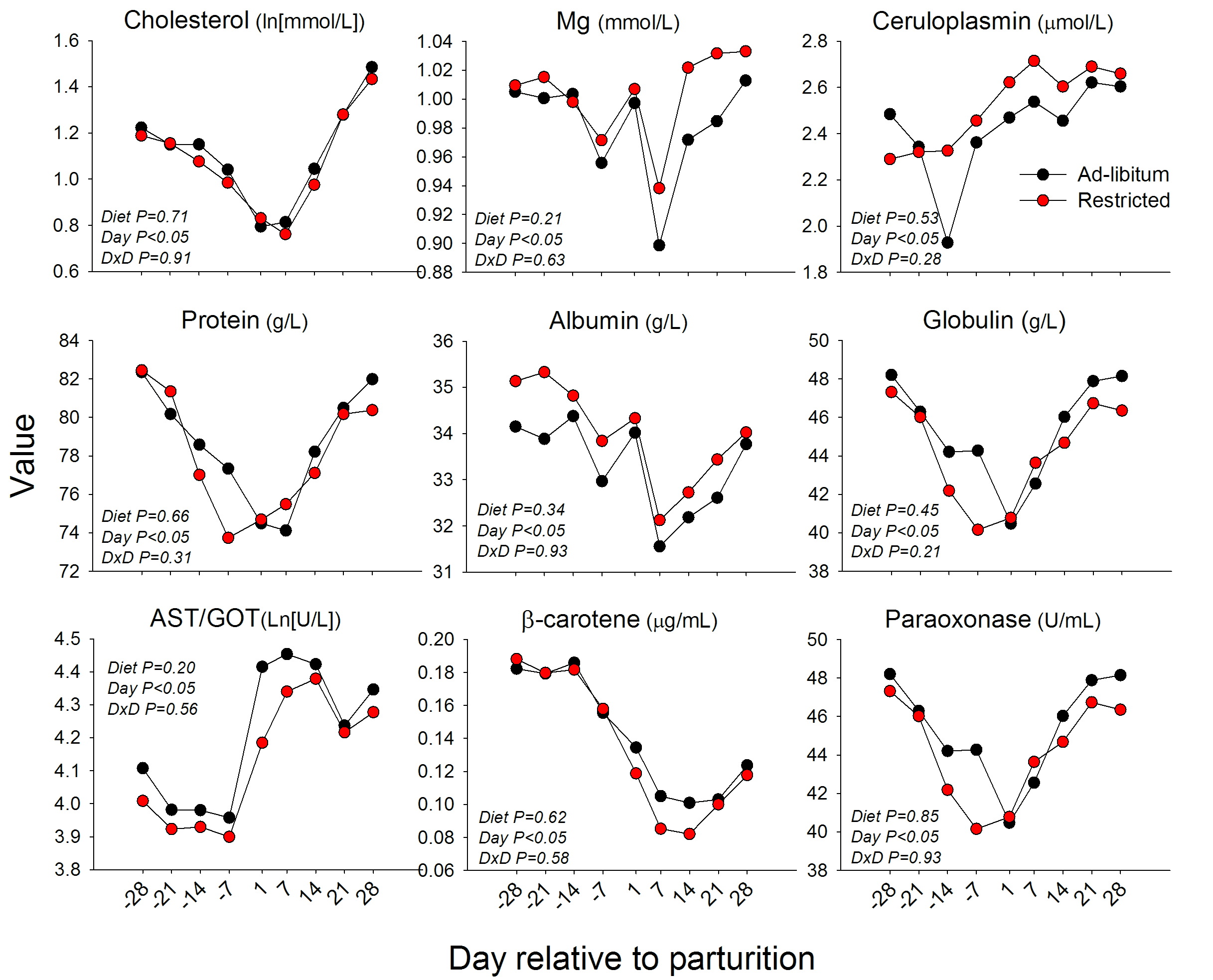

Supplement: Figure S1 — Results of plasma parameters, not significantly affected by prepartum dietary energy (OF = overfed energy and protein and RE = restricted-fed energy and protein prepartum). (TIF) [file pone.0099757.s001.tif]

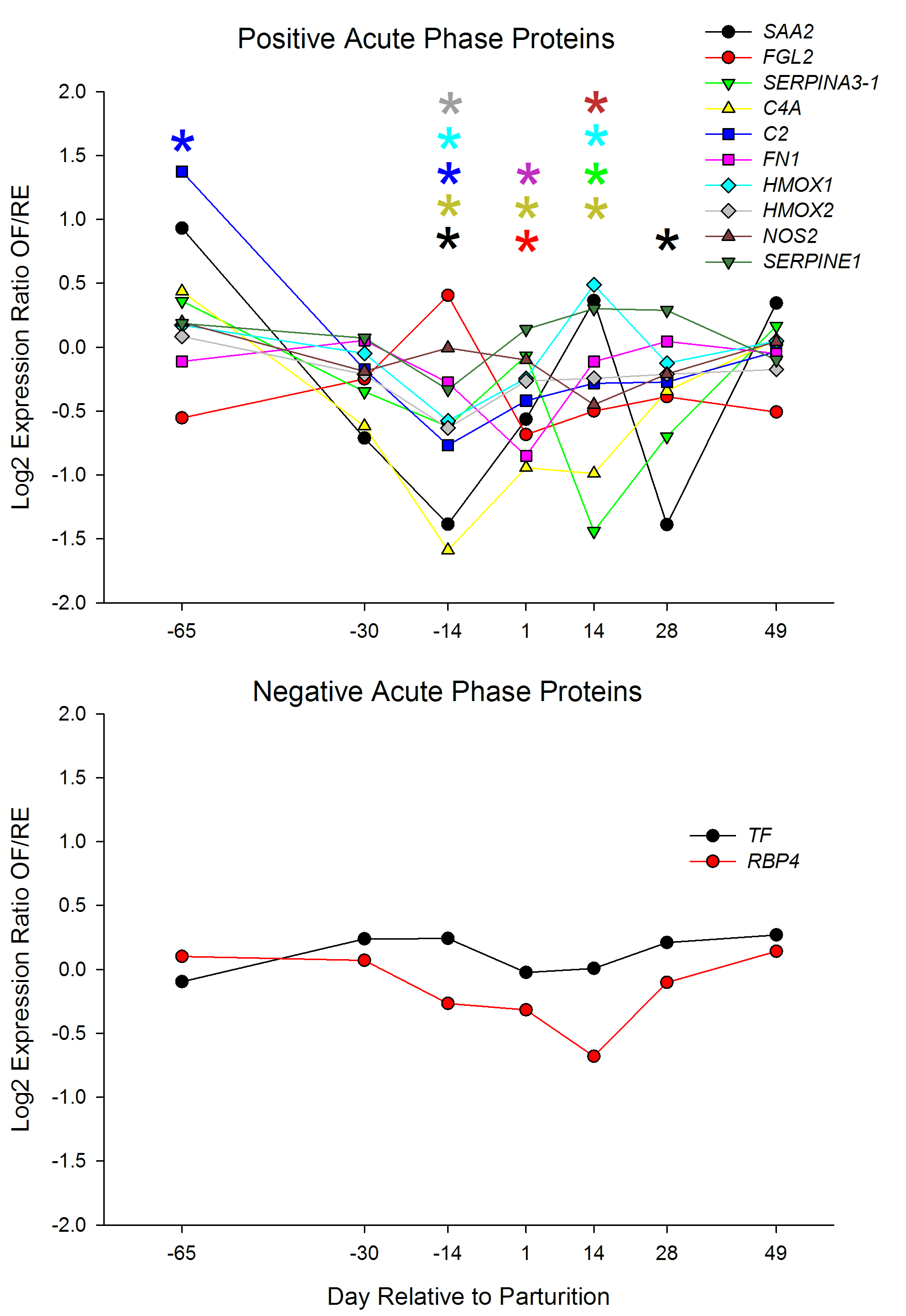

Supplement: Figure S2 — Expression of several genes coding for positive and negative acute phase proteins during the whole duration of the study. * indicate significant difference in expression at each time point between cows fed restricted (RE) energy and protein or receiving a higher energy and protein diet (OF) prepartum. Color of * is related to the color of symbol and lines for the gene. (TIF) [file pone.0099757.s002.tif]

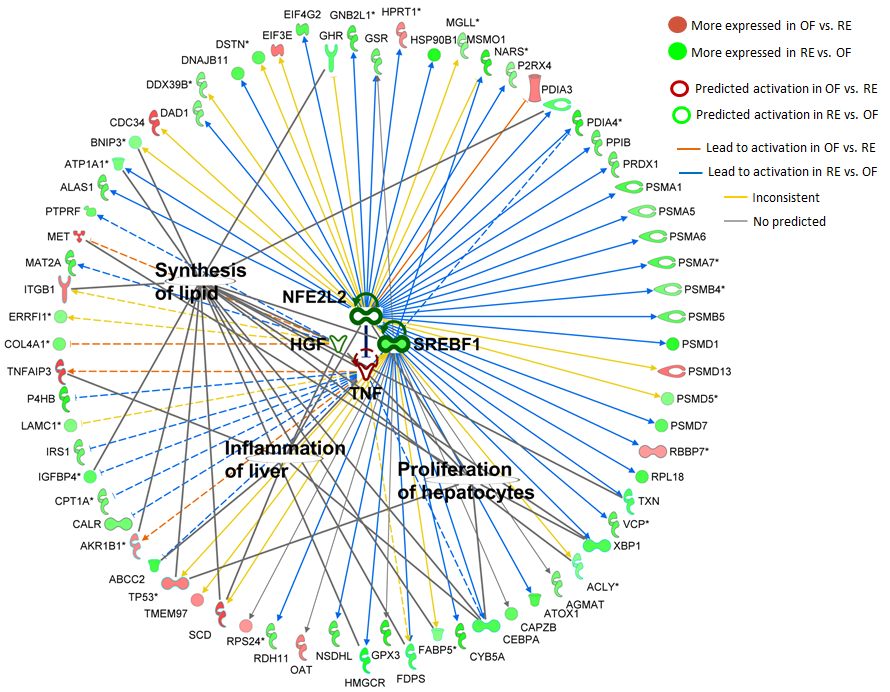

Supplement: Figure S3 — Ingenuity Pathway upstream network analysis of differentially expressed genes (DEG) between liver of cows fed restricted (RE) energy and protein or receiving a higher energy and protein diet (OF) prepartum at +1 d. Up-stream regulators are located at the center of the network and down-stream genes are located in the periphery. Indicated are also the most enriched biological terms in the network. (TIF) [file pone.0099757.s003.tif]

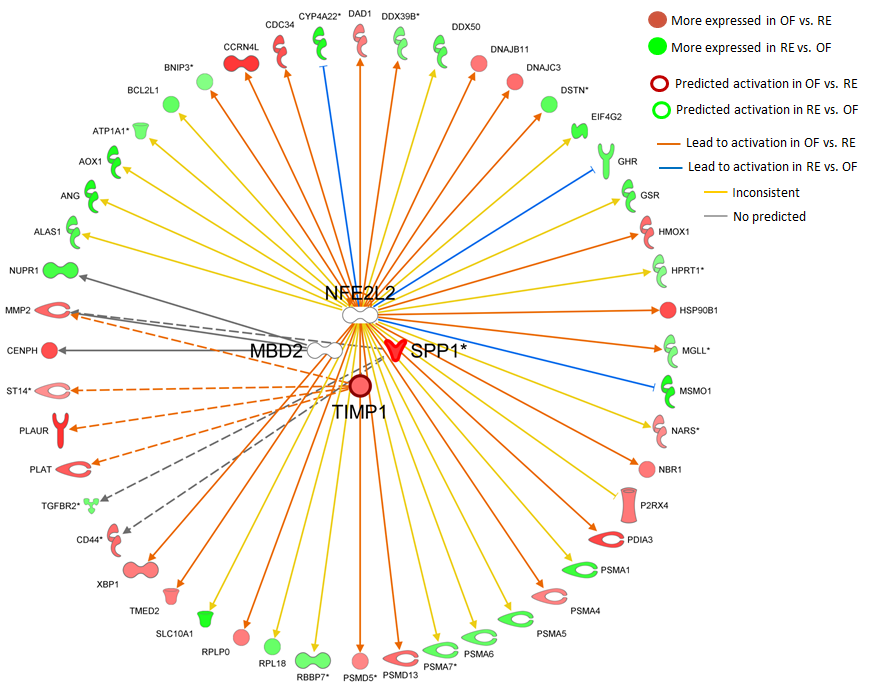

Supplement: Figure S4 — Ingenuity Pathway upstream network analysis of differentially expressed genes (DEG) between liver of cows fed restricted (RE) energy and protein or receiving a higher energy and protein diet (OF) prepartum at +14 d. Up-stream regulators are located at the center of the network and down-stream genes are located in the periphery. (TIF) [file pone.0099757.s004.tif]
