# Supplementary material for: Integrative Analyses of Hepatic Differentially Expressed Genes and Blood Biomarkers during the Peripartal Period between Dairy Cows Overfed or Restricted-Fed Energy Prepartum
Source: PLoS One. 2014 Jun 10;9(6):e99757. doi: 10.1371/journal.pone.0099757 (PMC4051754; doi:10.1371/journal.pone.0099757)
Supplement: File S3 — KegArray ( http://www.genome.jp/kegg/expression/ ) results of several of the most affected KEGG pathways by prepartum energy level in the diet for the −14, 1, and 14 d comparison. (DOCX) [file pone.0099757.s007.docx]

**VISUALIZATION OF KEGG PATHWAYS**

KEGG pathways visualization for the **comparison OF vs. RE cows** using the application **KegArray** available in KEGG: Kyoto Encyclopedia of Genes and Genomes website at <http://www.genome.jp/kegg/download/kegtools.html>. The gene IDs of pathways from -14 to 14 days with fold change values were uploaded to the KEGG database using the KegArray tool.

The results from analysis using KegArray is not fully comparable with the DIA because the former only use the fold change as input while the DIA accounts for proportion of DEG compared to the genes present in array, the P-value of the change, and the fold change/difference. In addition, the DIA does not weight more the down-regulated genes compared to the up-regulated, but the KegArray tends to use the “limit enzyme” concept in the pathway, i.e., if in a enzymatic complex all genes coding for the proteins are up-regulated but one gene is down-regulated the object of the complex will appear green.

The orange-red object denote protein (or complex of proteins) overall more expressed in OF vs. RE, the green object denote protein (or complex of proteins) overall more expressed in RE vs. OF, the grey objects denote genes present (or annotated) in the bovine genome but not affected by diet or not present in the microarray, and white objects denote genes not yet annotated in the bovine genome. Shown are only the pathways from -14 to 14 days; these pathways are shown in descending order of impact as calculated by the DIA.

Table of Contents

[Metabolic Pathways 5](#_Toc371324837)

[-14 DAYS 5](#_Toc371324838)

[1 DAY 6](#_Toc371324839)

[14 DAYS 7](#_Toc371324840)

[1. Metabolism 8](#_Toc371324841)

[1.1 Carbohydrate Metabolism 8](#_Toc371324842)

[Pyruvate Metabolism 8](#_Toc371324843)

[Fructose and mannose Metabolism 9](#_Toc371324844)

[Galactose Metabolism 10](#_Toc371324845)

[Glycolysis / Gluconeogenesis 11](#_Toc371324846)

[Citrate Cycle (TCA Cycle) 13](#_Toc371324847)

[Propanoate Metabolism 14](#_Toc371324848)

[1.2 Energy Metabolism 15](#_Toc371324849)

[Oxidative Phosphorylation 15](#_Toc371324850)

[1.3 Lipid Metabolism 16](#_Toc371324851)

[Glycerolipid Metabolism 16](#_Toc371324852)

[Arachidonic Acid Metabolism 17](#_Toc371324853)

[Synthesis and Degradation of Ketone Bodies 18](#_Toc371324854)

[Fatty acid Metabolism 19](#_Toc371324855)

[1.5 Amino Acid Metabolism 20](#_Toc371324856)

[Alanine, Aspartate and Glutamate metabolism 20](#_Toc371324857)

[Valine, leucine and isoleucine degradation 21](#_Toc371324858)

[1.7 Glycan biosynthesis and metabolism 23](#_Toc371324859)

[N-Glycan Biosynthesis 23](#_Toc371324860)

[1.8 Metabolism of Cofactors and Vitamins 24](#_Toc371324861)

[Ubiquinone (coenzyme Q) and other terpenoid-quinone biosynthesis 24](#_Toc371324862)

[Folate Biosynthesis 25](#_Toc371324863)

[1.9 Metabolism of Terpenoids and Polyketides 26](#_Toc371324864)

[Terpenoid backbone biosynthesis 26](#_Toc371324865)

[1.10 Biosynthesis of other secondary metabolites 27](#_Toc371324866)

[Caffeine metabolism 27](#_Toc371324867)

[2. Genetic Information processing 28](#_Toc371324868)

[2.1 Transcription 28](#_Toc371324869)

[Basal Transcription factors 28](#_Toc371324870)

[2.2 Translation 29](#_Toc371324871)

[RNA Transport 29](#_Toc371324872)

[Ribosome 30](#_Toc371324873)

[mRNA Surveillance Pathway 31](#_Toc371324874)

[2.3 Folding, Sorting and Degradation 32](#_Toc371324875)

[Protein Export 32](#_Toc371324876)

[Protein Processing in Endoplasmic Reticulum 33](#_Toc371324877)

[Ubiquitin Mediated Proteolysis 34](#_Toc371324878)

[2.4 Replication and Repair 35](#_Toc371324879)

[Base Excision Repair 35](#_Toc371324880)

[Non-homologous end-joining 36](#_Toc371324881)

[3. Environmental Information Processing 37](#_Toc371324882)

[3.1 Membrane Transport 37](#_Toc371324883)

[ABC transporters 37](#_Toc371324884)

[3.2 Signal Transduction 38](#_Toc371324885)

[Notch signaling 38](#_Toc371324886)

[3.3 Signaling Molecules and Interaction 39](#_Toc371324887)

[ECM-receptor Interaction 39](#_Toc371324888)

[4. Cellular Processes 40](#_Toc371324889)

[4.1 Transport and catabolism 40](#_Toc371324890)

[Peroxisome 40](#_Toc371324891)

[Lysosome 41](#_Toc371324892)

[5. Organismal Systems 42](#_Toc371324893)

[5.1 Immune system 42](#_Toc371324894)

[Complement and coagulation cascade 42](#_Toc371324895)

[NOD-like receptor signaling pathway 43](#_Toc371324896)

[Antigen processing and presentation 44](#_Toc371324897)

[5.2. Endocrine system 45](#_Toc371324898)

[Renin-angiotensin system 45](#_Toc371324899)

[PPAR signaling pathway 46](#_Toc371324900)

# Metabolic Pathways

Orange line denote flux in pathways more induced in OF vs. RE cows, while light green lines denote flux in pathways more induced in RE vs. OF cows. Other color lines denote the overall category of pathways

## -14 DAYS


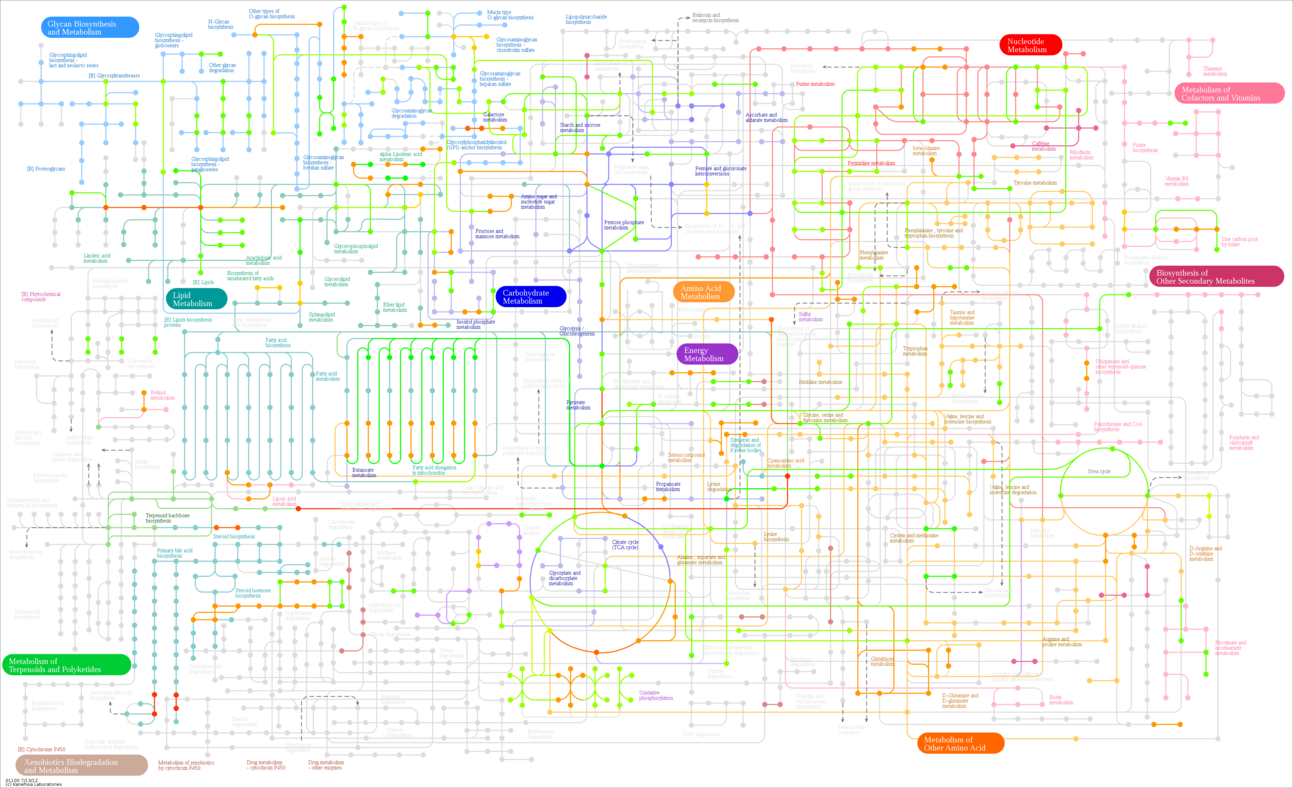


## 1 DAY


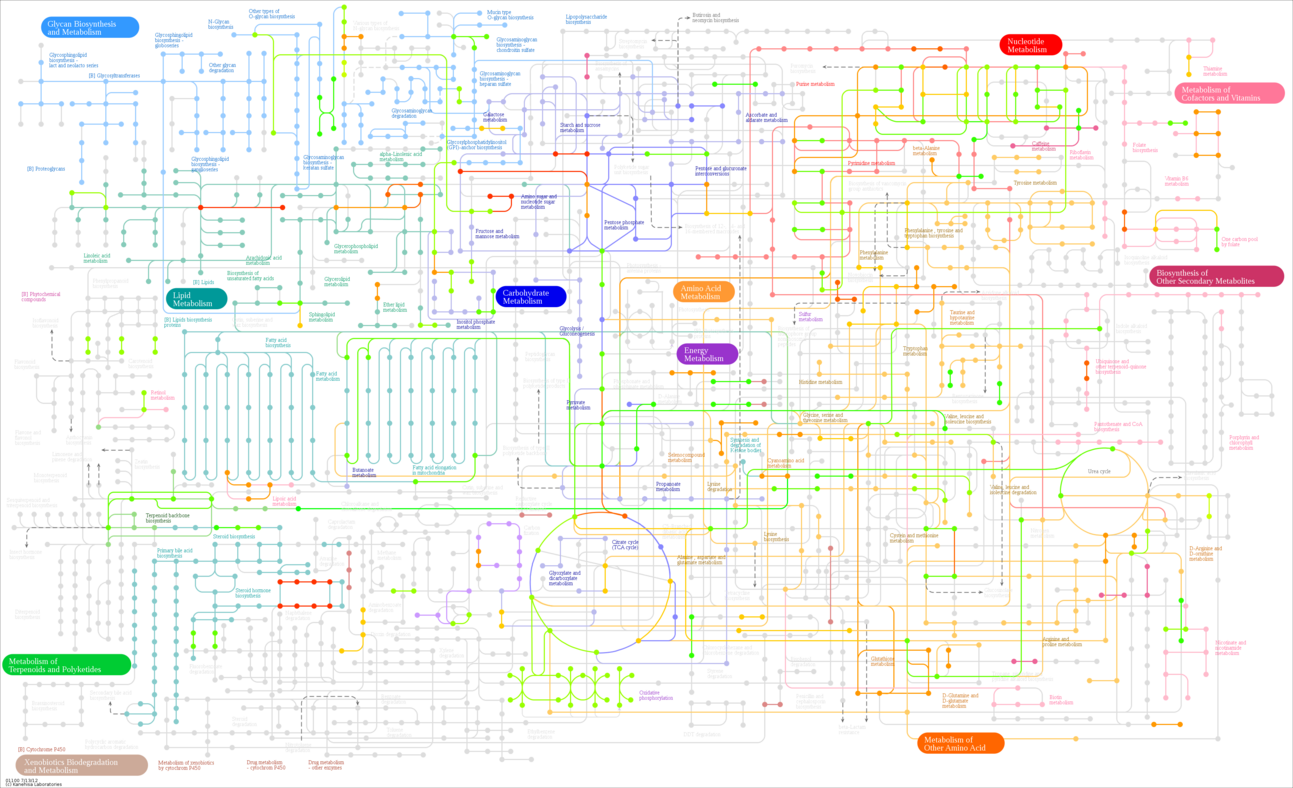


## 14 DAYS


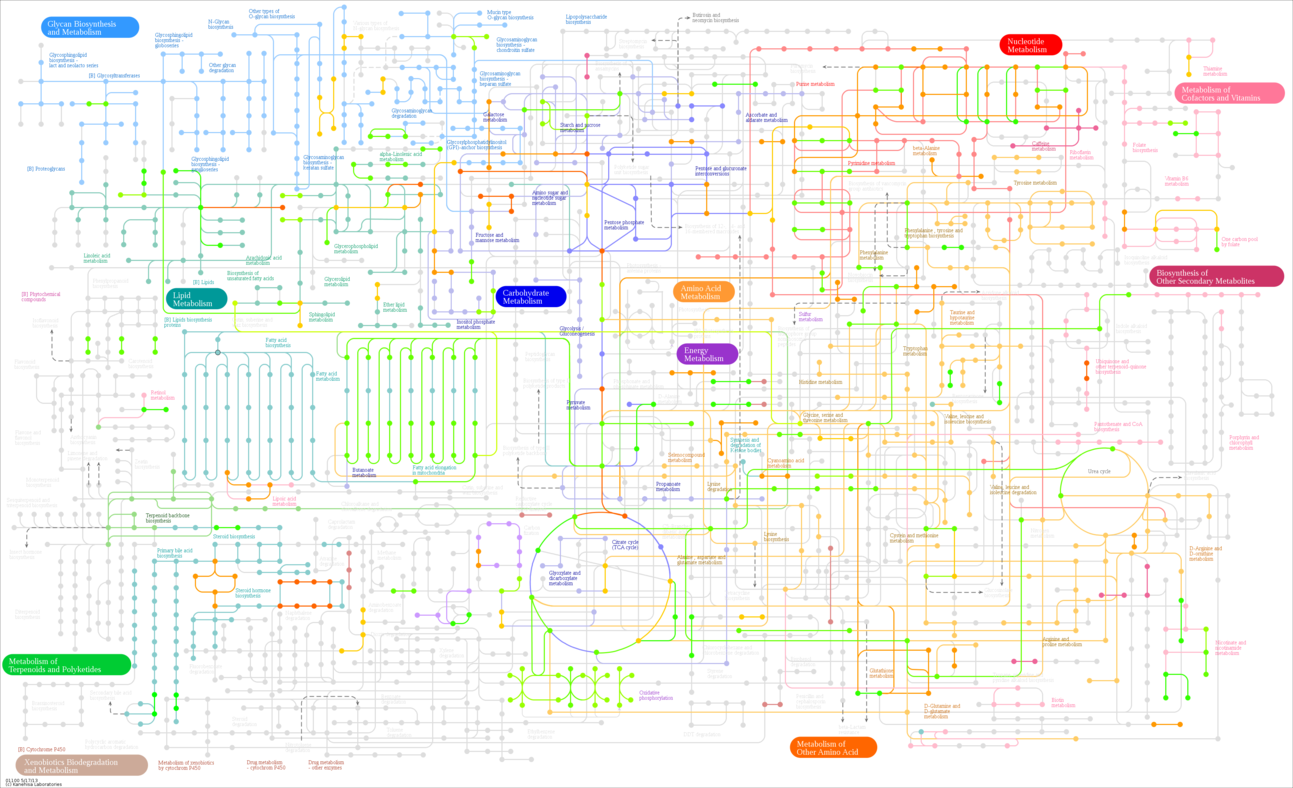


# 1. Metabolism

## 1.1 Carbohydrate Metabolism

### Pyruvate Metabolism

-14 DAYS


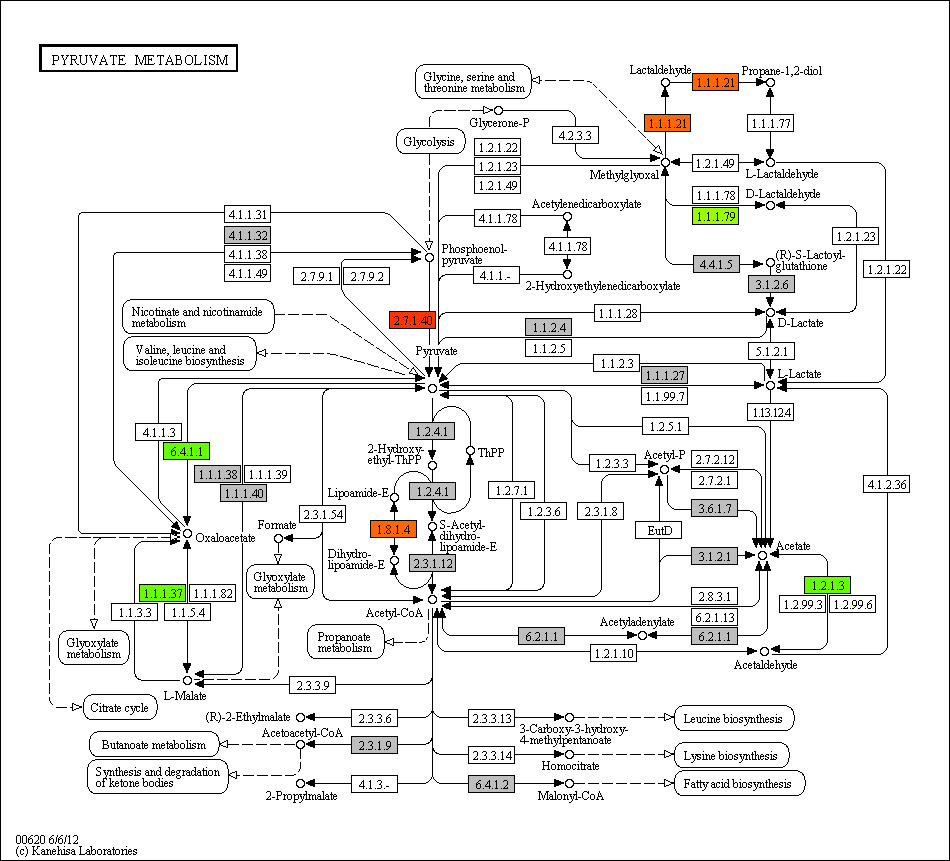


1 DAY 14 DAYS


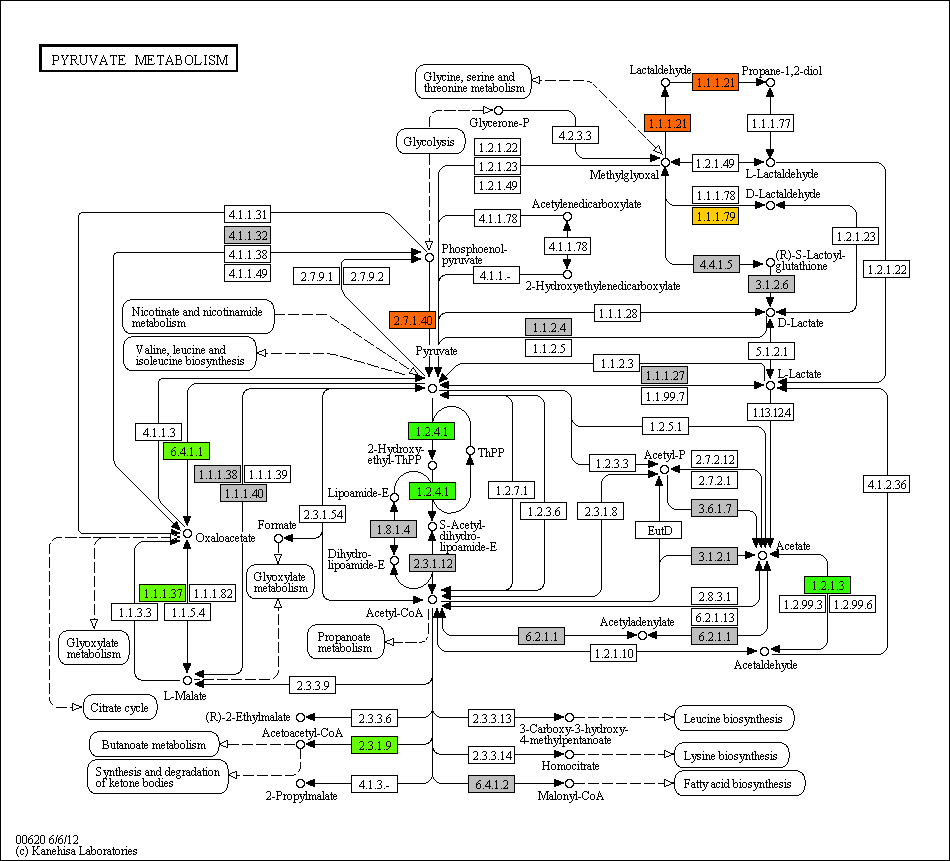

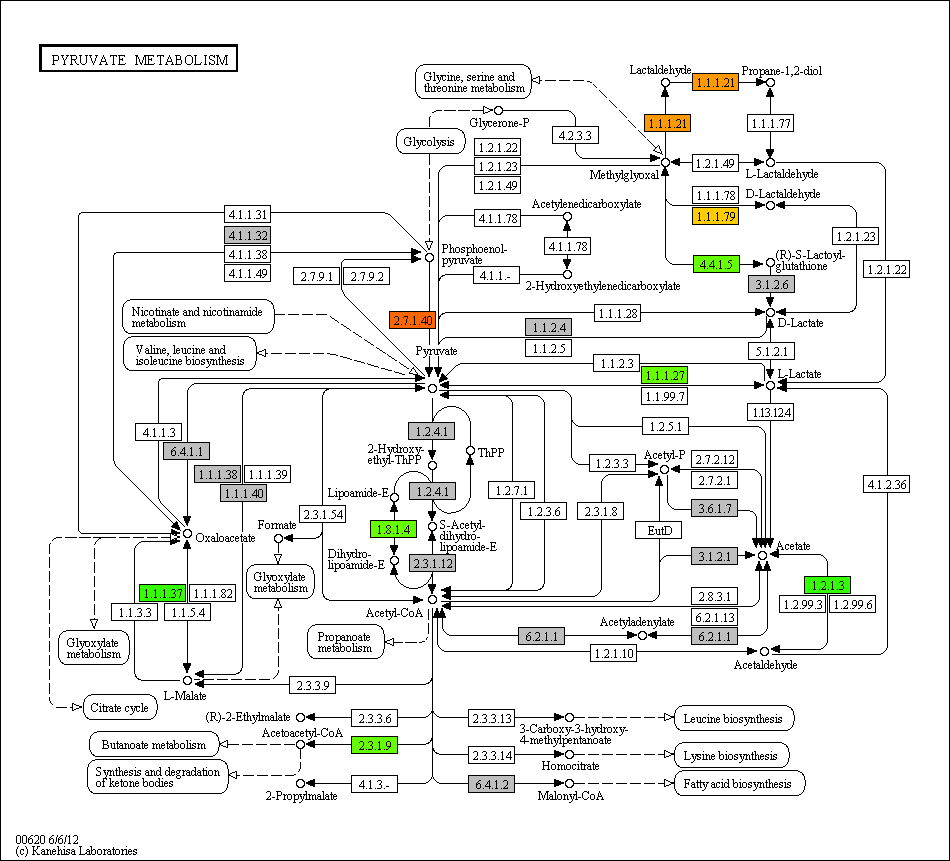


### Fructose and mannose Metabolism

-14 DAYS


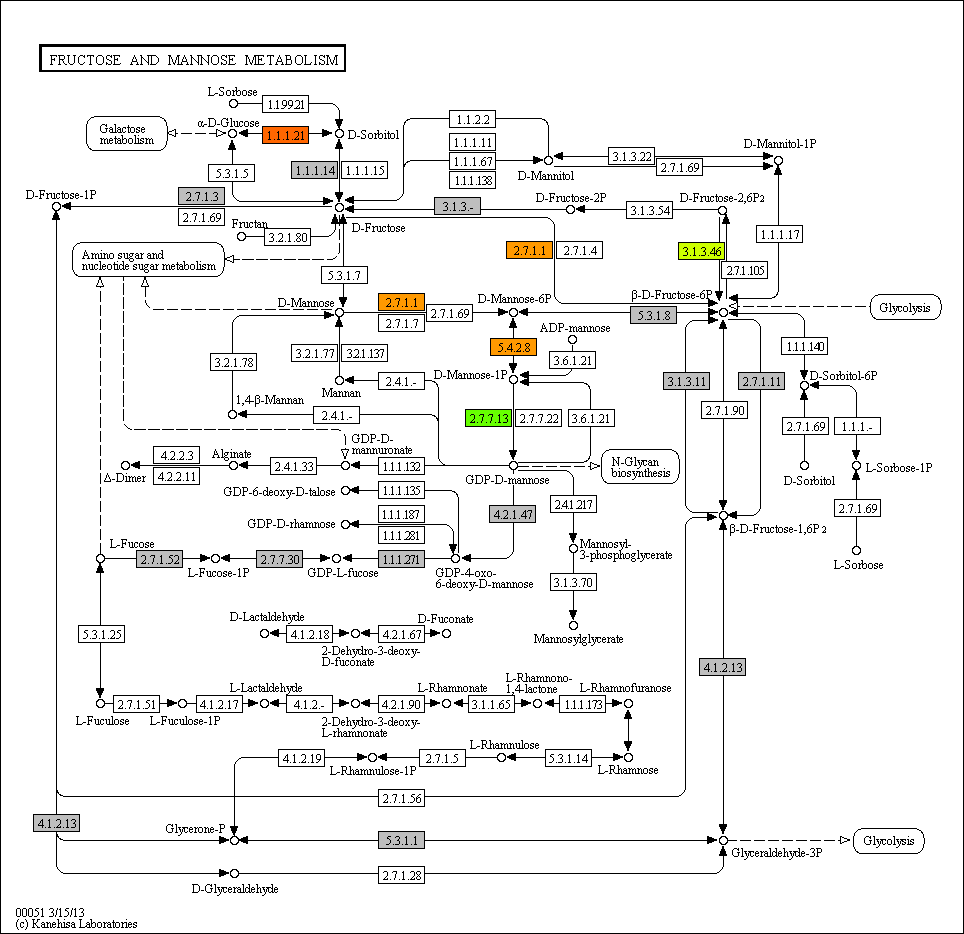


1 DAY 14 DAYS


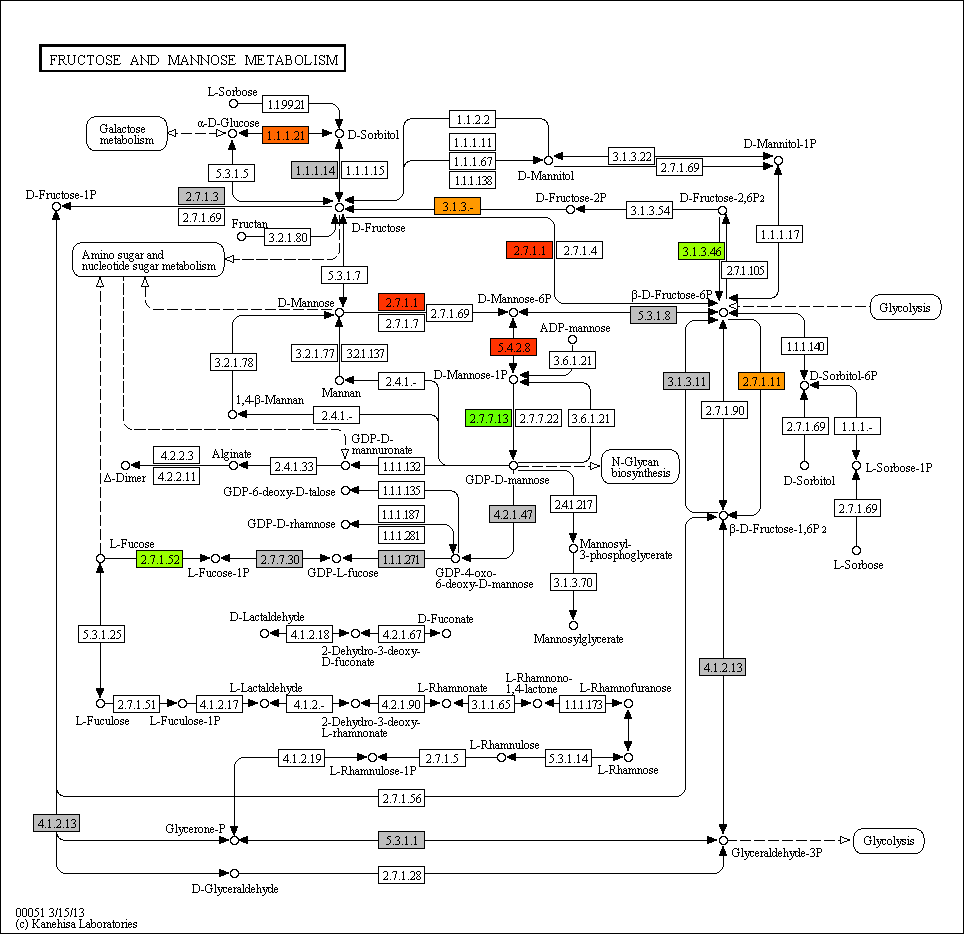

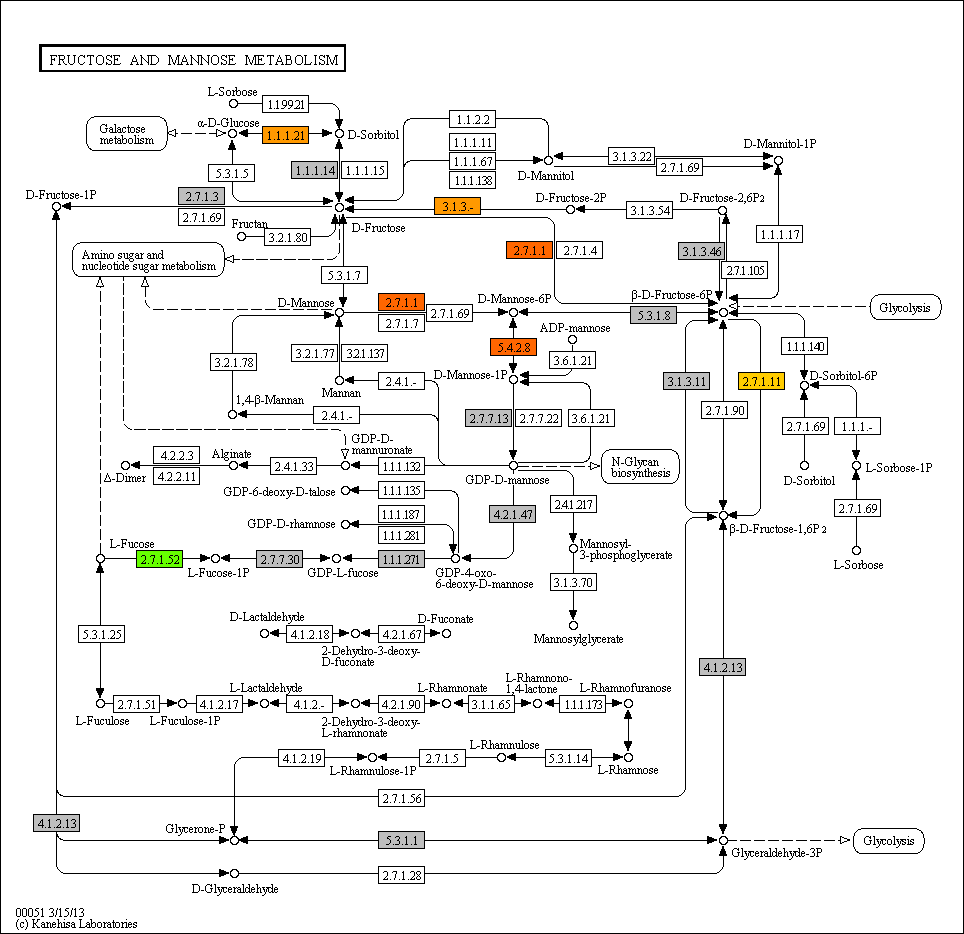


### Galactose Metabolism

-14 DAYS


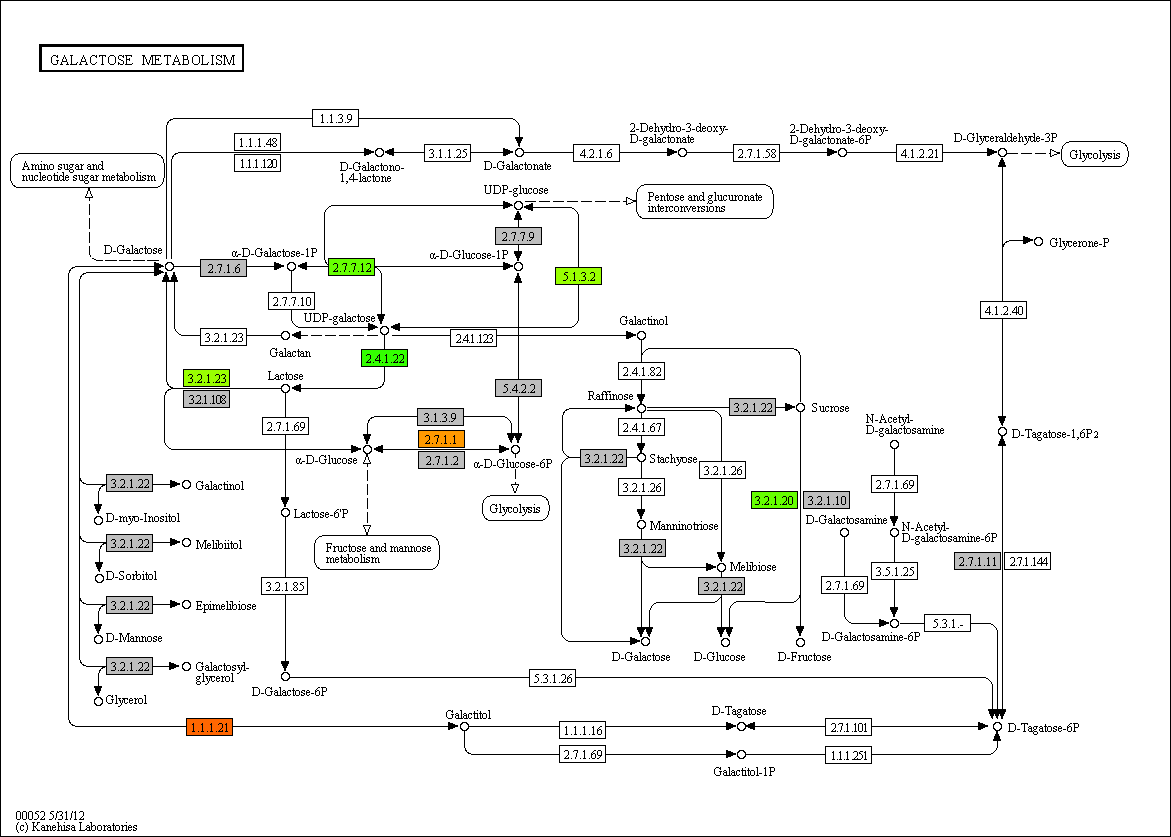


1 DAY 14 DAYS


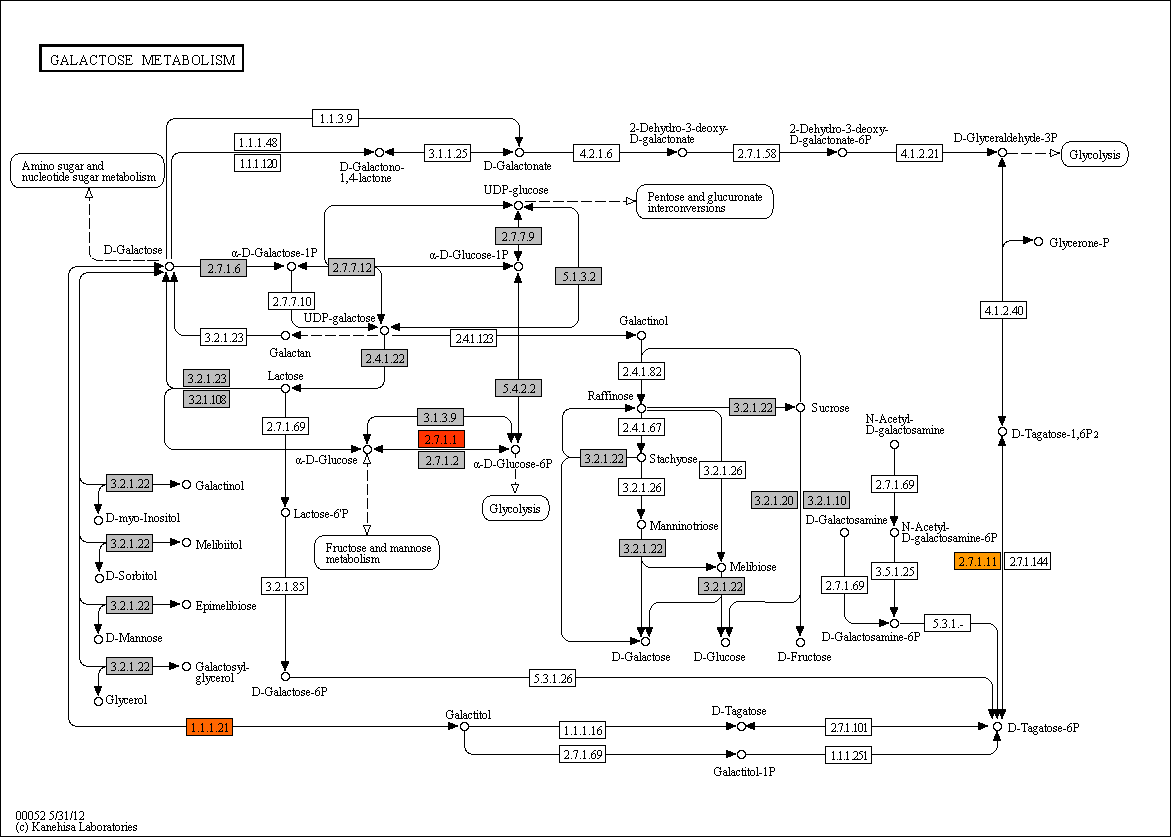

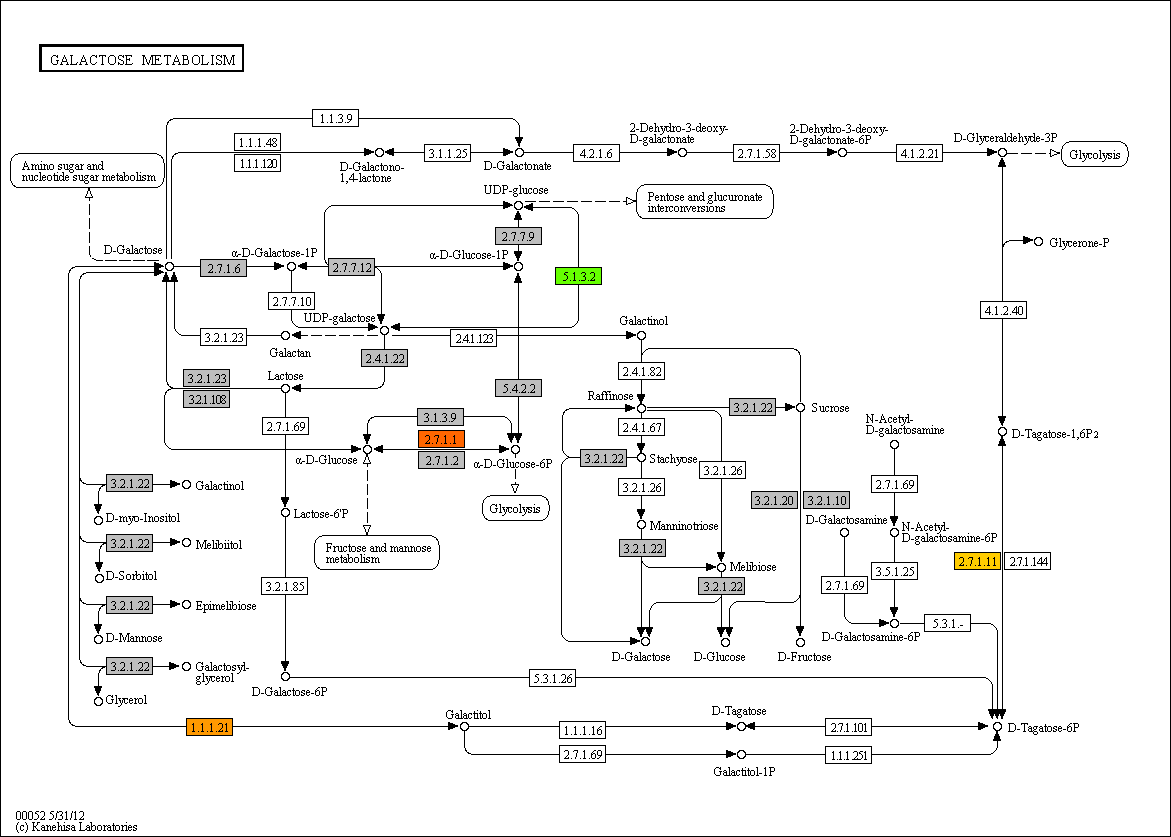


### Glycolysis / Gluconeogenesis

-14 DAYS 1 DAY


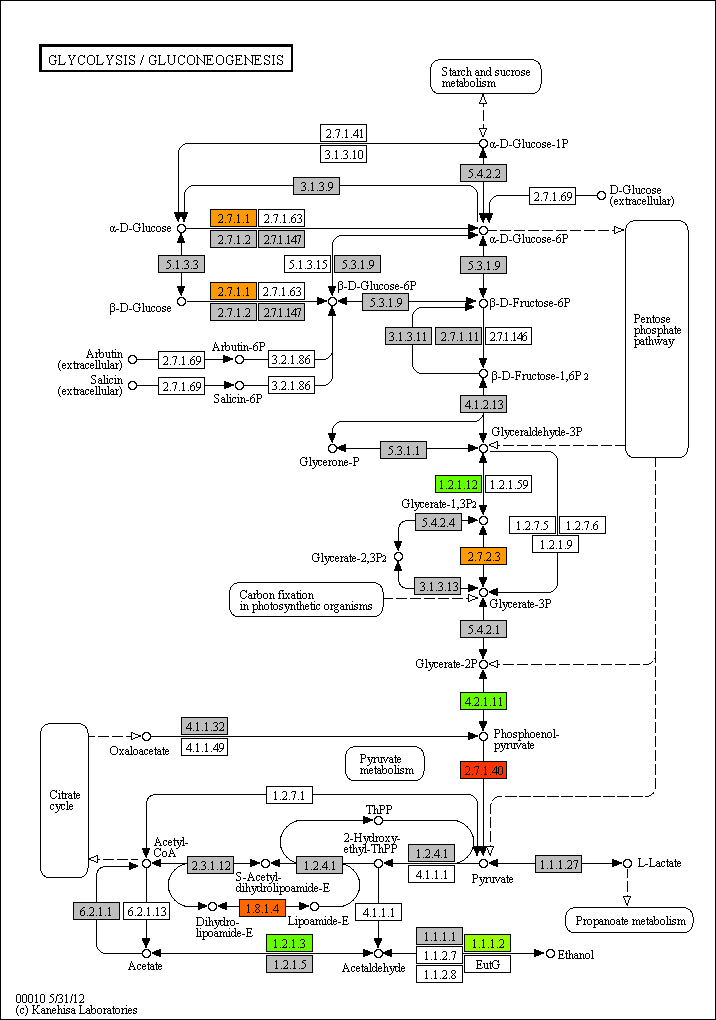

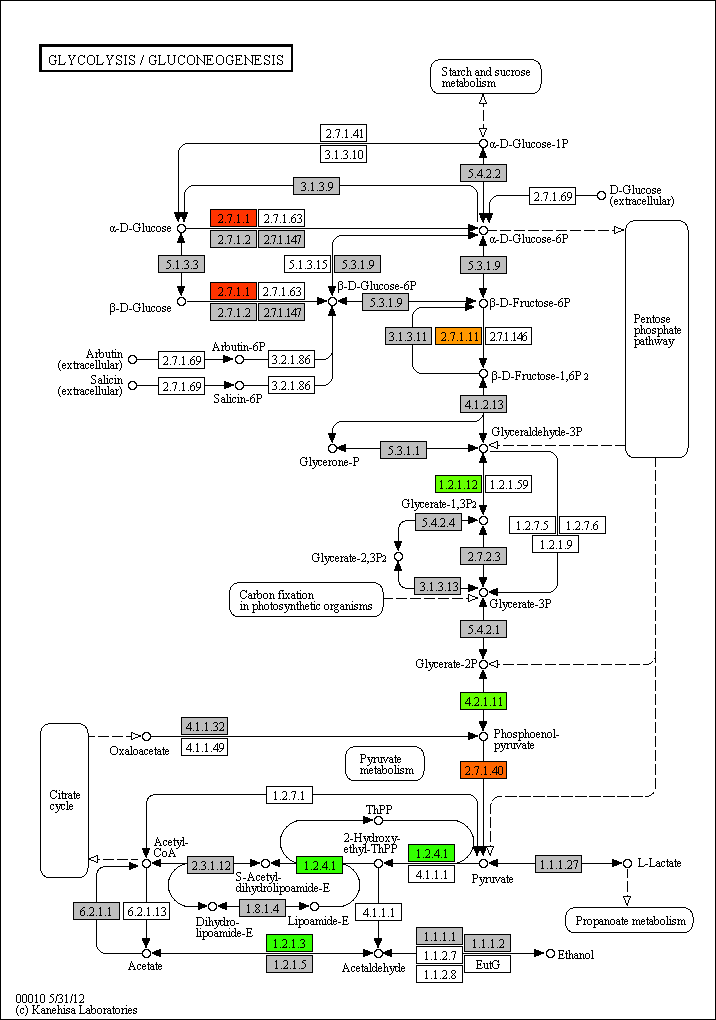


14 DAY


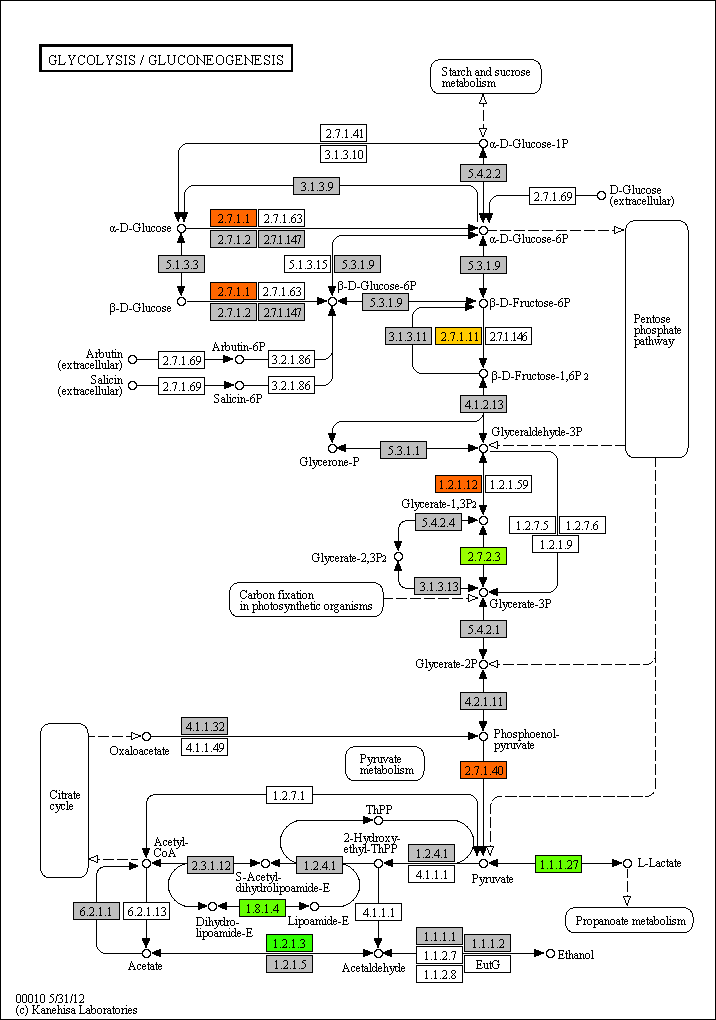


**Glyoxylate and dicarboxylate Metabolism**

-14 DAYS


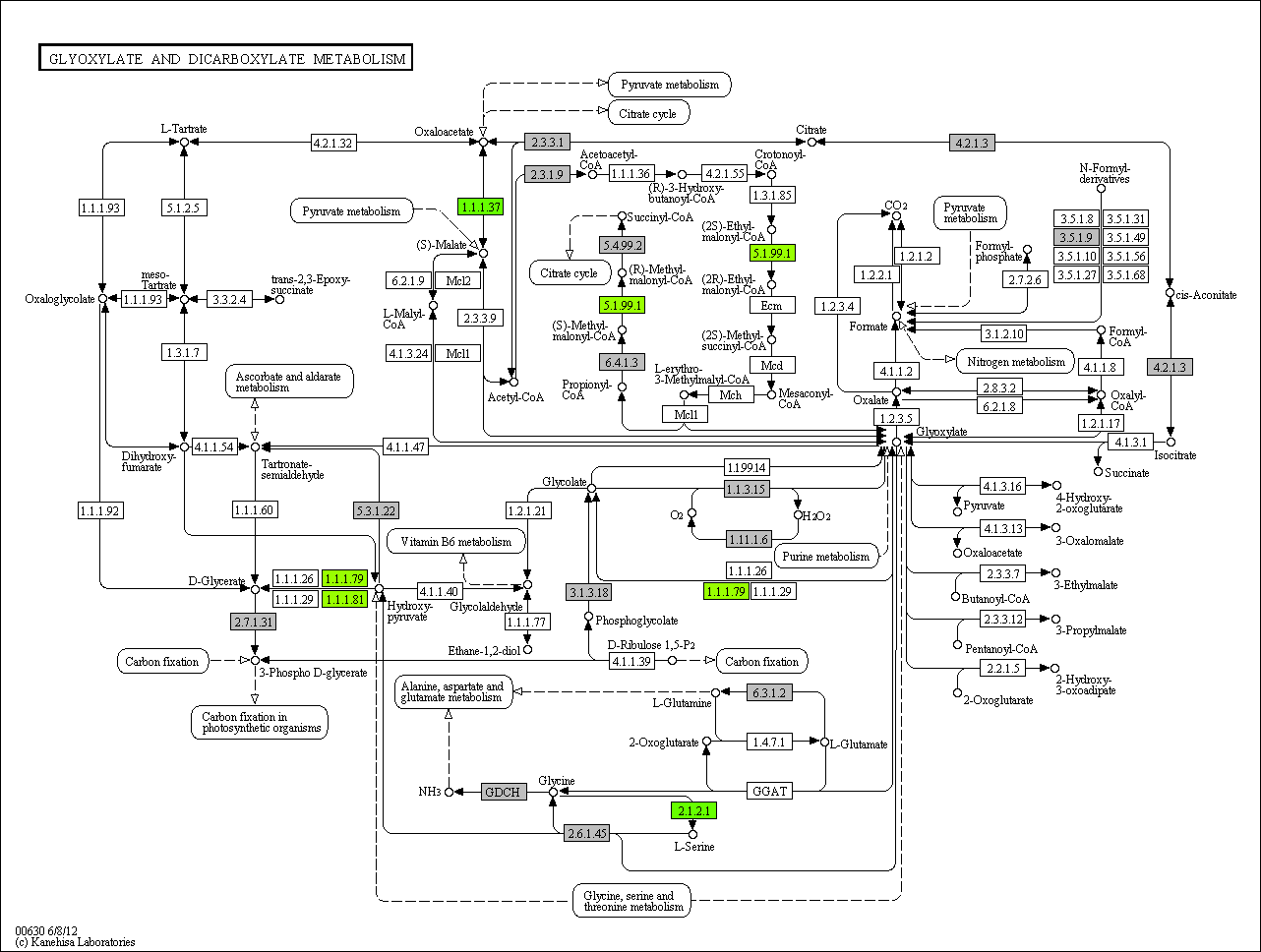


1 DAY 14 DAYS


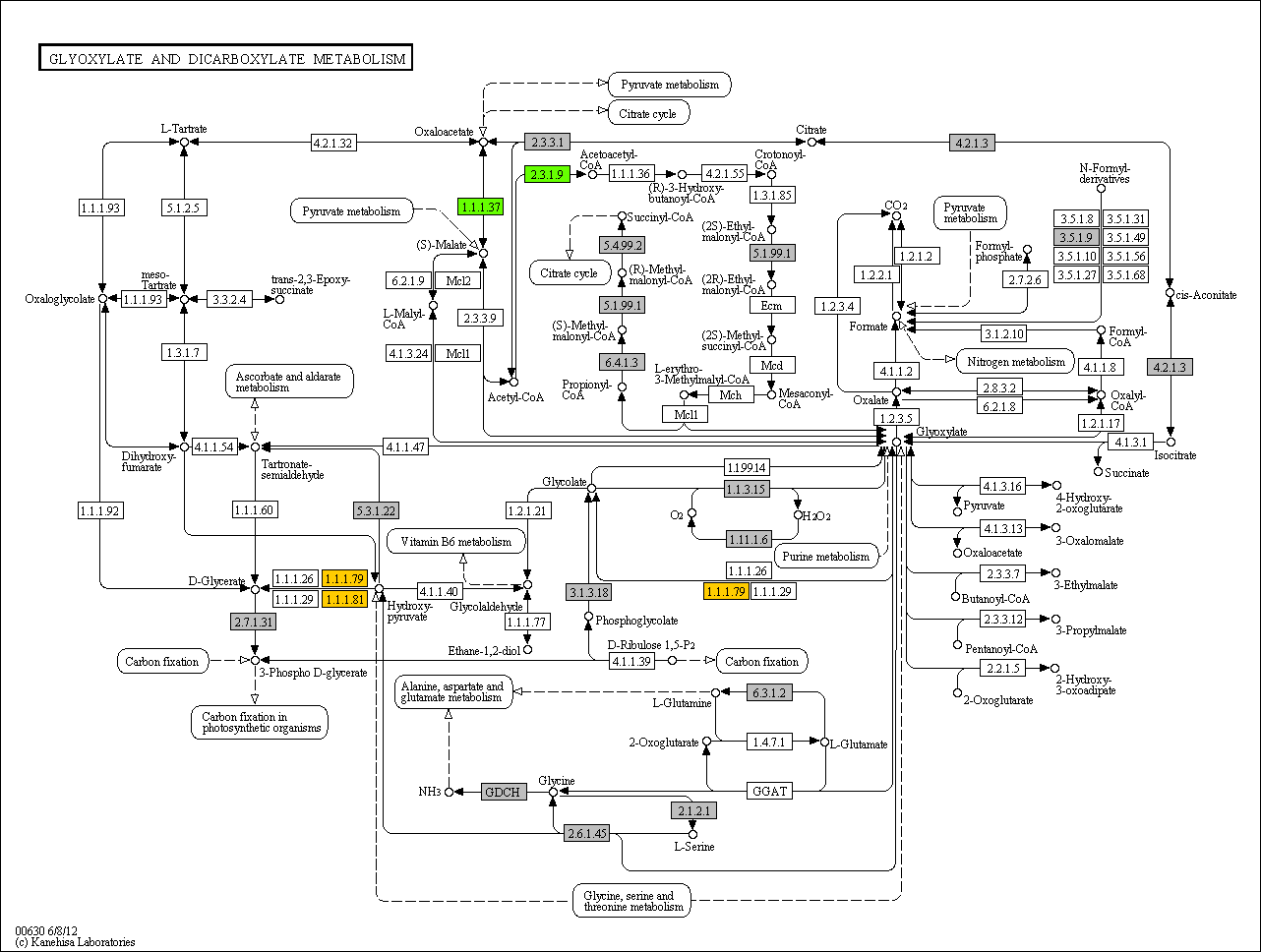

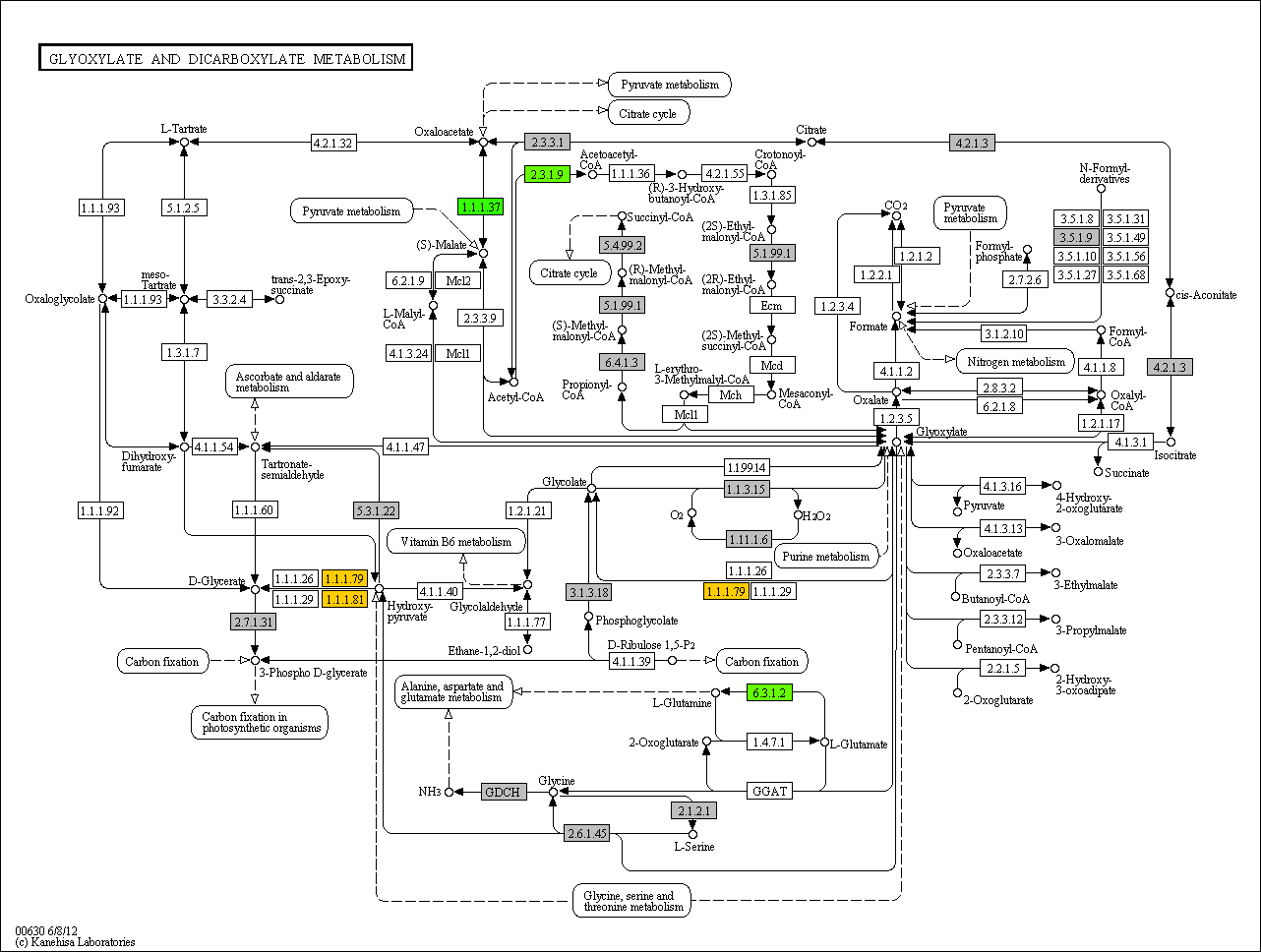


### Citrate Cycle (TCA Cycle)

-14 DAYS


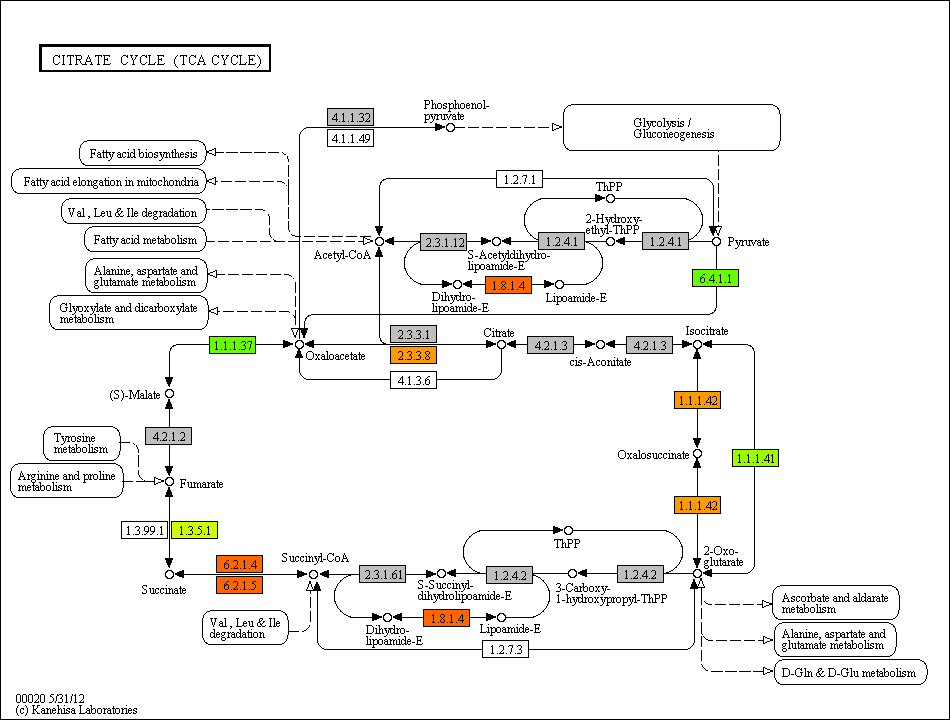


1 DAY 14 DAYS


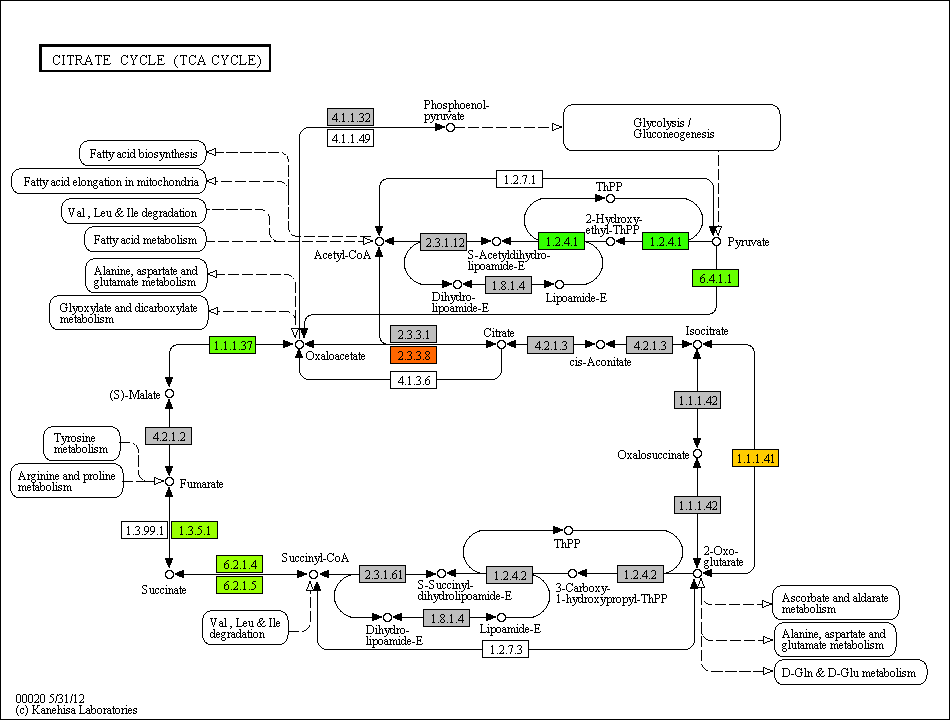

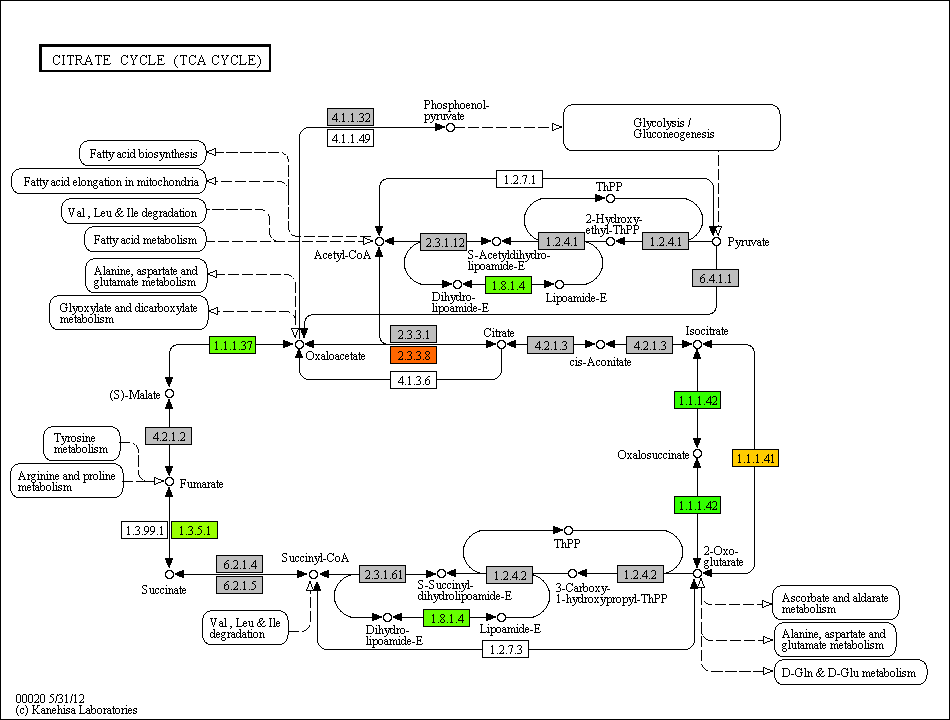


### Propanoate Metabolism

-14 DAYS


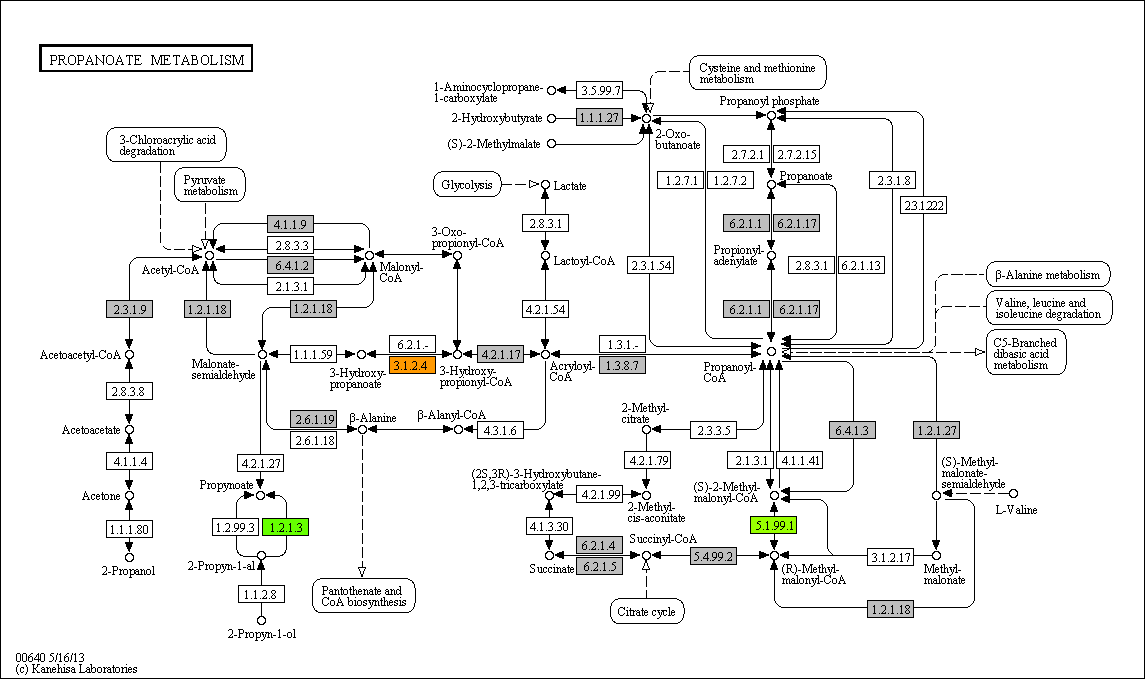


1 DAY 14 DAYS


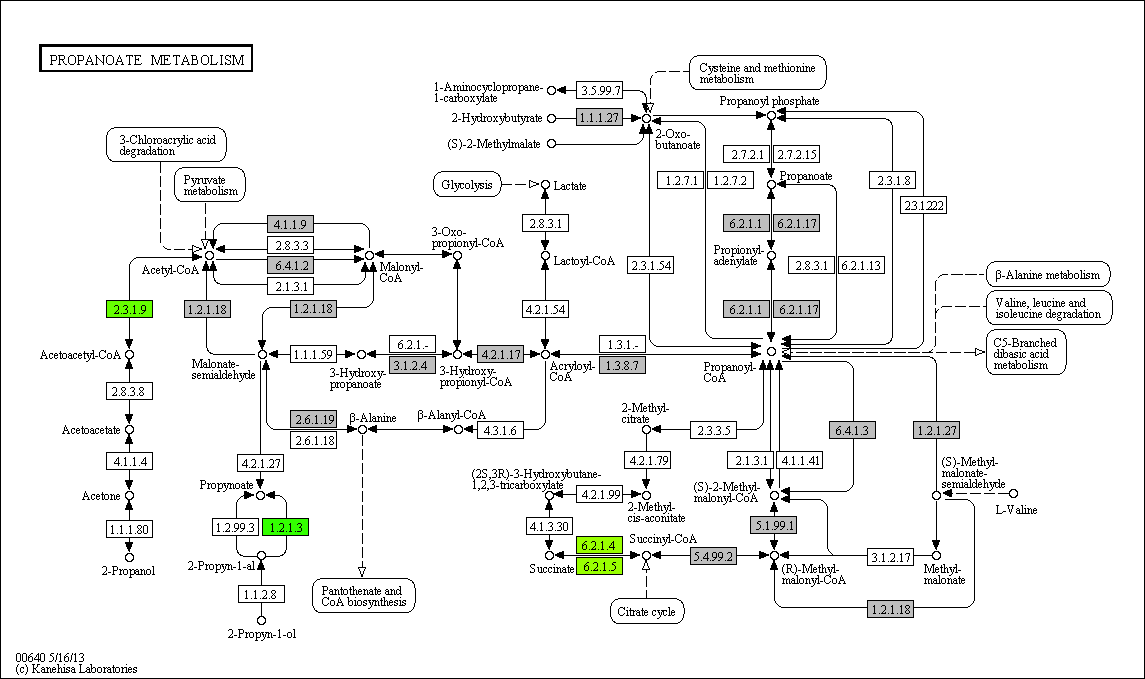

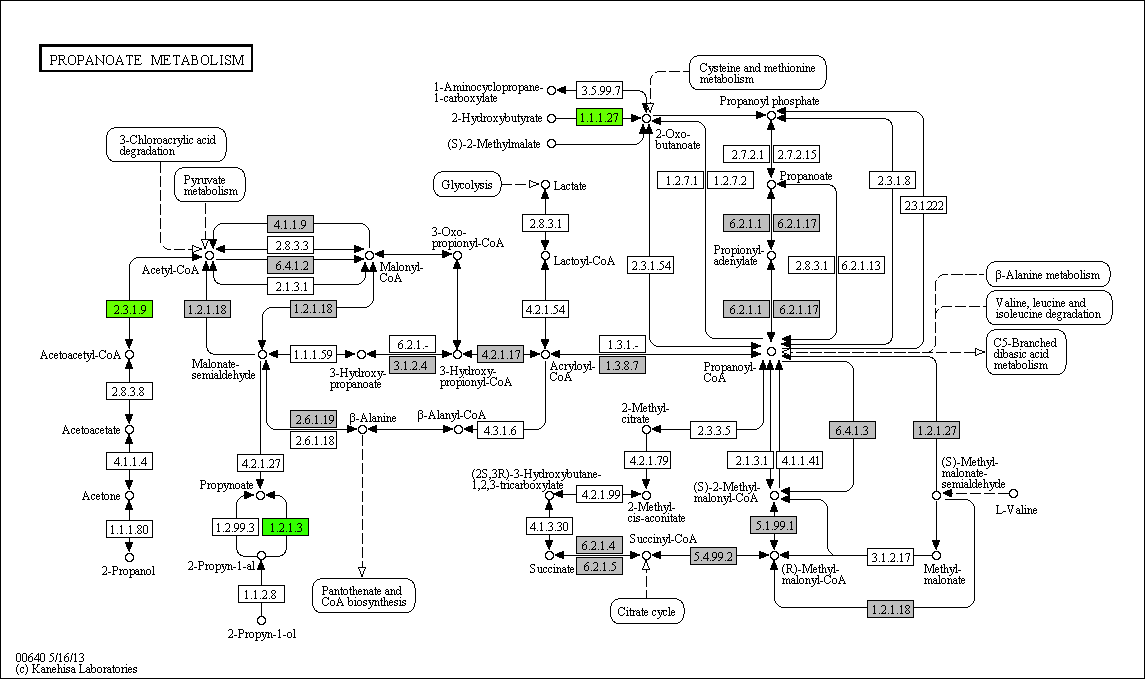


## 1.2 Energy Metabolism

### Oxidative Phosphorylation

-14 DAYS


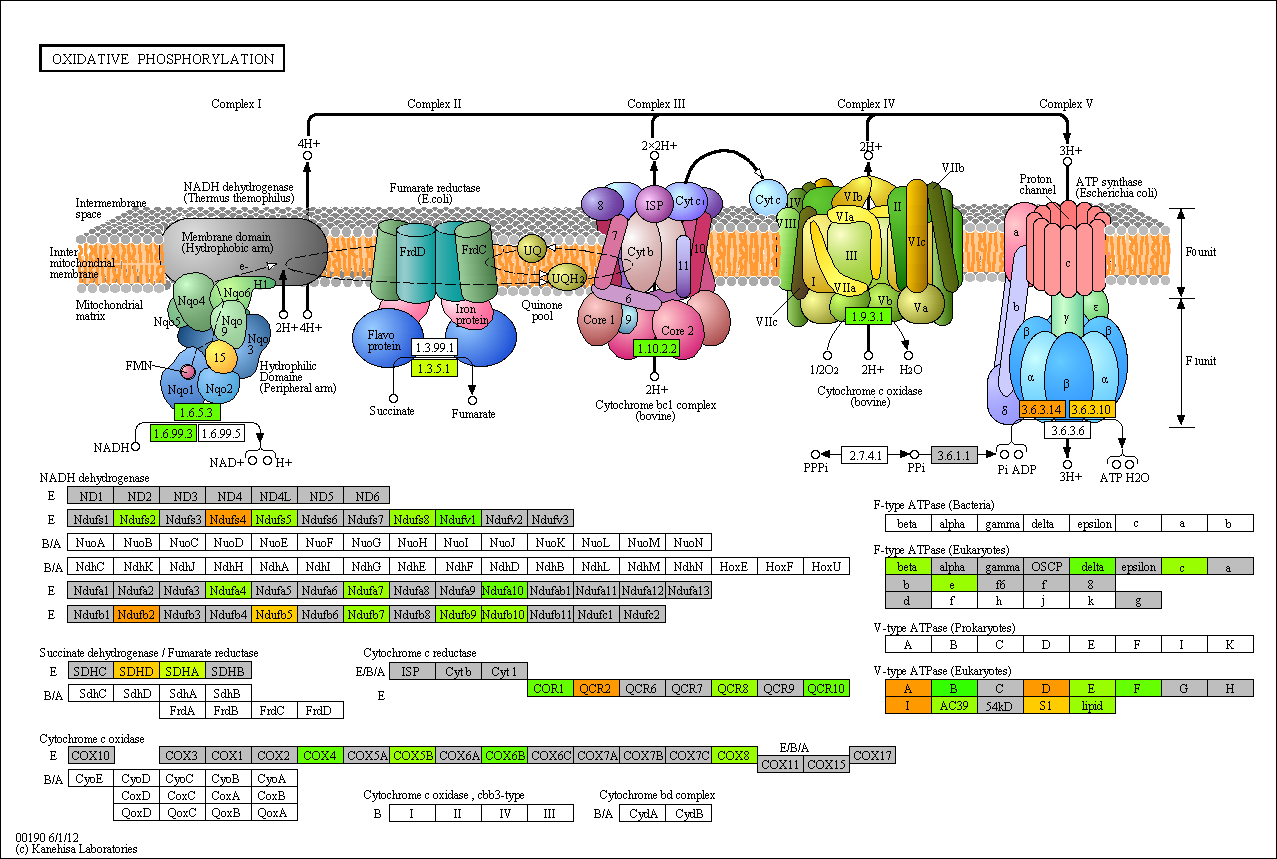


1 DAY 14 DAYS


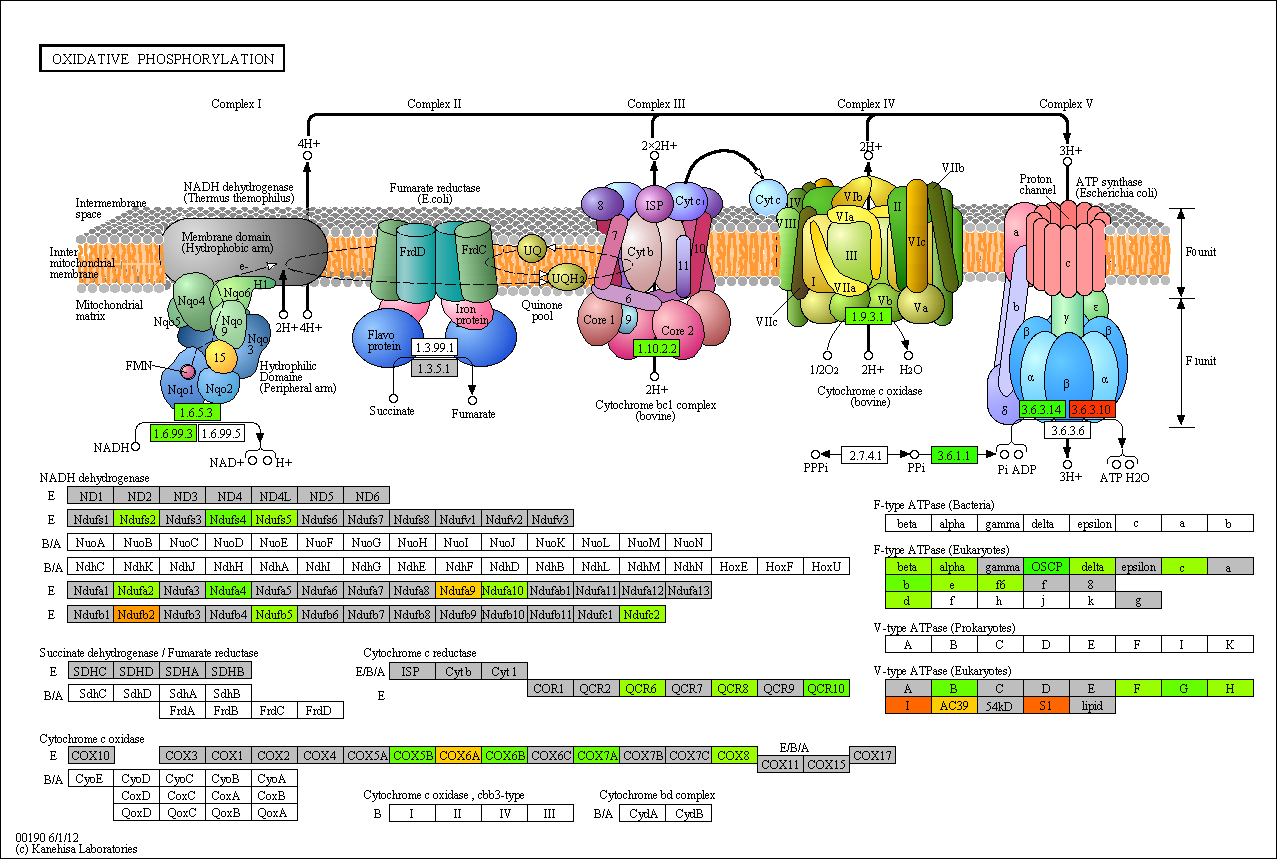

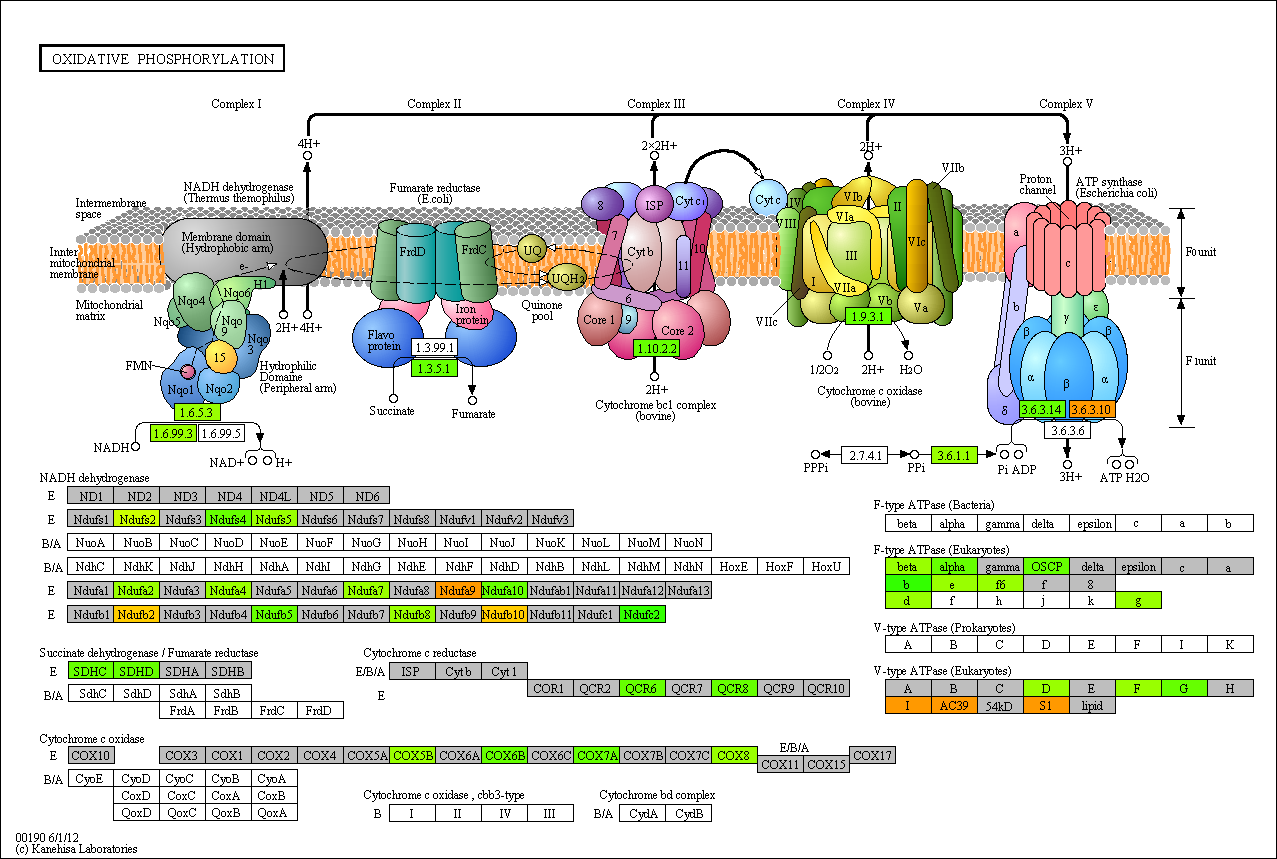


## 1.3 Lipid Metabolism

### Glycerolipid Metabolism

-14 DAYS


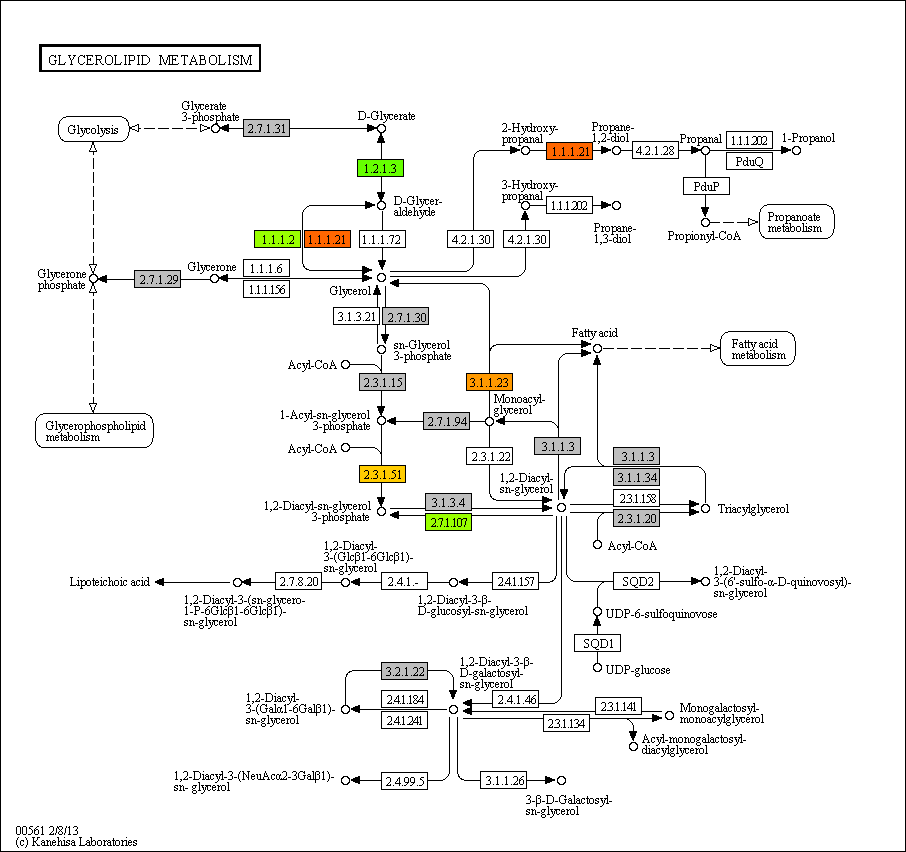


1 DAY 14 DAYS


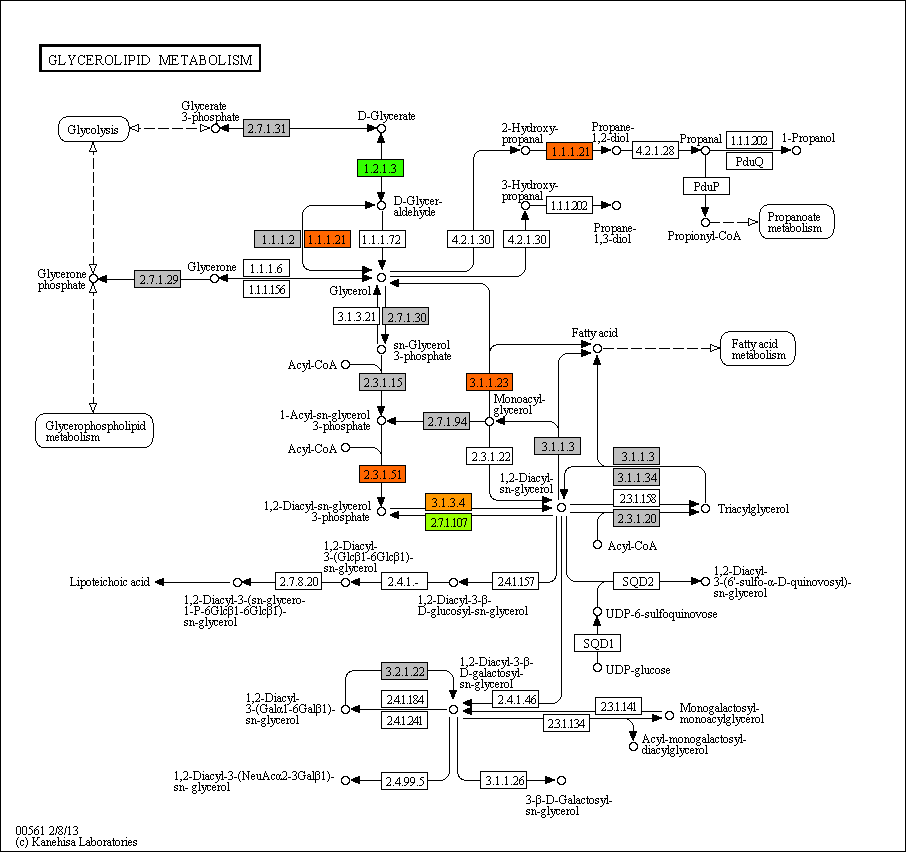

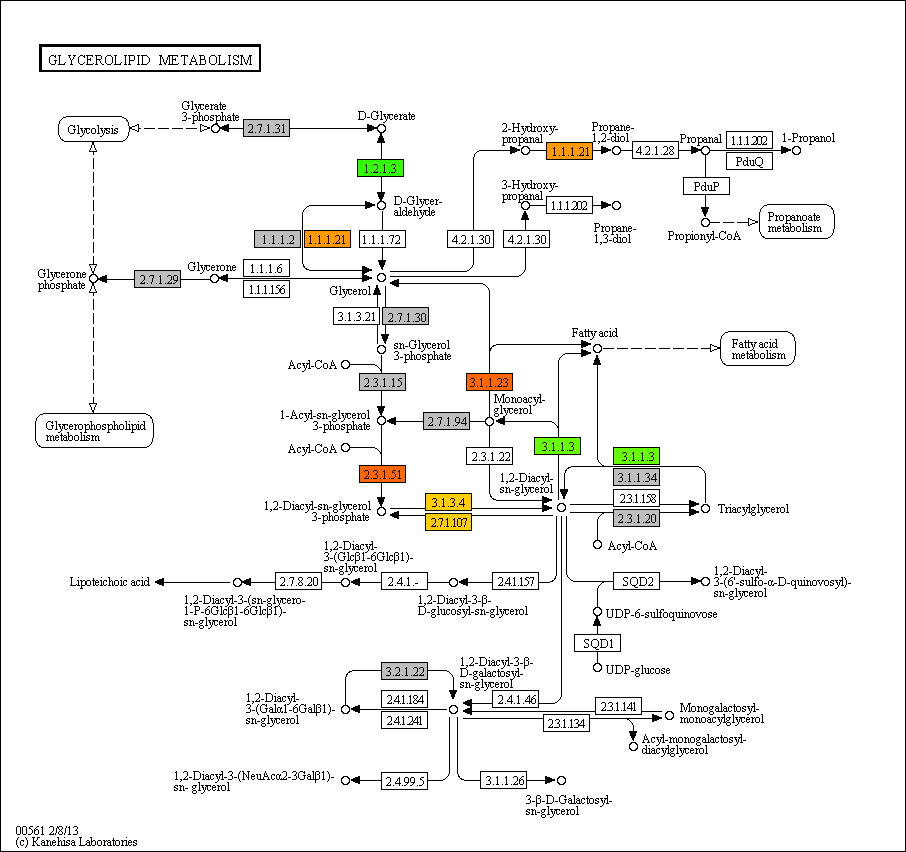


### Arachidonic Acid Metabolism

-14 DAYS


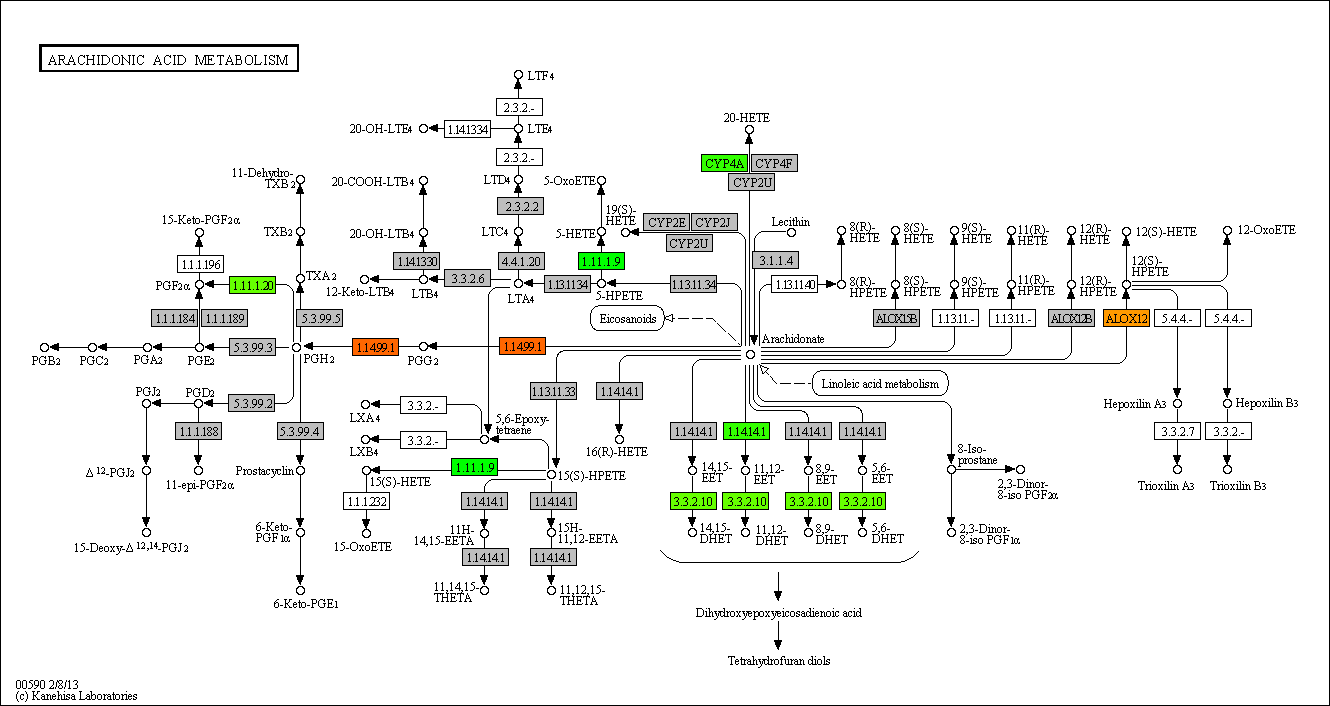


1 DAY 14 DAYS


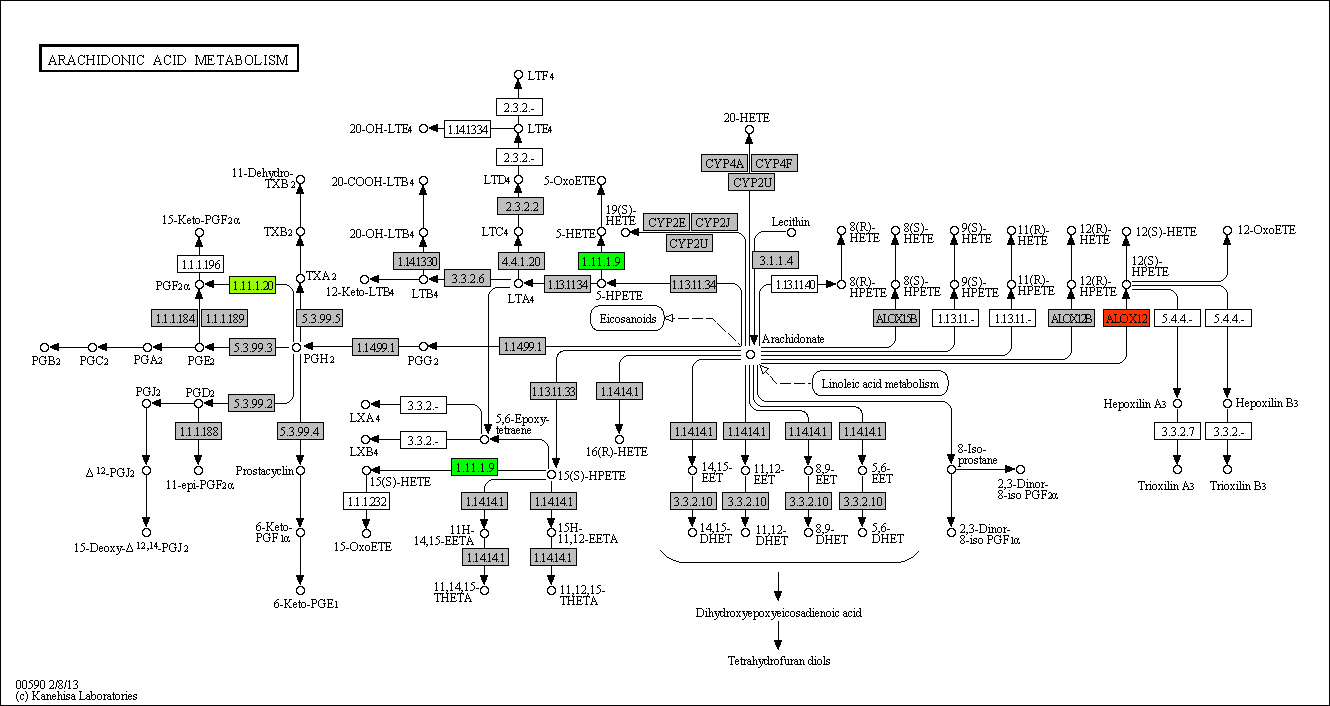

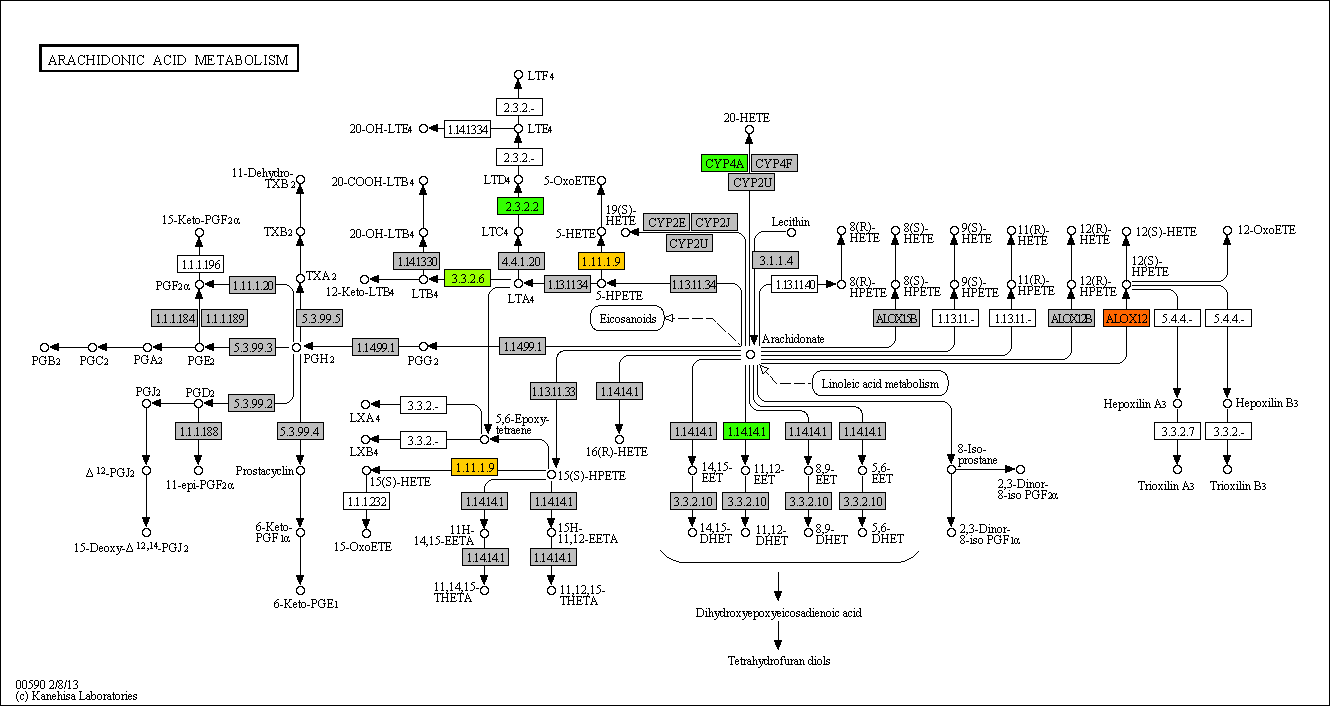


### Synthesis and Degradation of Ketone Bodies

1 DAY 14 DAYS


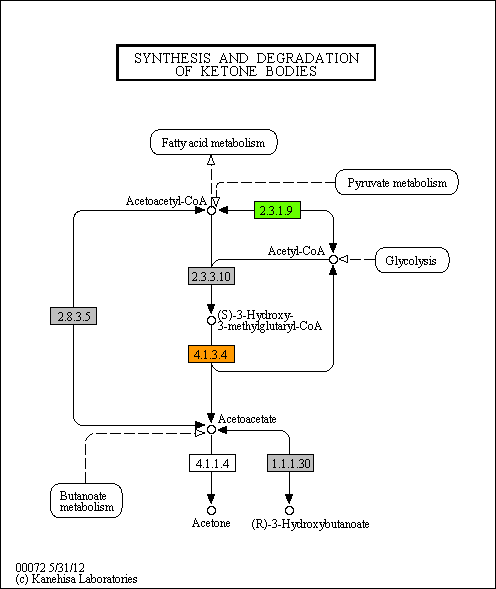

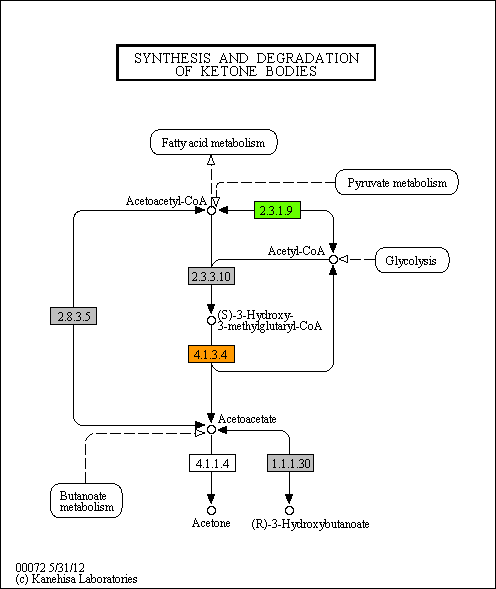


### Fatty acid Metabolism

-14 DAYS


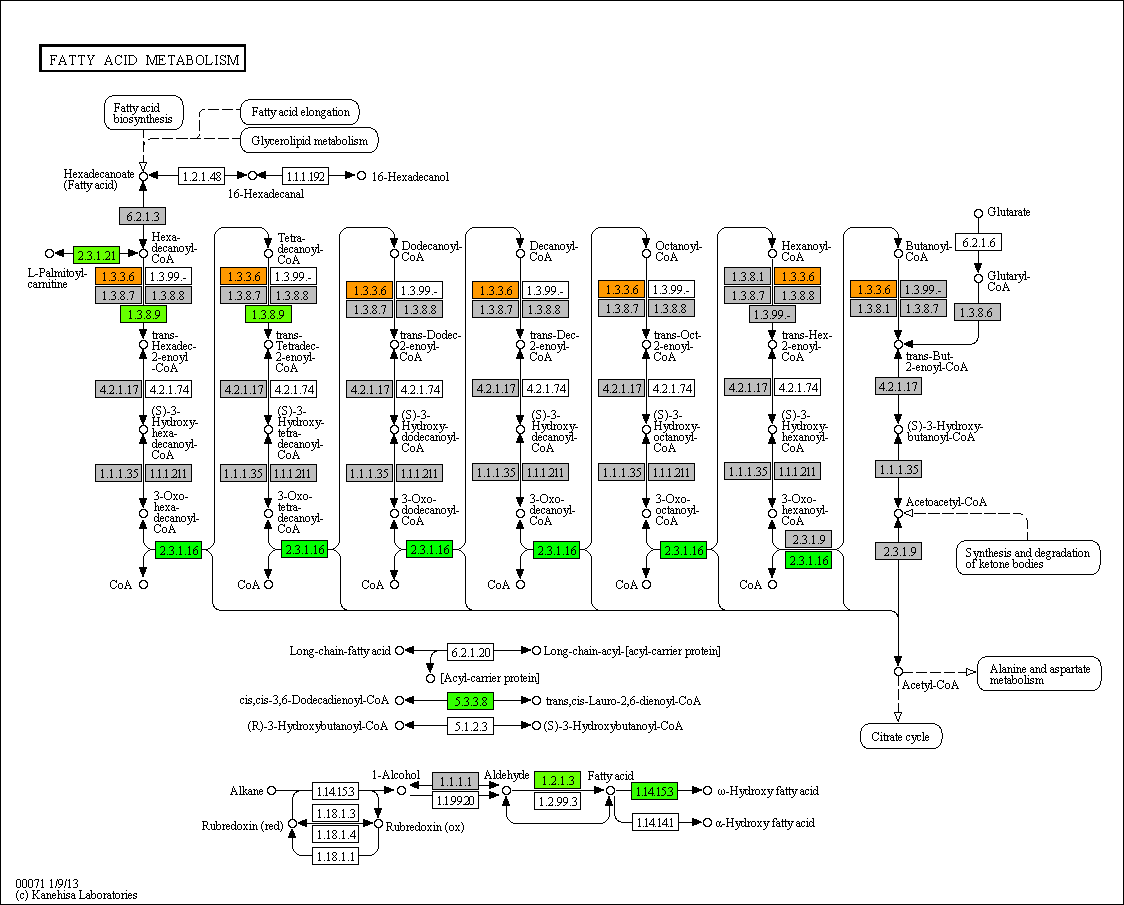


1 DAY 14 DAYS


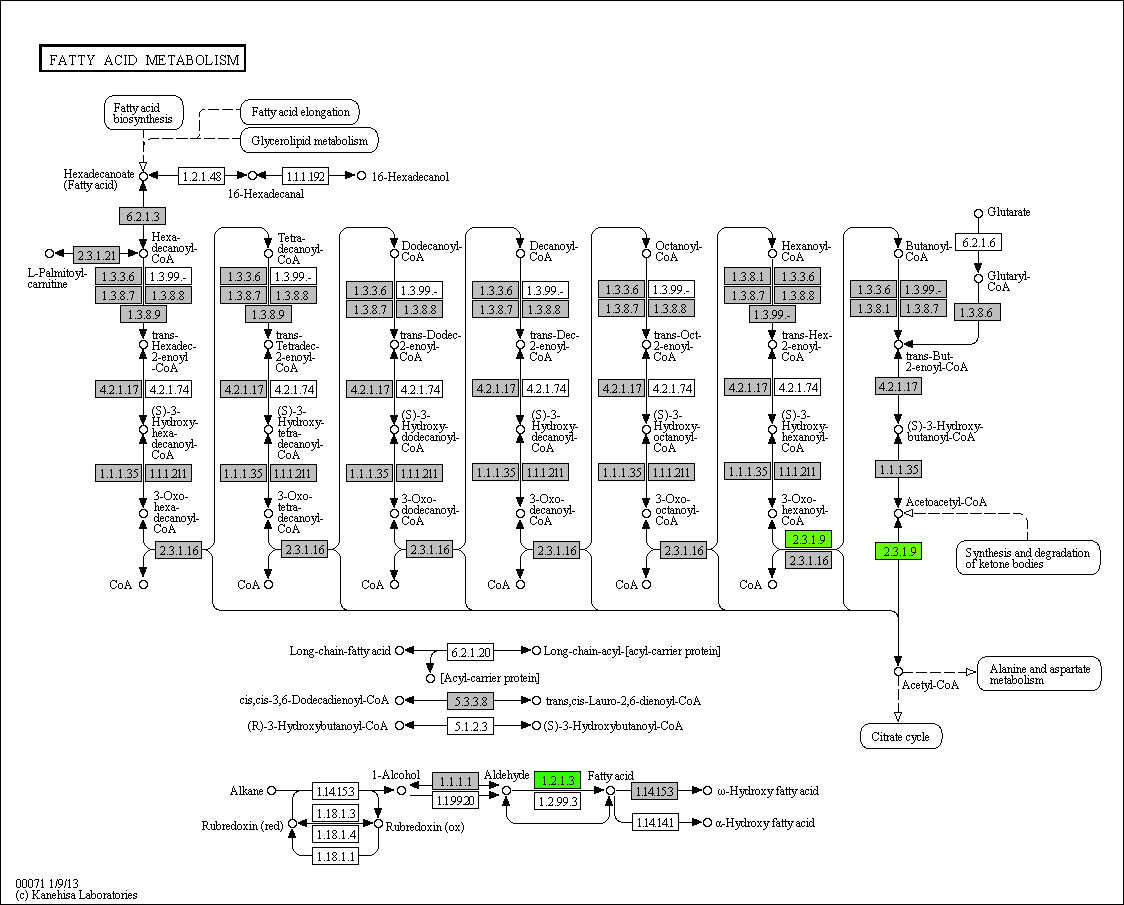

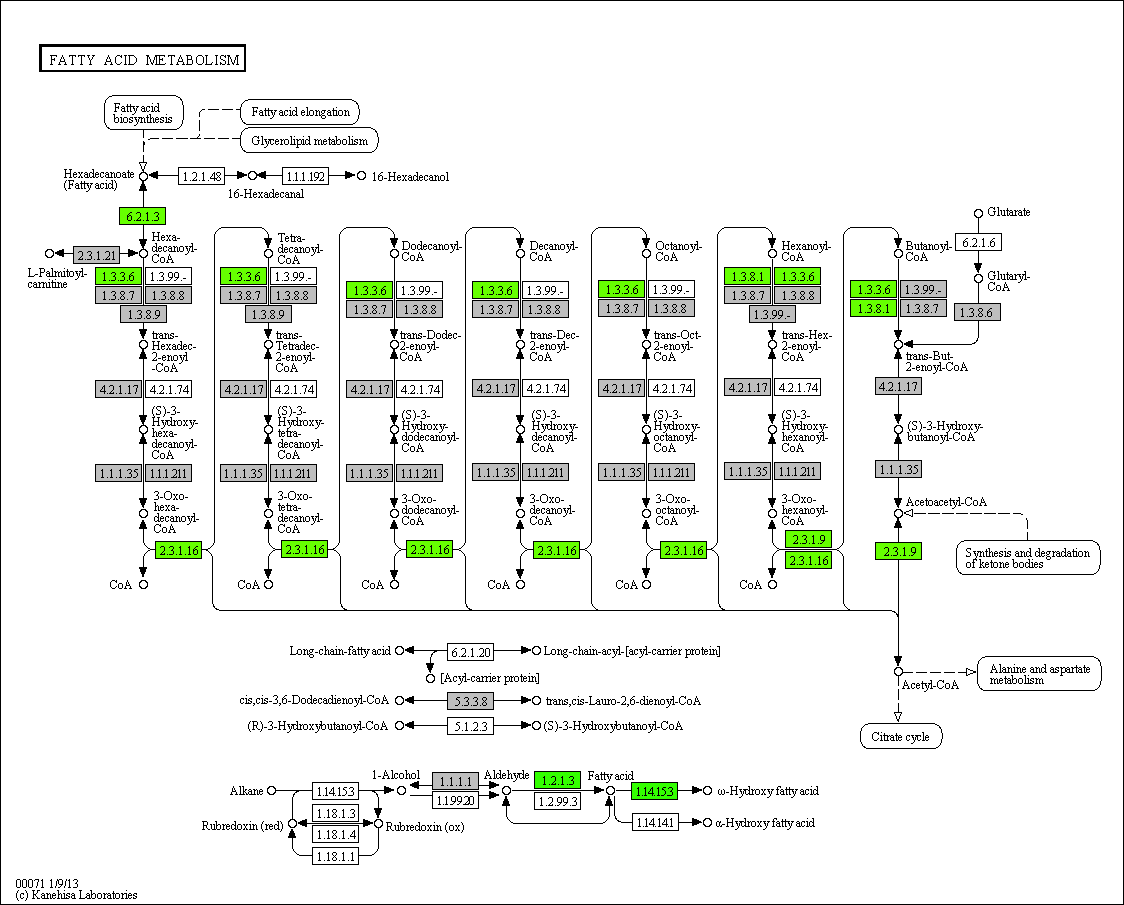


## 1.5 Amino Acid Metabolism

### Alanine, Aspartate and Glutamate metabolism

-14 DAYS


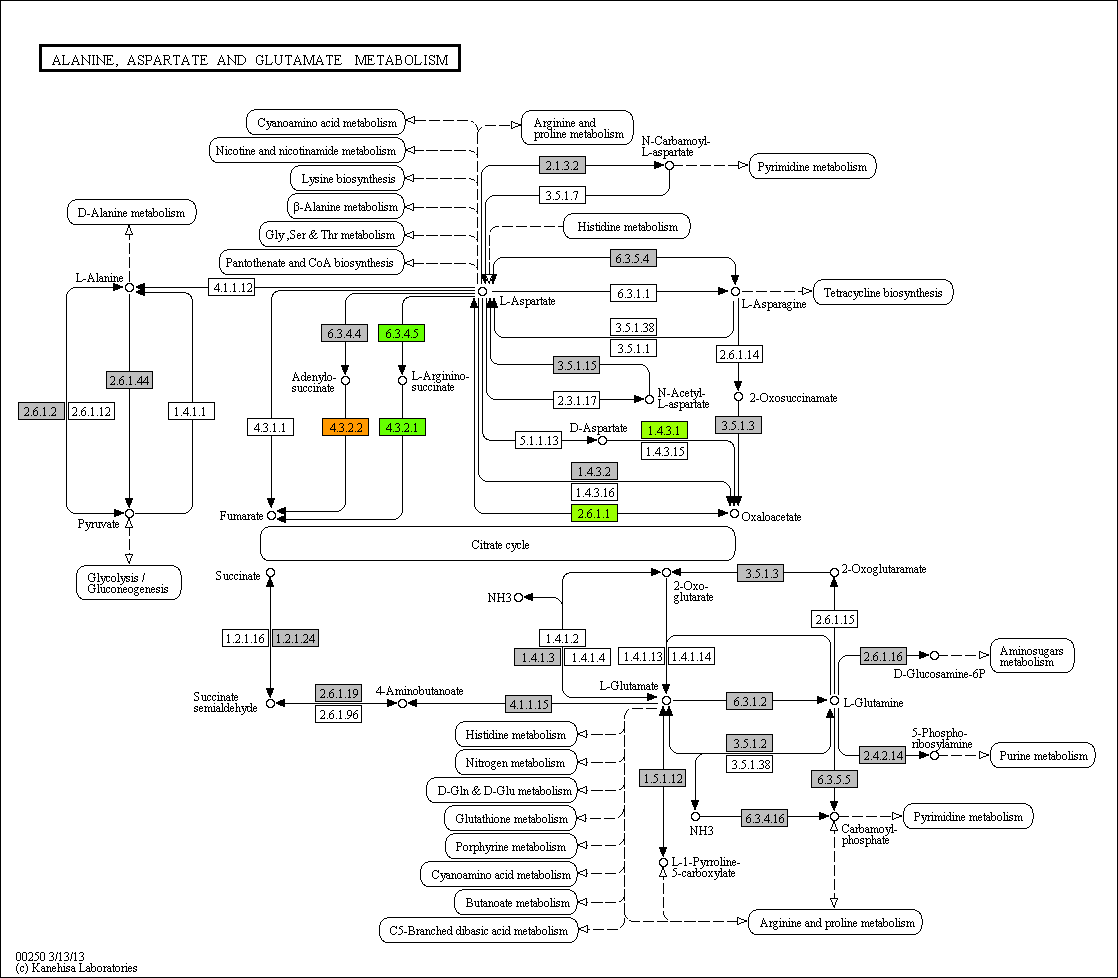


1 DAY 14 DAYS


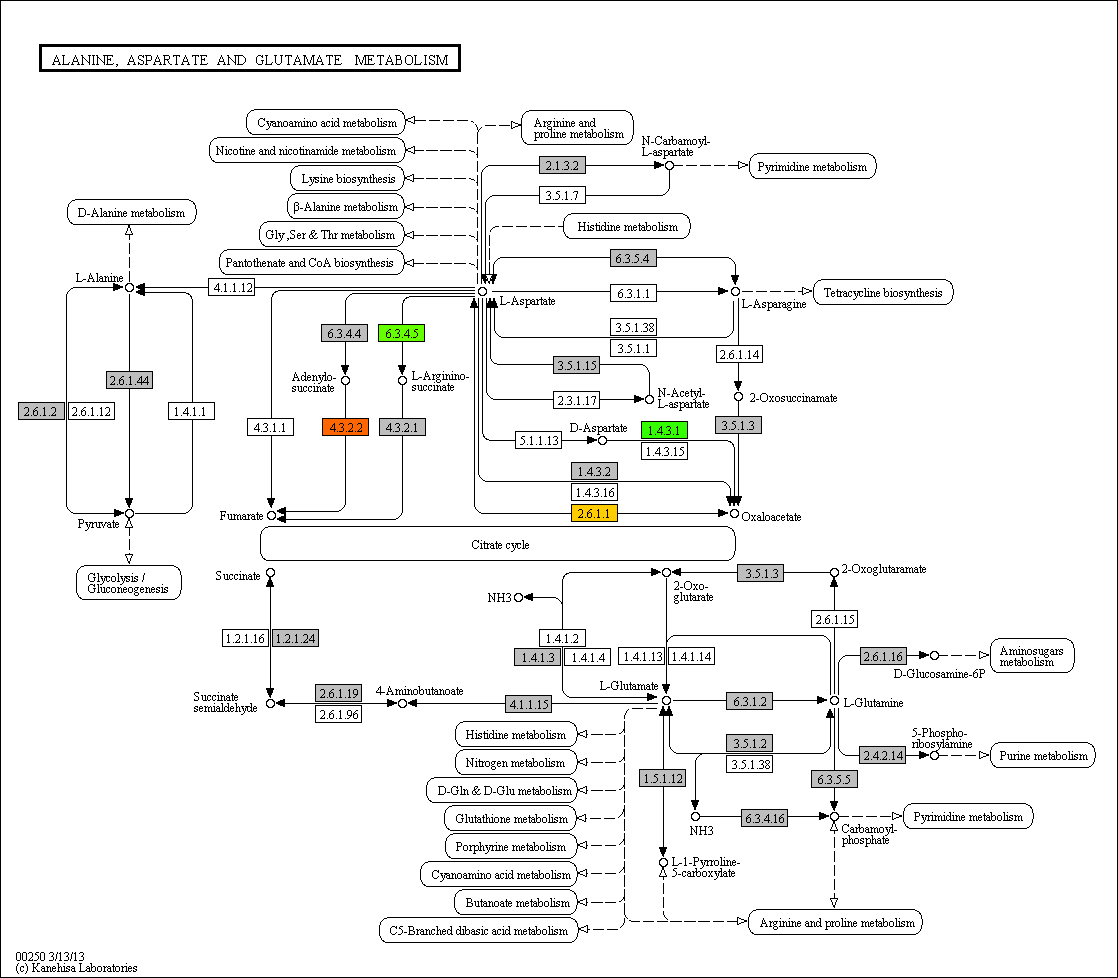

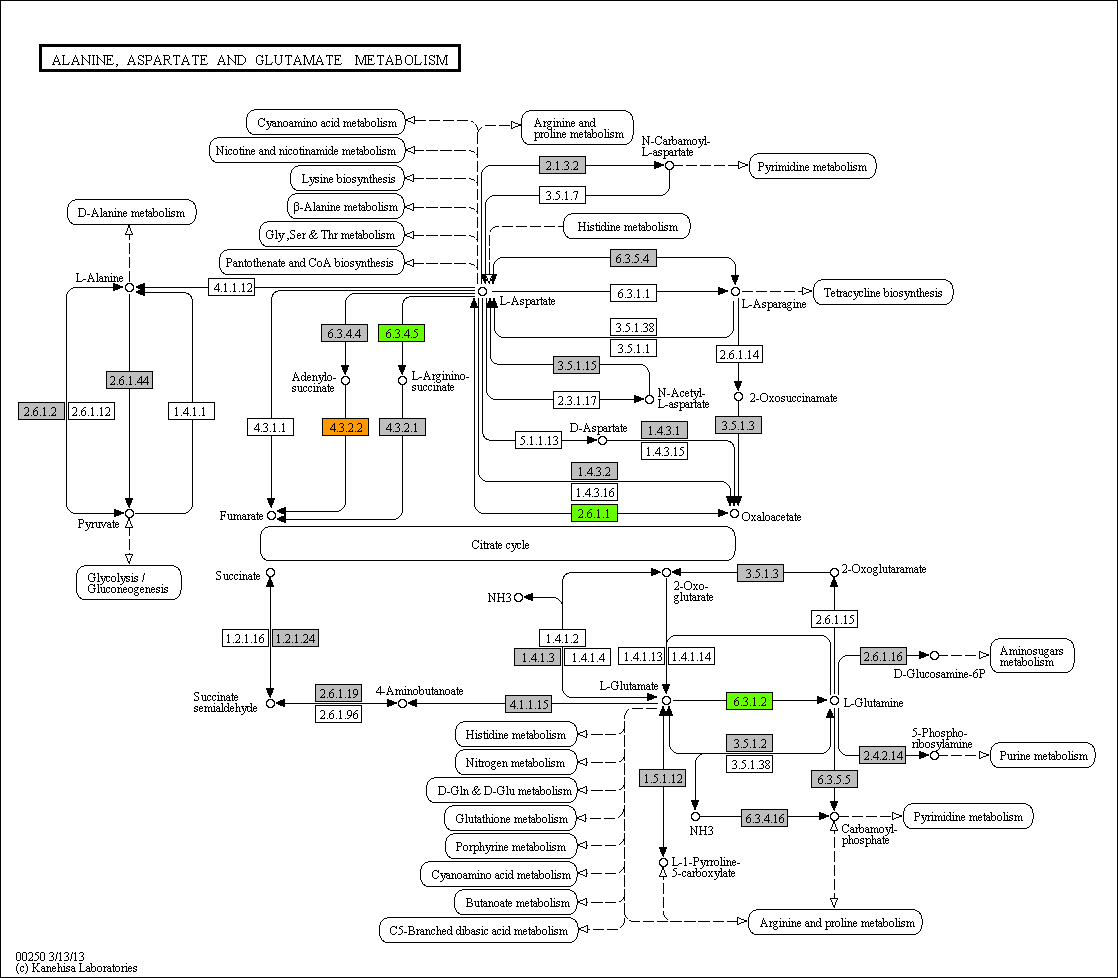


### Valine, leucine and isoleucine degradation

-14 DAYS


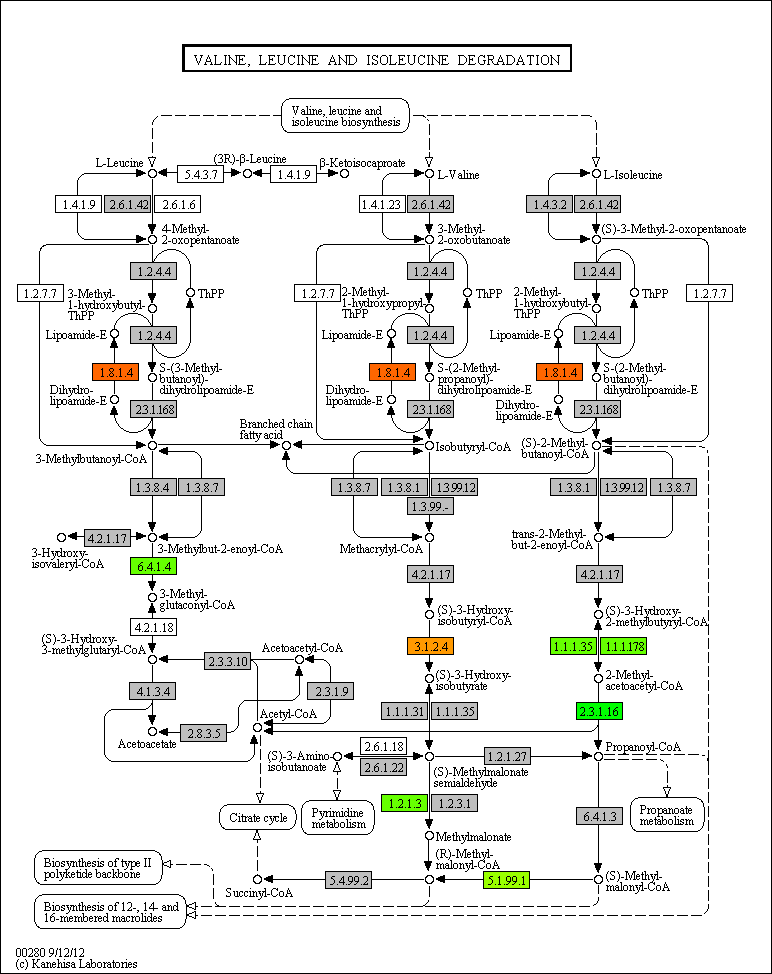
1 DAY
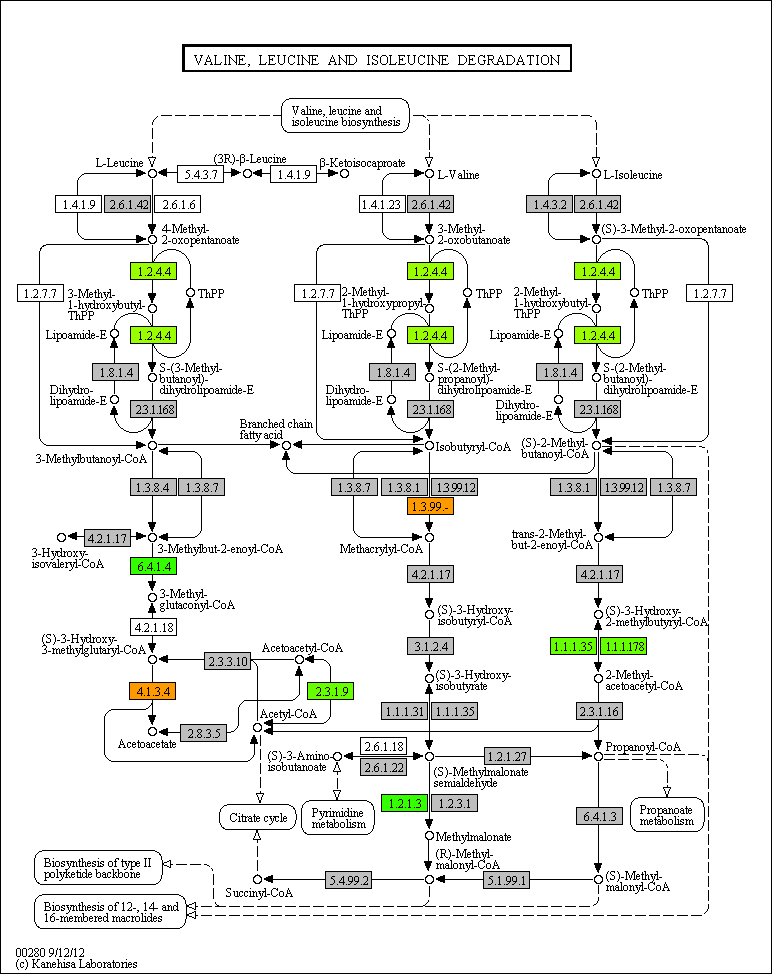


14 DAYS


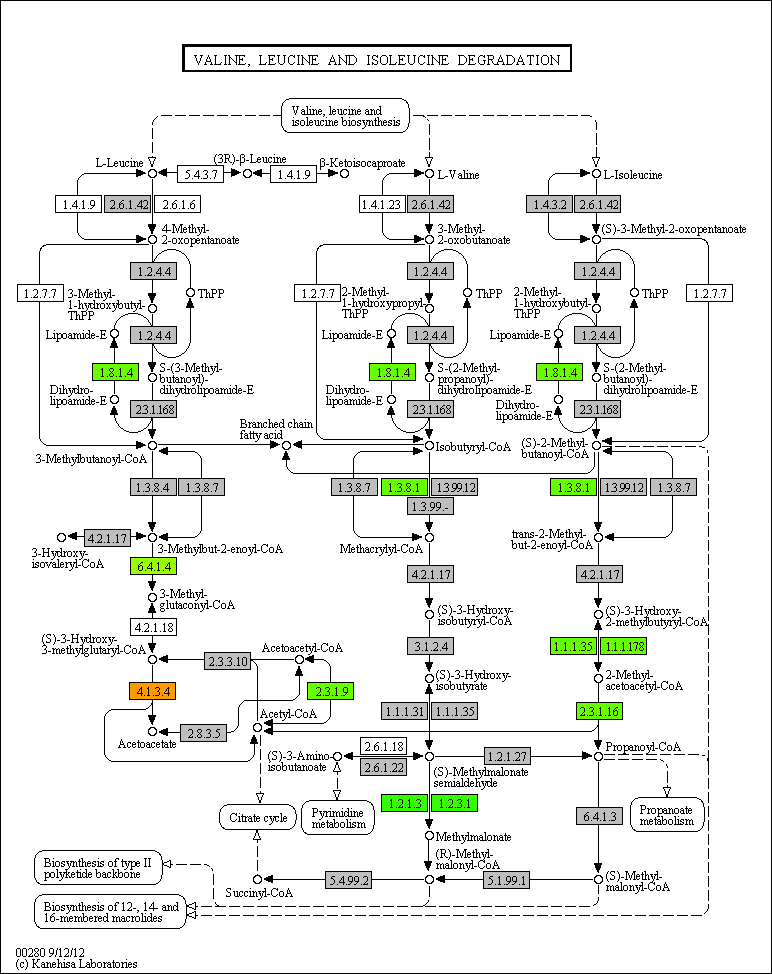


**Glutathione metabolism**

-14 DAYS


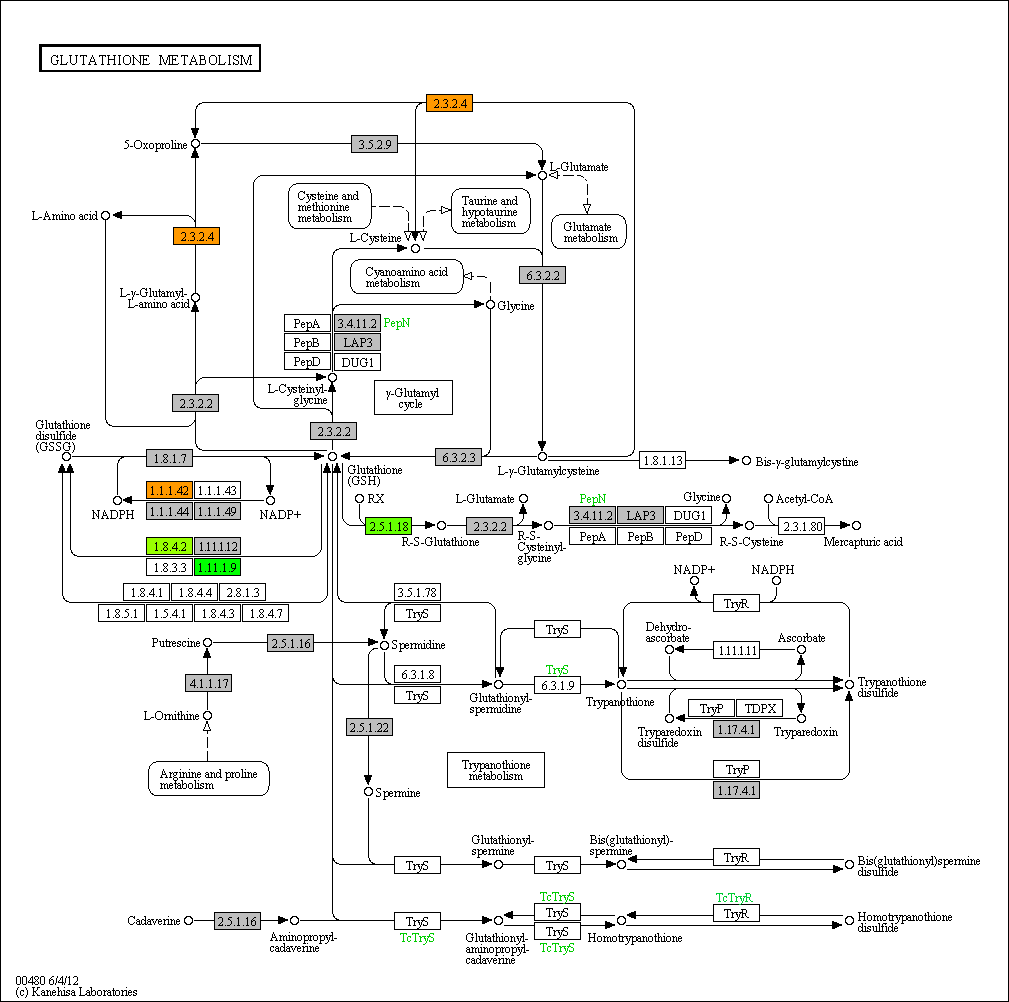


1 DAY 14 DAYS


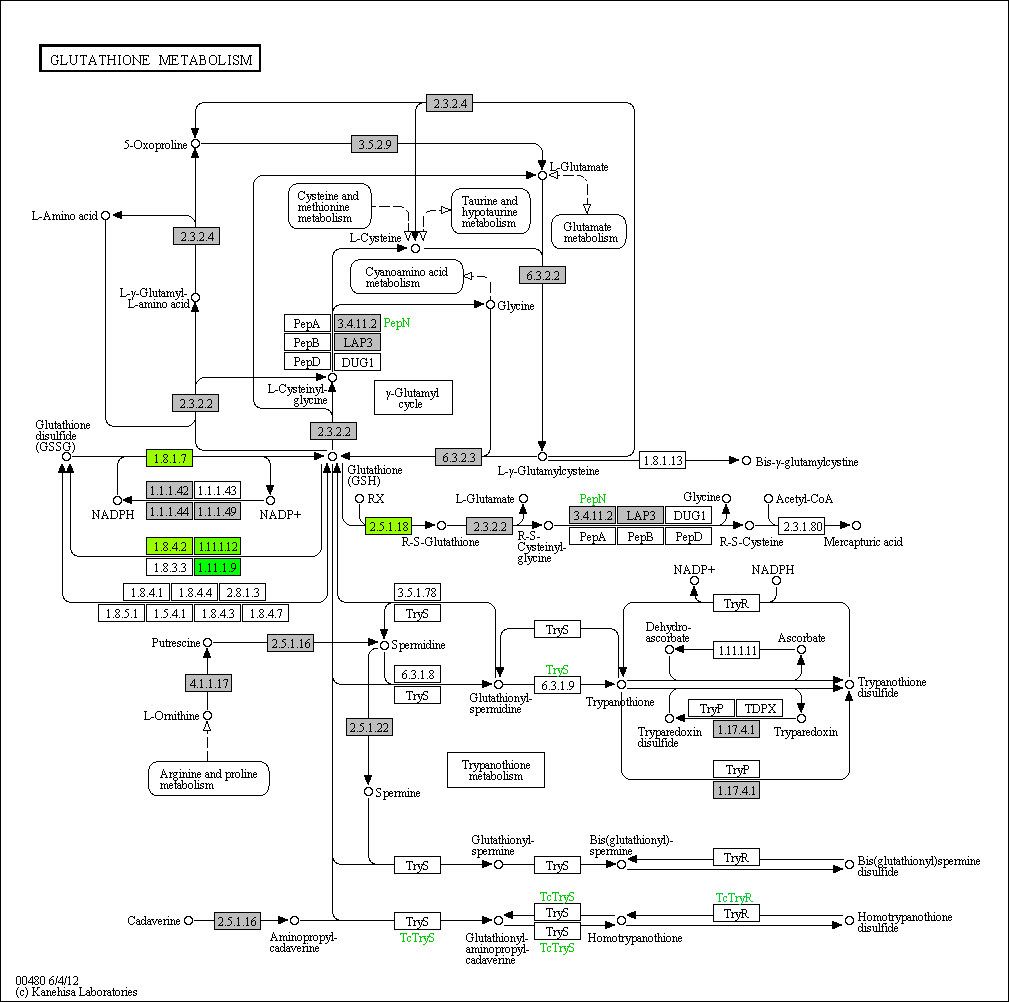

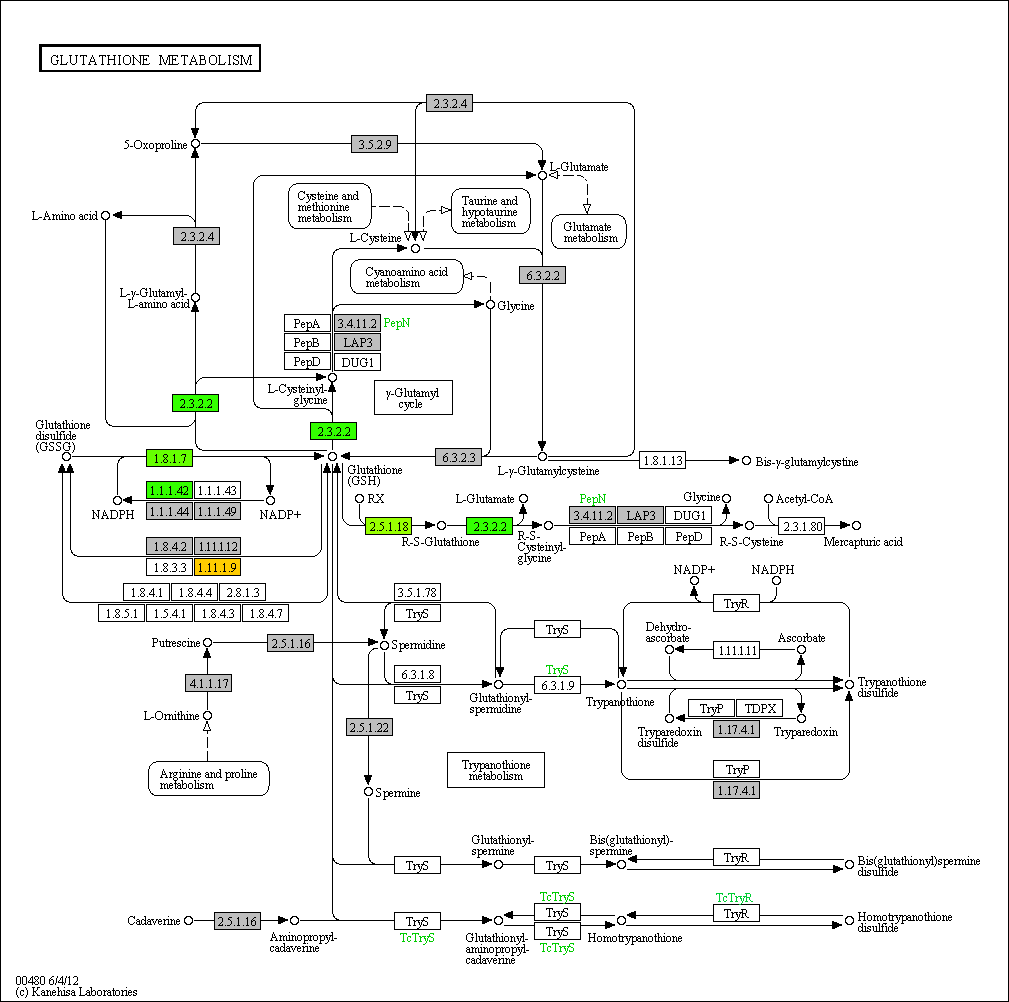


## 1.7 Glycan biosynthesis and metabolism

### N-Glycan Biosynthesis

-14 DAYS 1 DAY


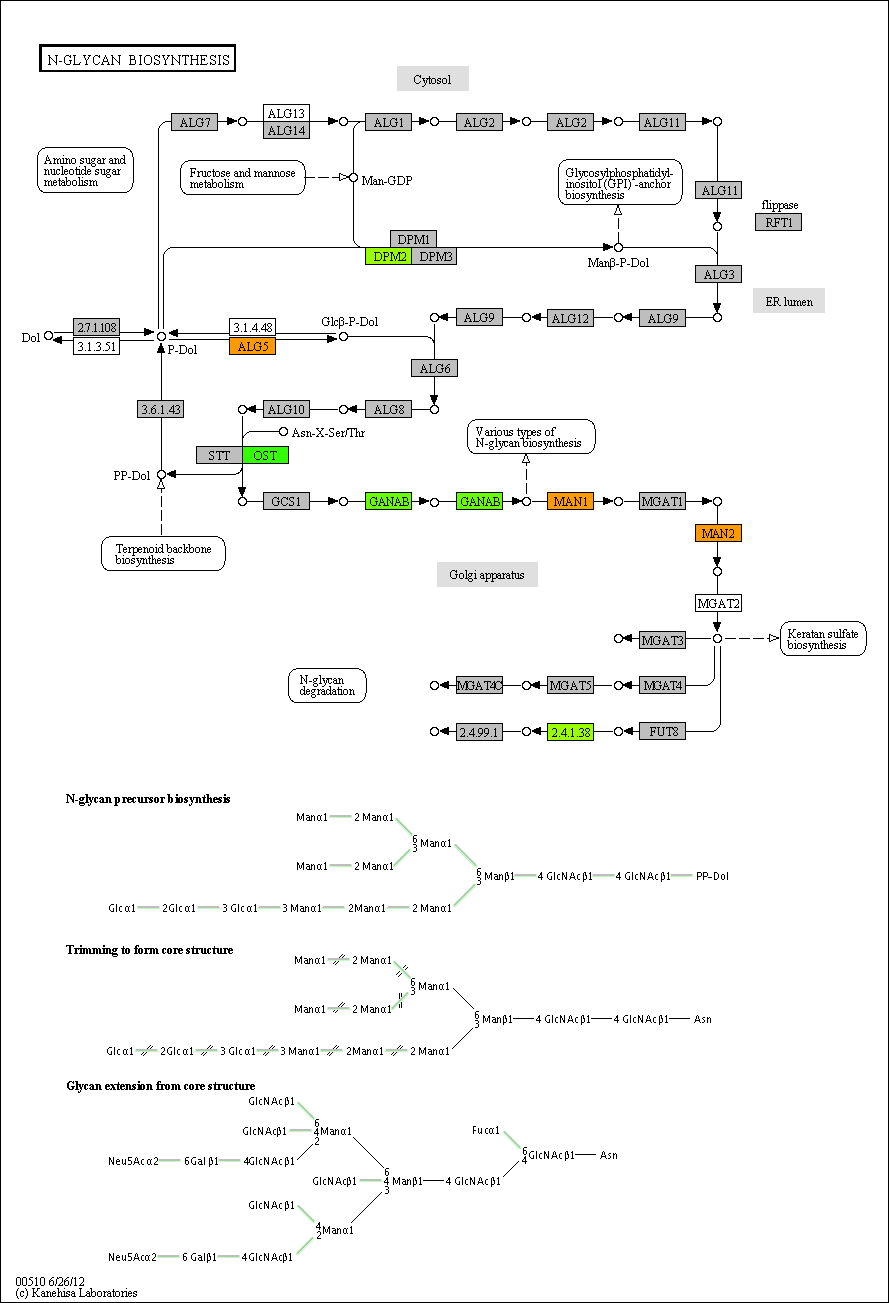

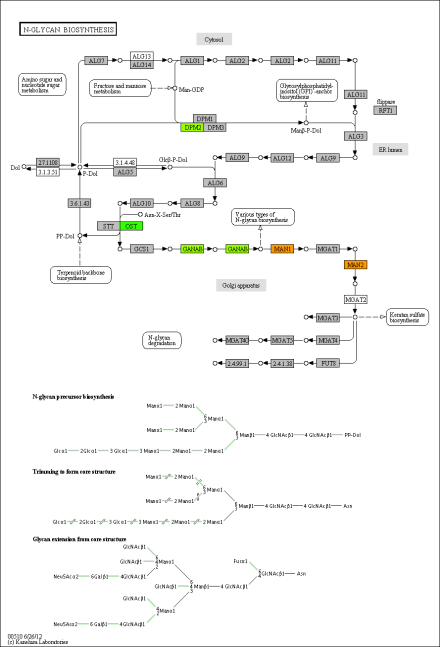


14 DAYS

## 1.8 Metabolism of Cofactors and Vitamins

### Ubiquinone (coenzyme Q) and other terpenoid-quinone biosynthesis

-14 DAYS


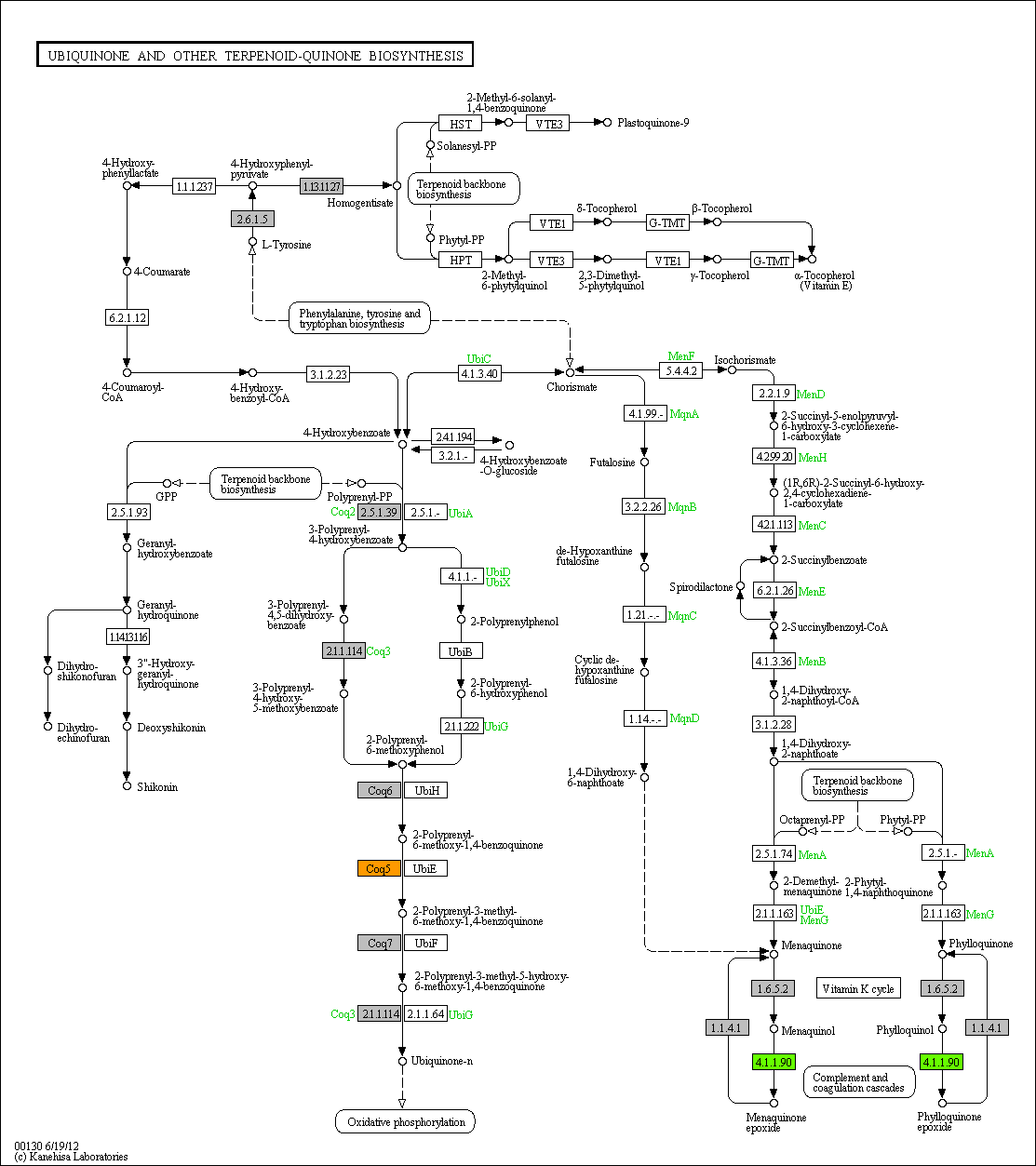


1 DAY 14 DAYS


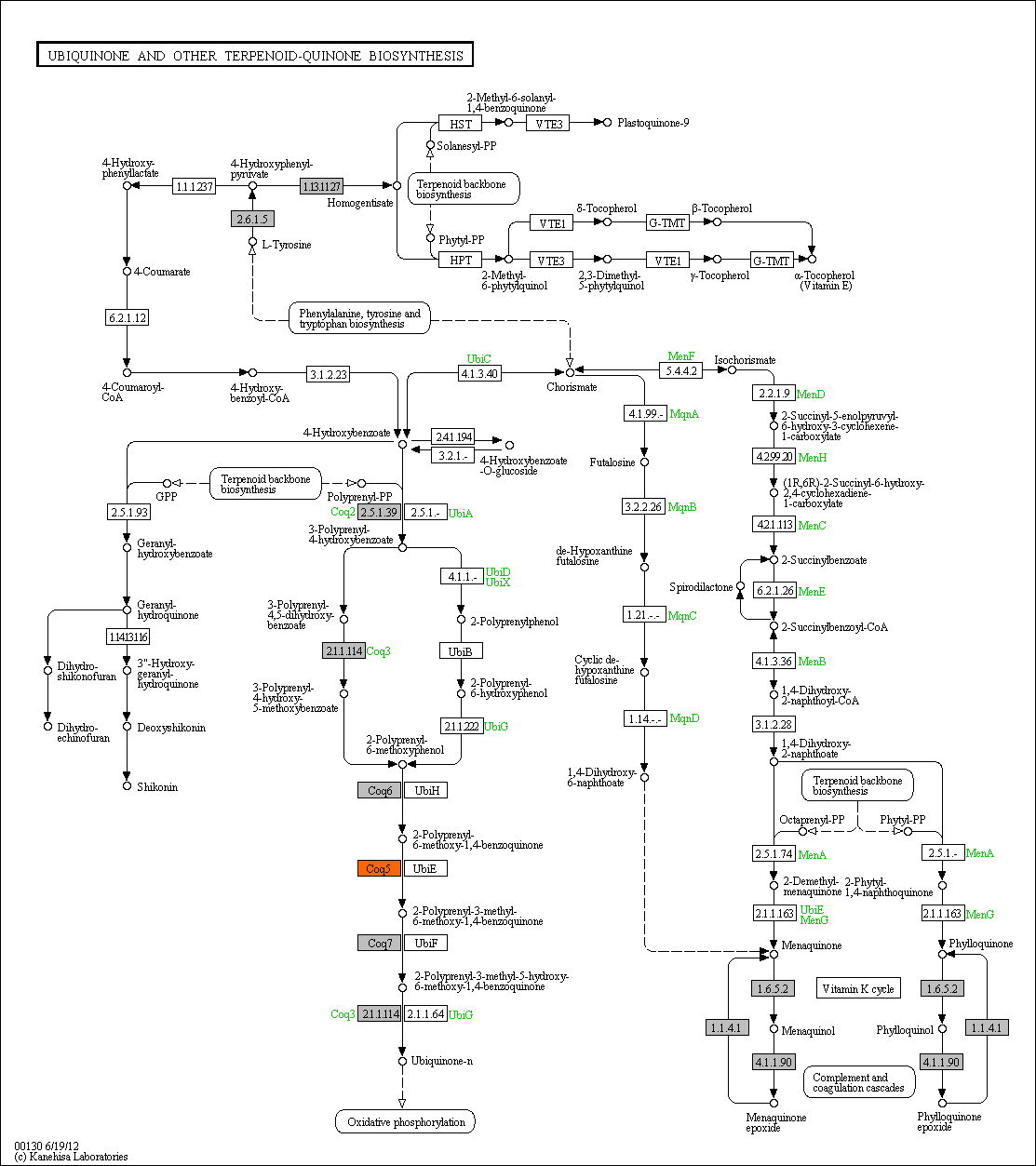

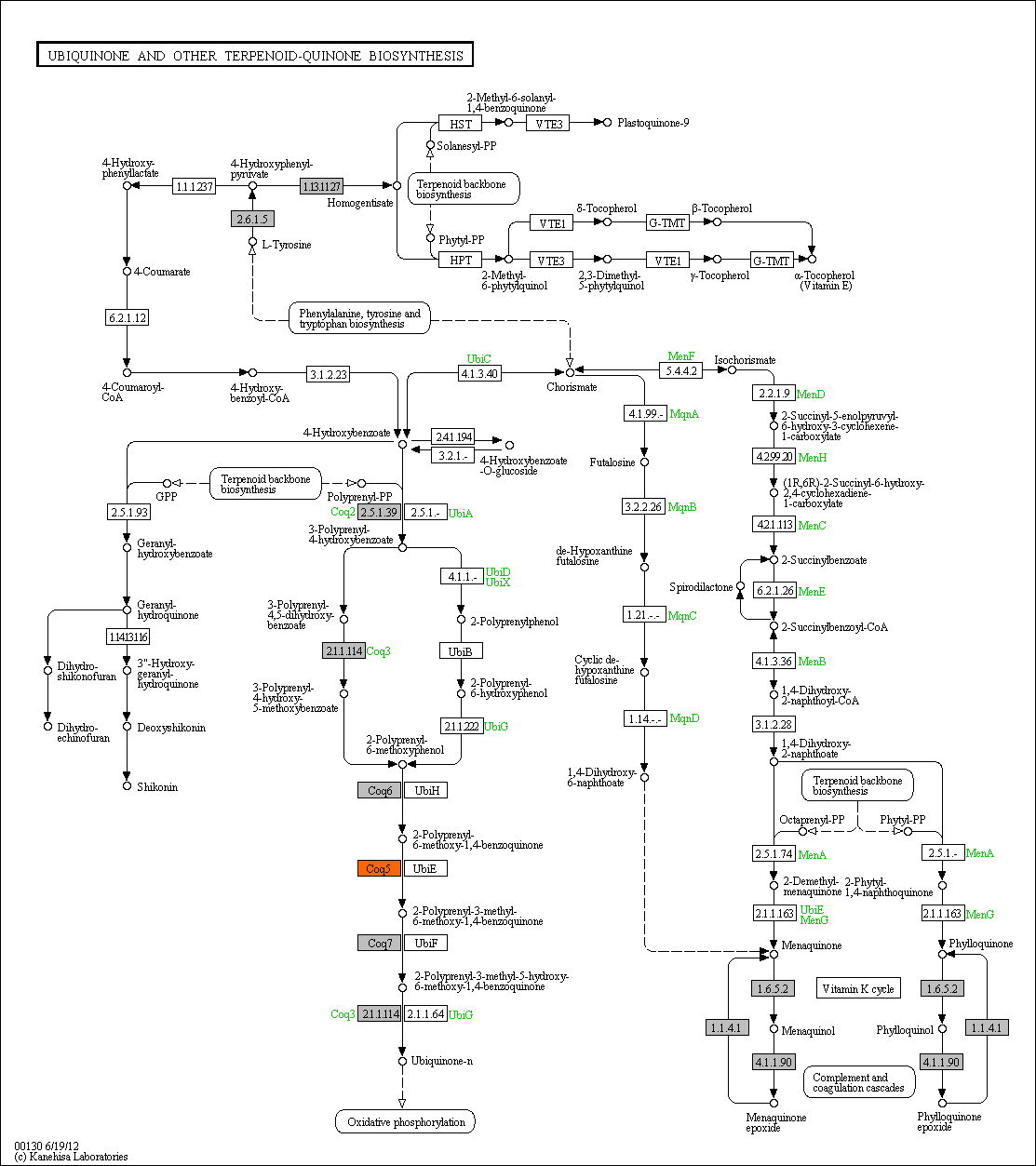


### Folate Biosynthesis

-14 DAYS


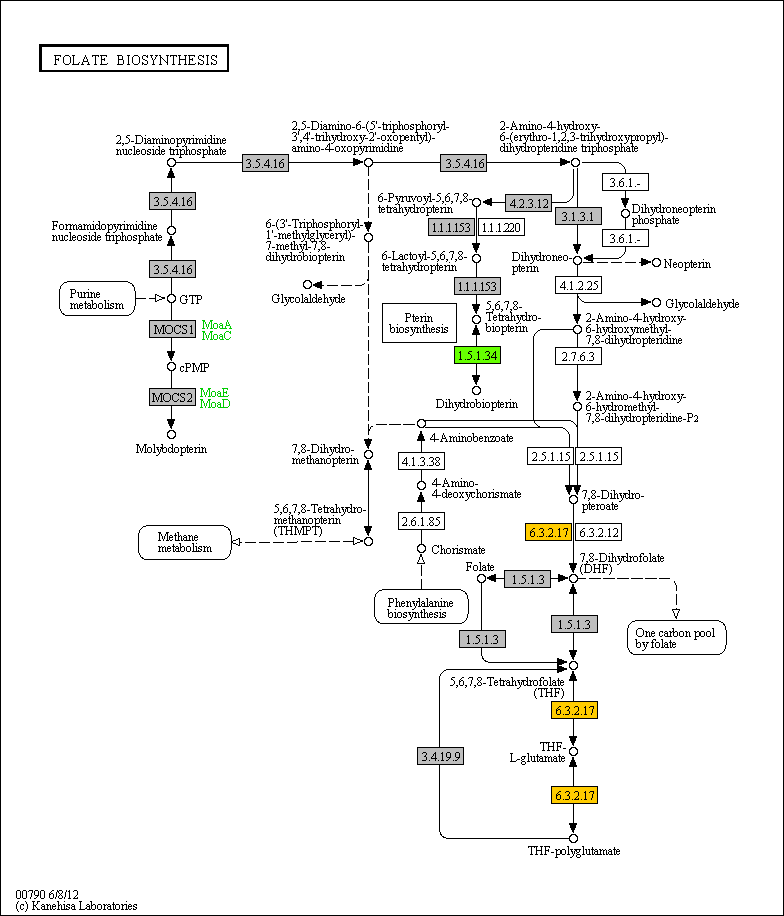


1 DAY 14 DAYS


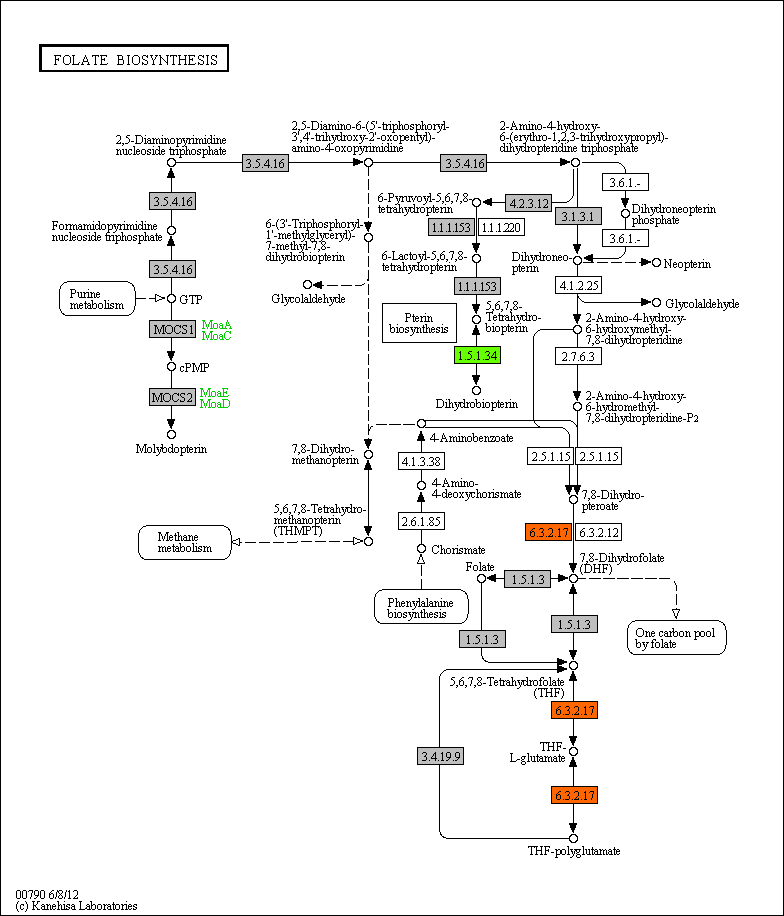

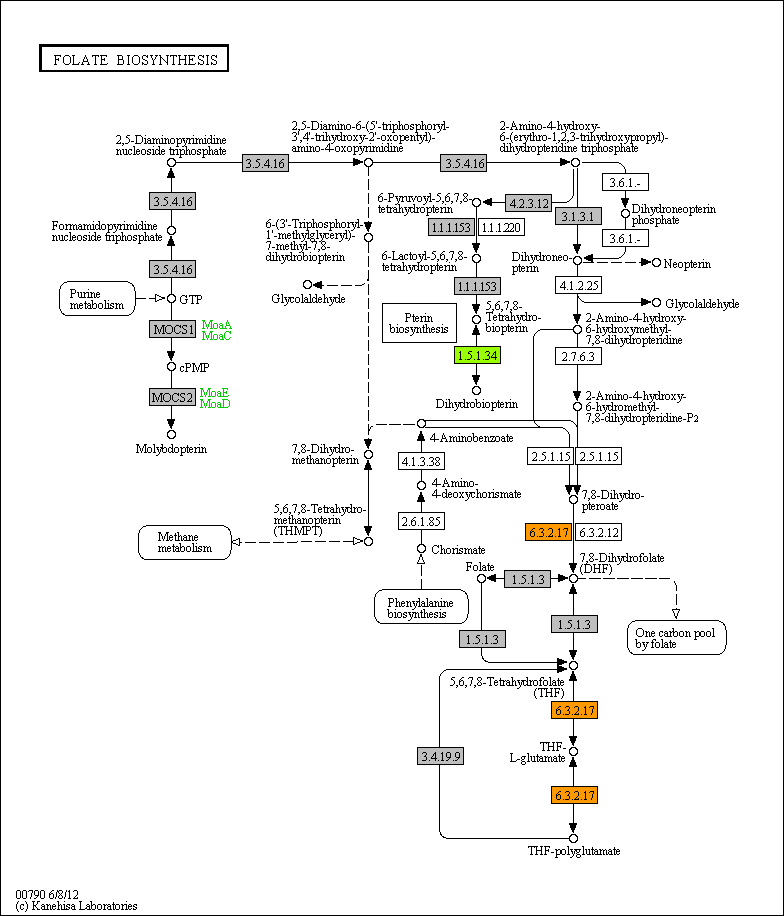


## 1.9 Metabolism of Terpenoids and Polyketides

### Terpenoid backbone biosynthesis

-14 DAYS


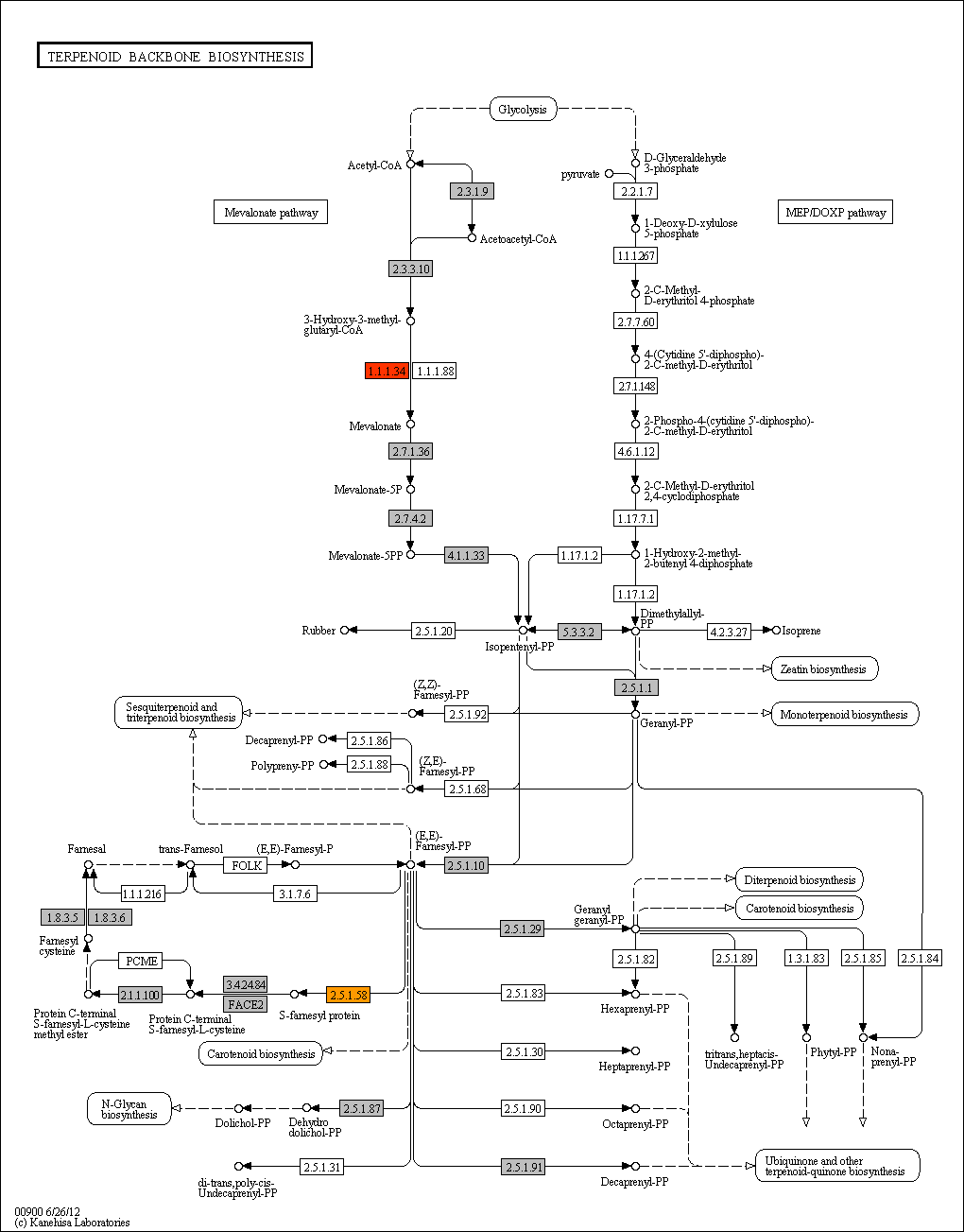


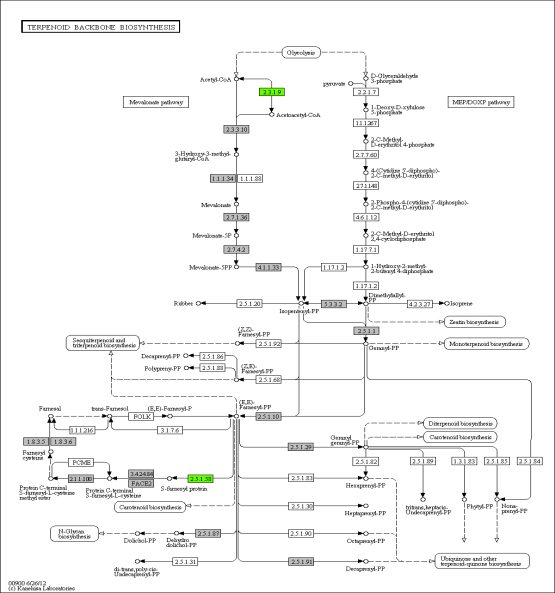

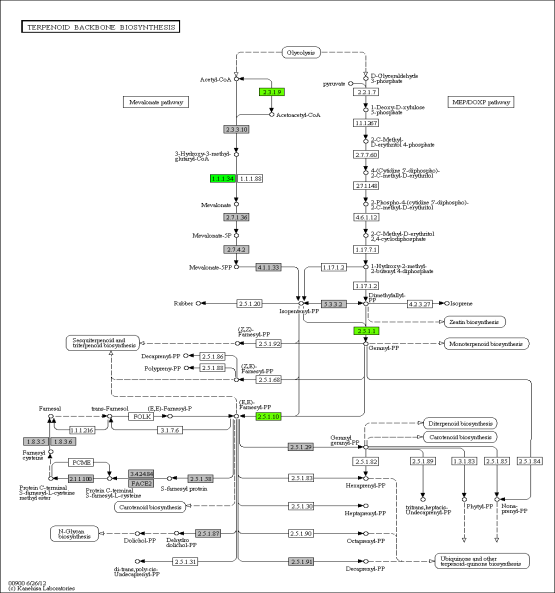
1 DAY 14 DAYS

## 1.10 Biosynthesis of other secondary metabolites

### Caffeine metabolism

1 DAY


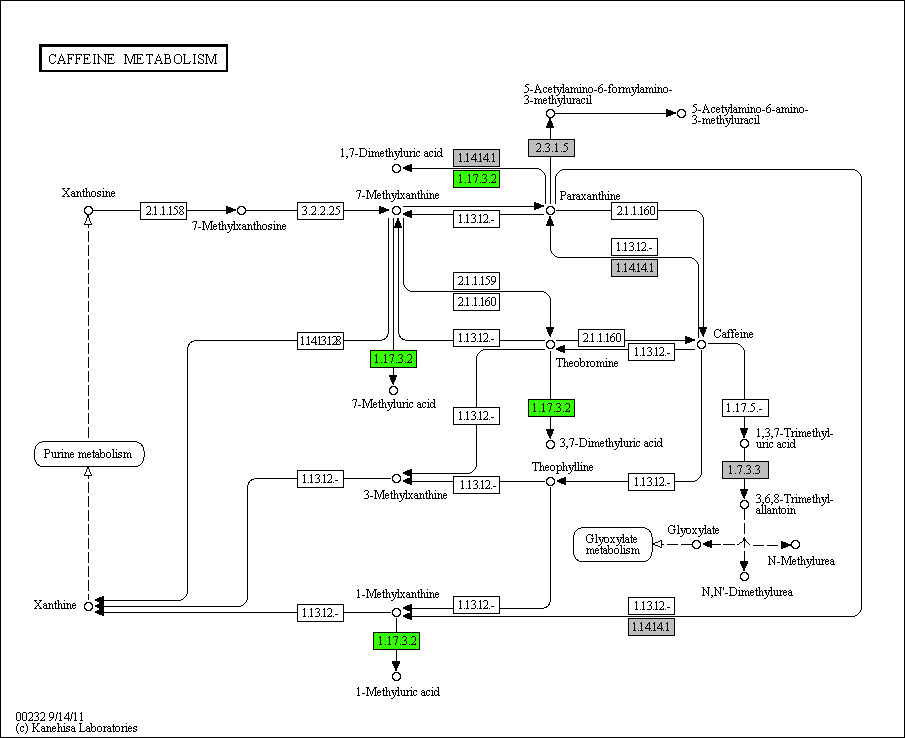


# 2. Genetic Information processing

## 2.1 Transcription

### Basal Transcription factors

-14 DAYS


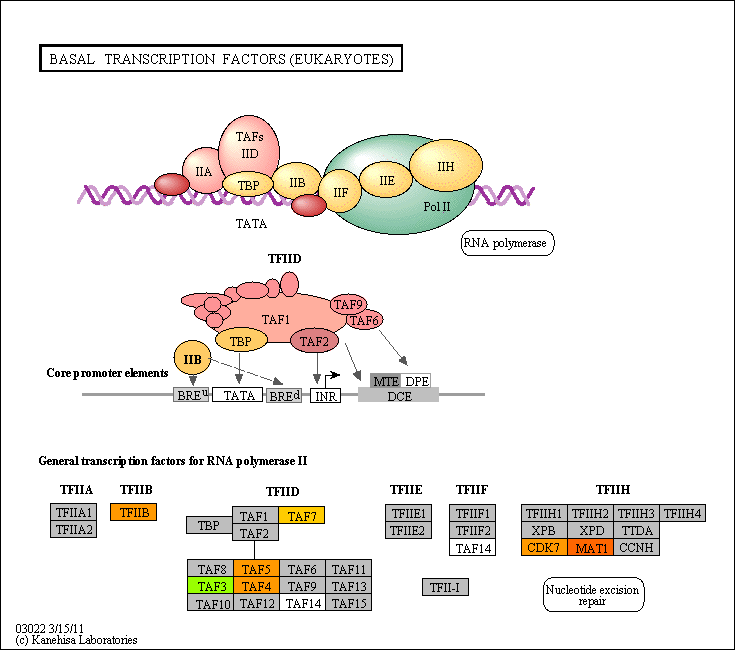


1 DAY 14 DAYS


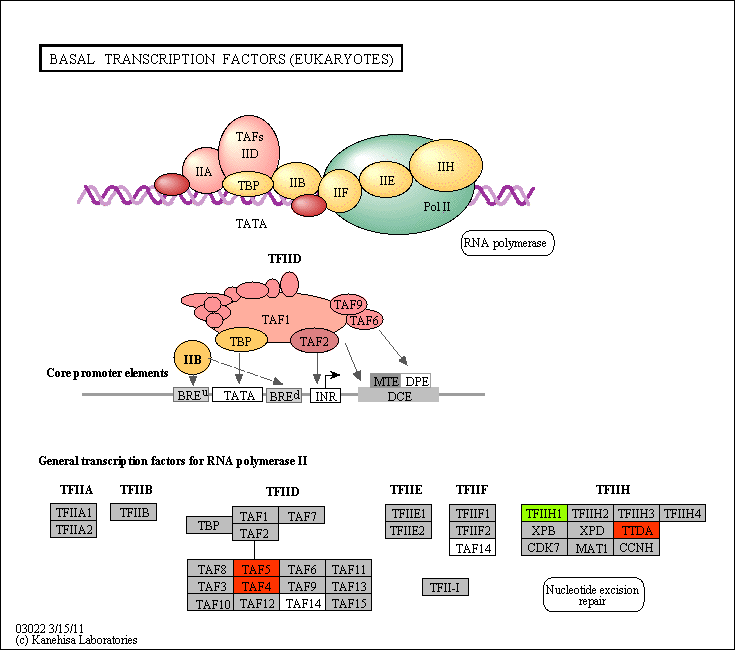

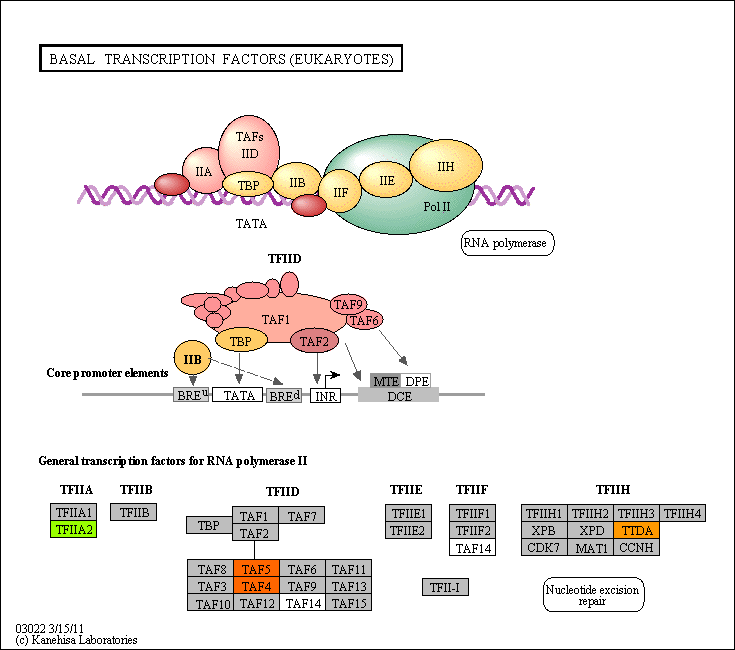


## 2.2 Translation

### RNA Transport

-14 DAYS


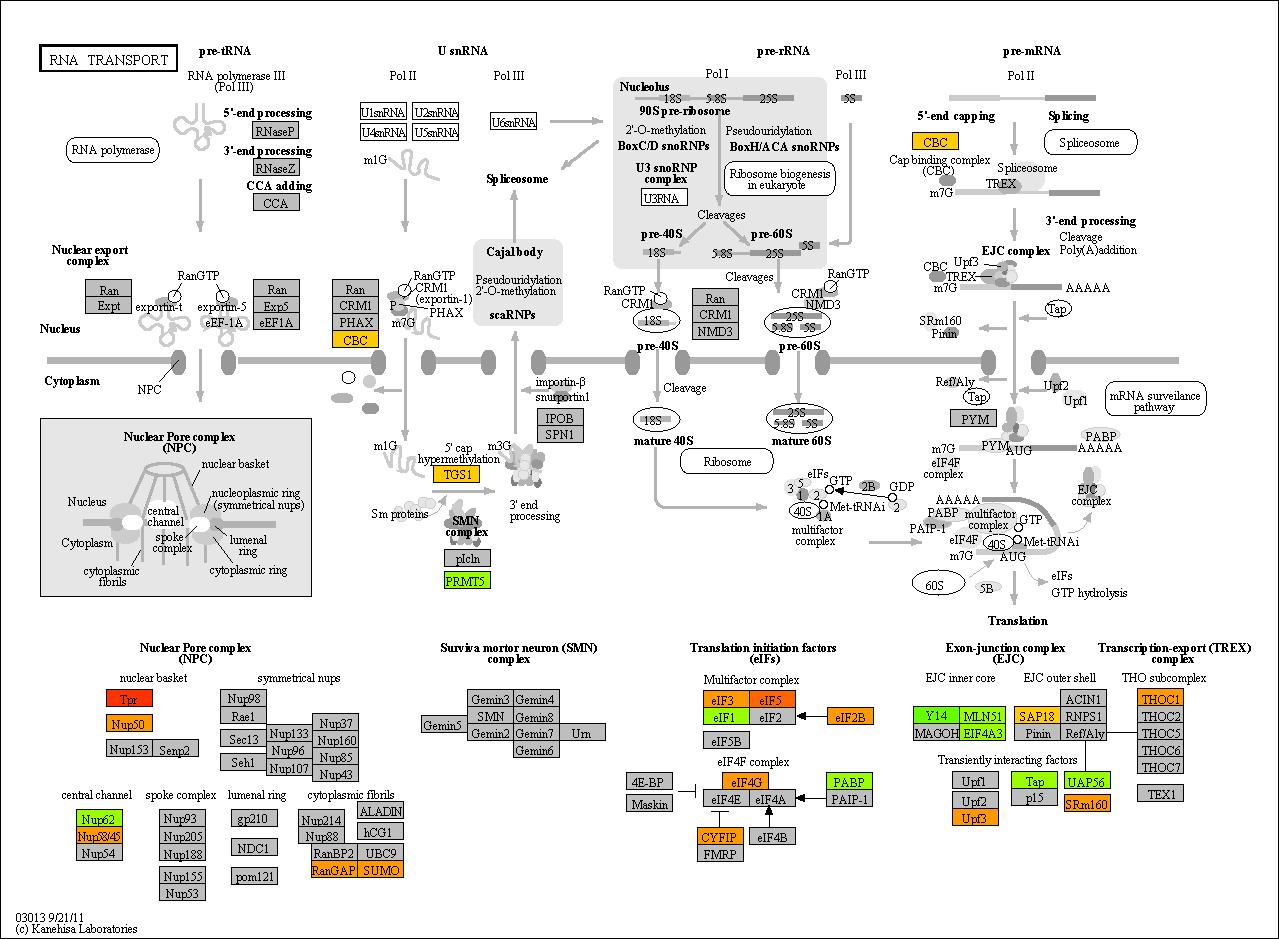


1 DAY 14 DAYS


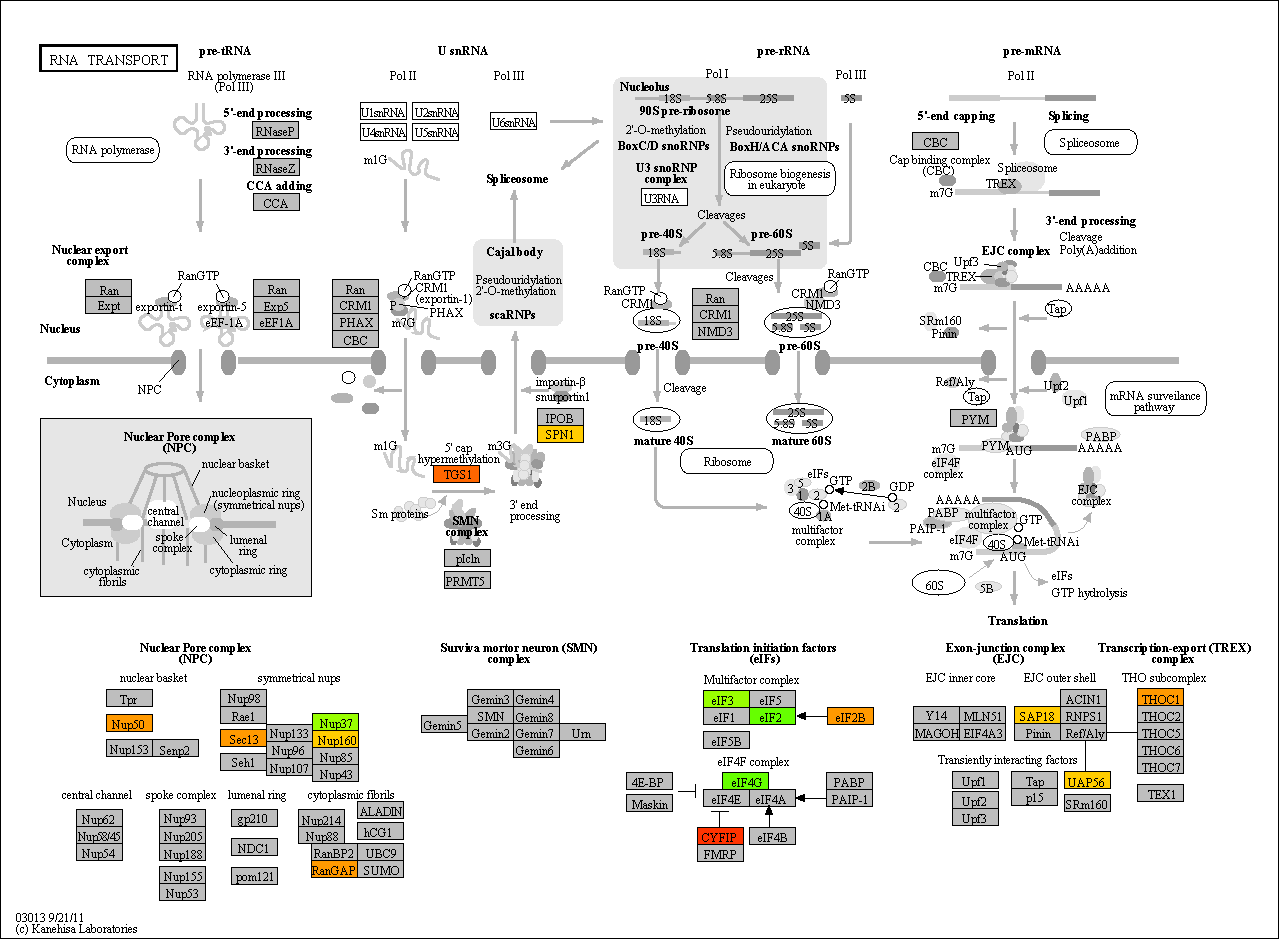

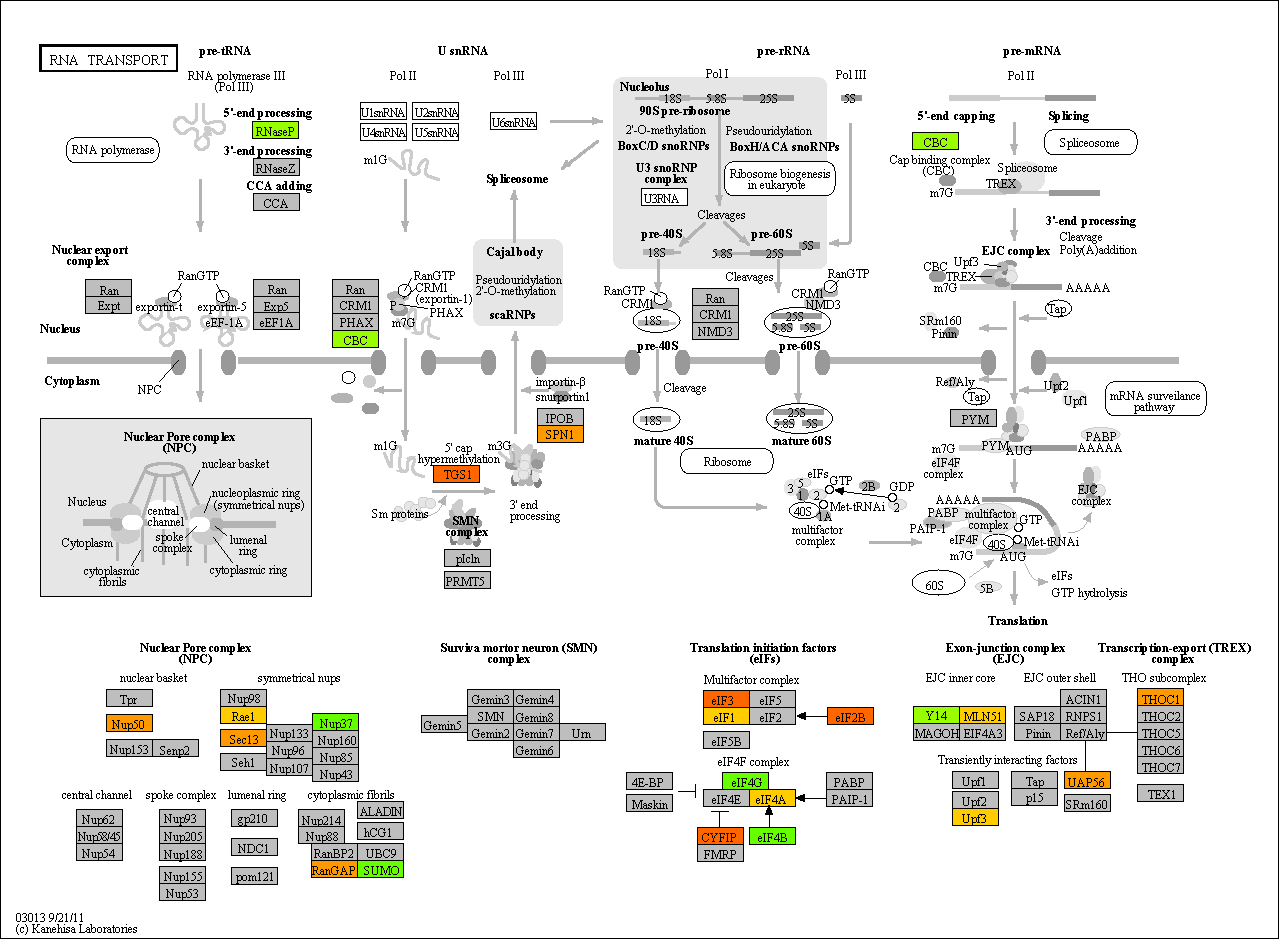


### Ribosome

-14 DAYS


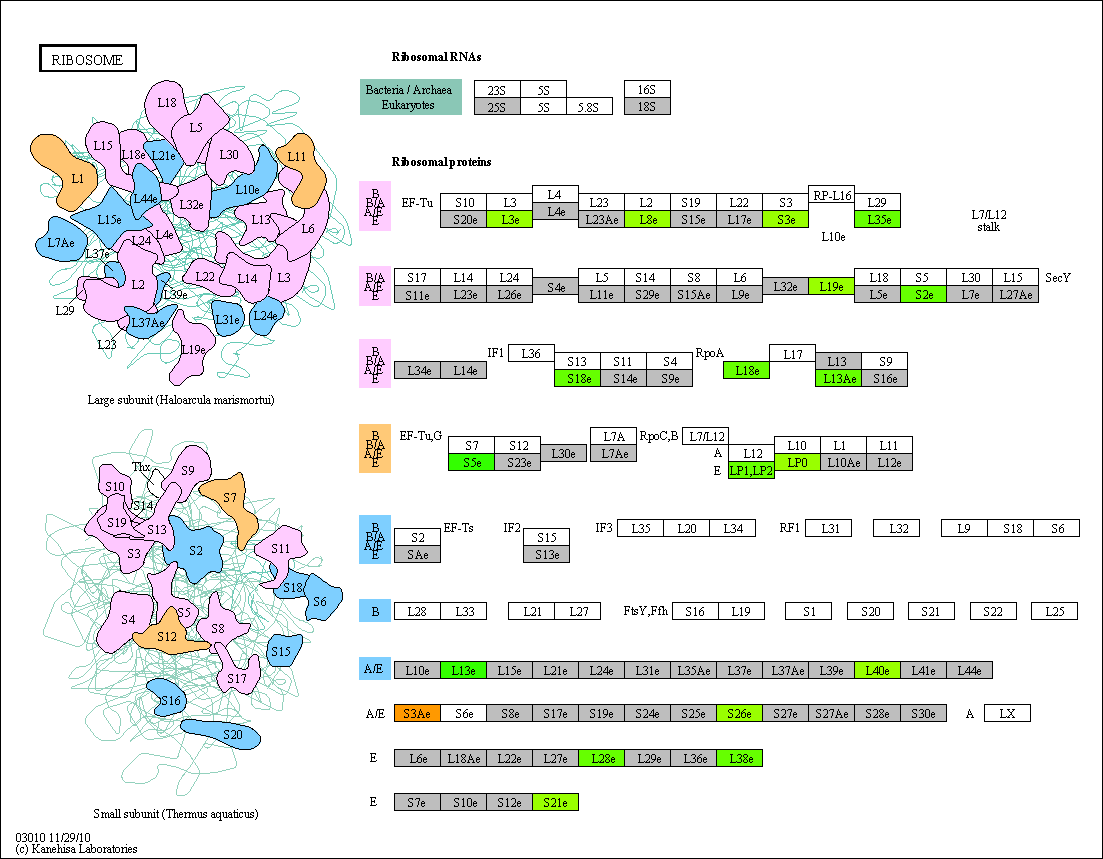


1 DAY 14 DAYS


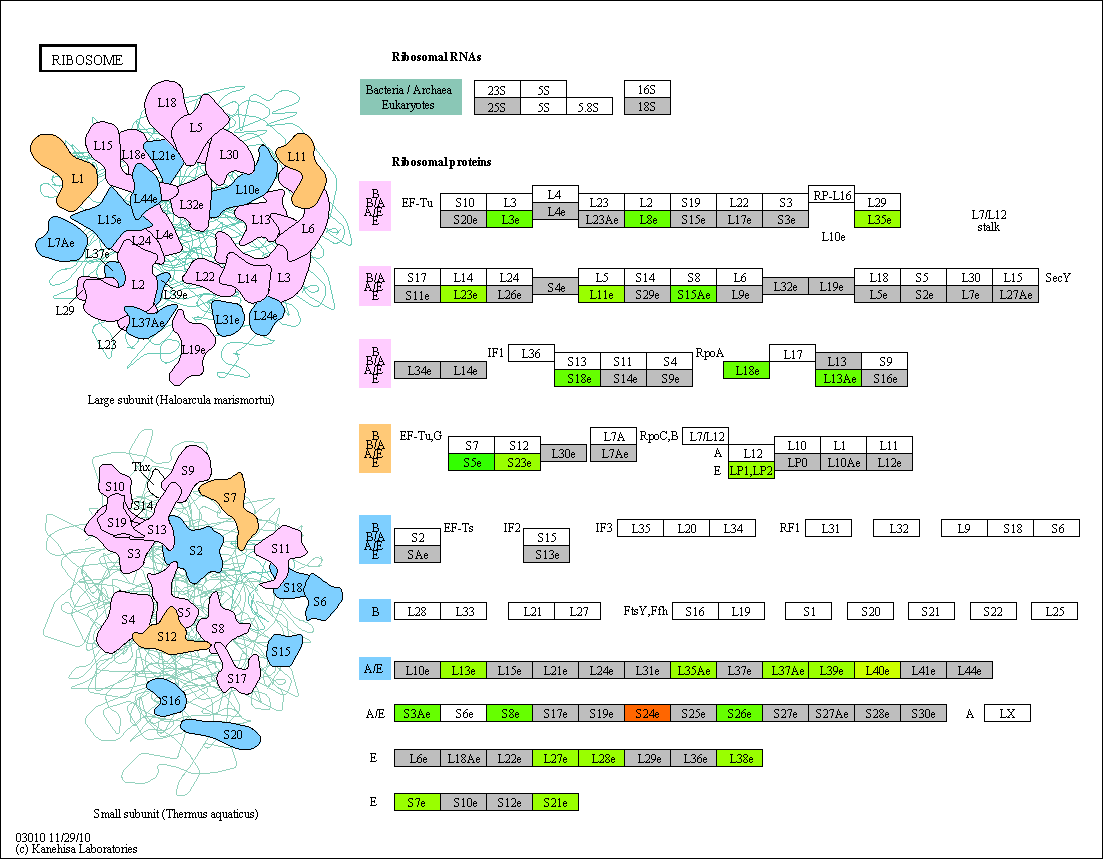

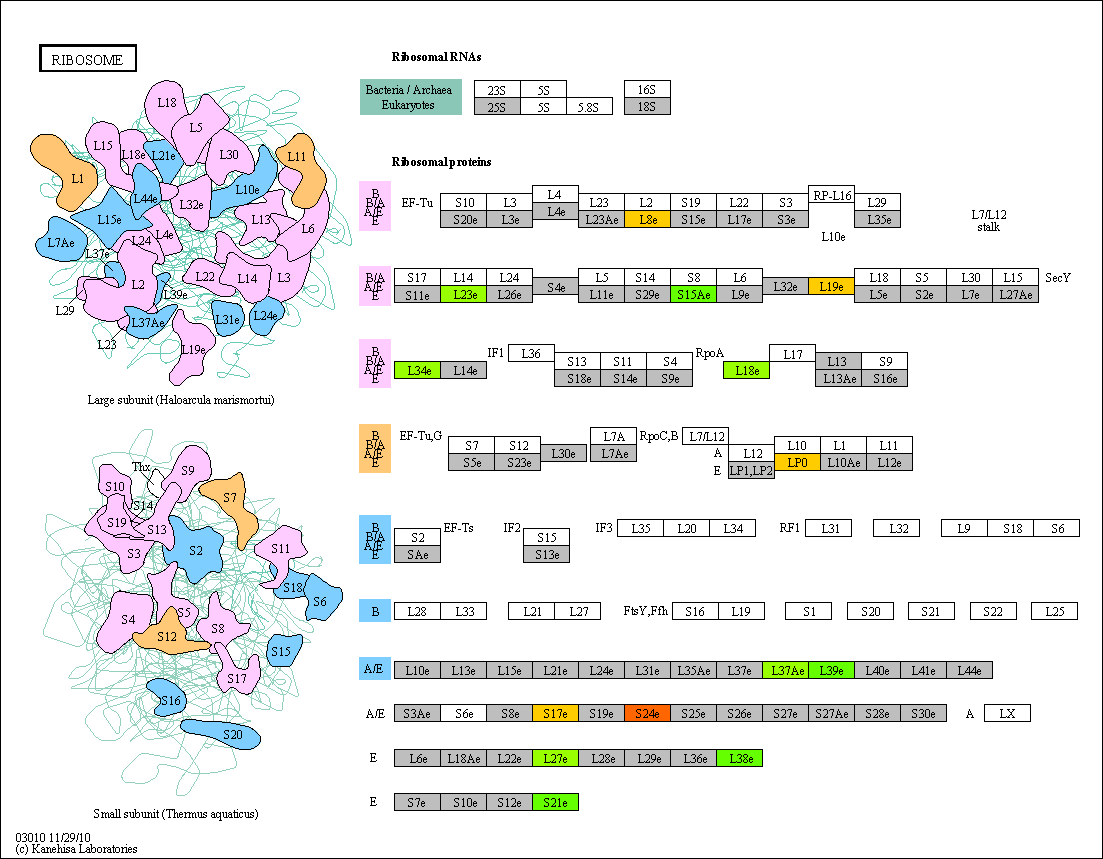


### mRNA Surveillance Pathway

-14 DAYS


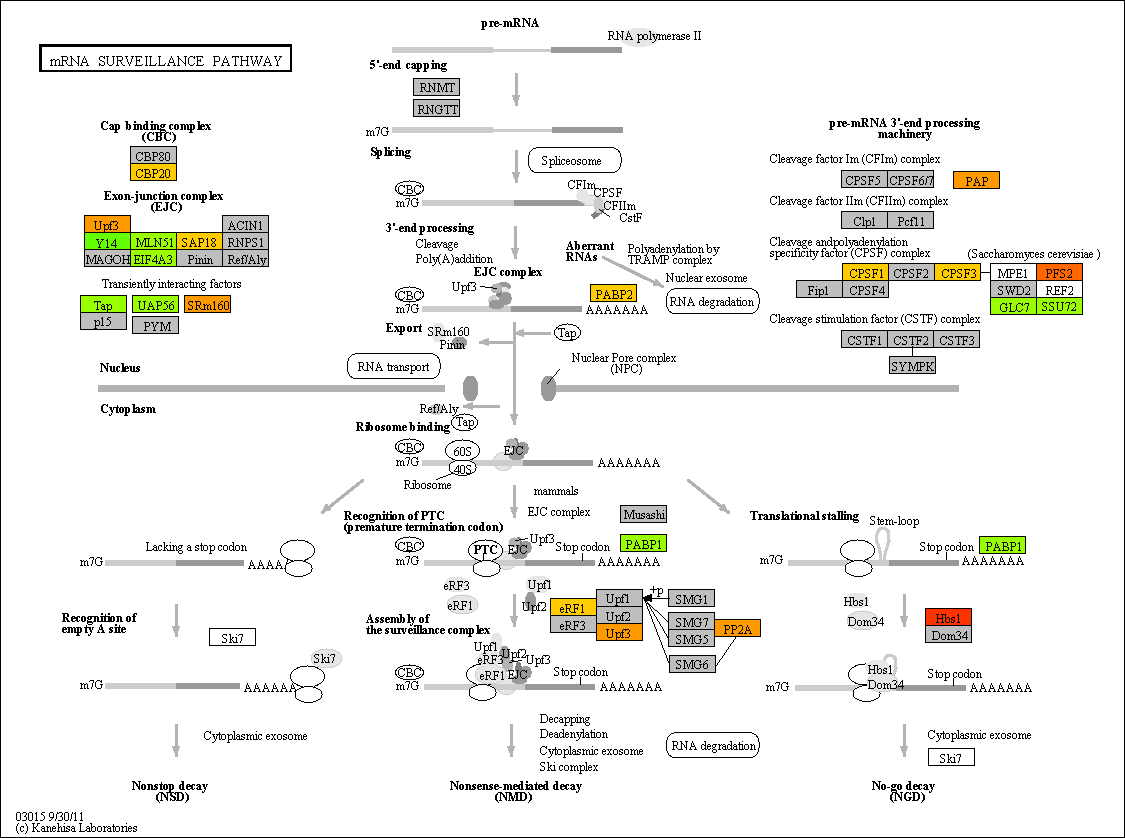


1 DAY 14 DAYS


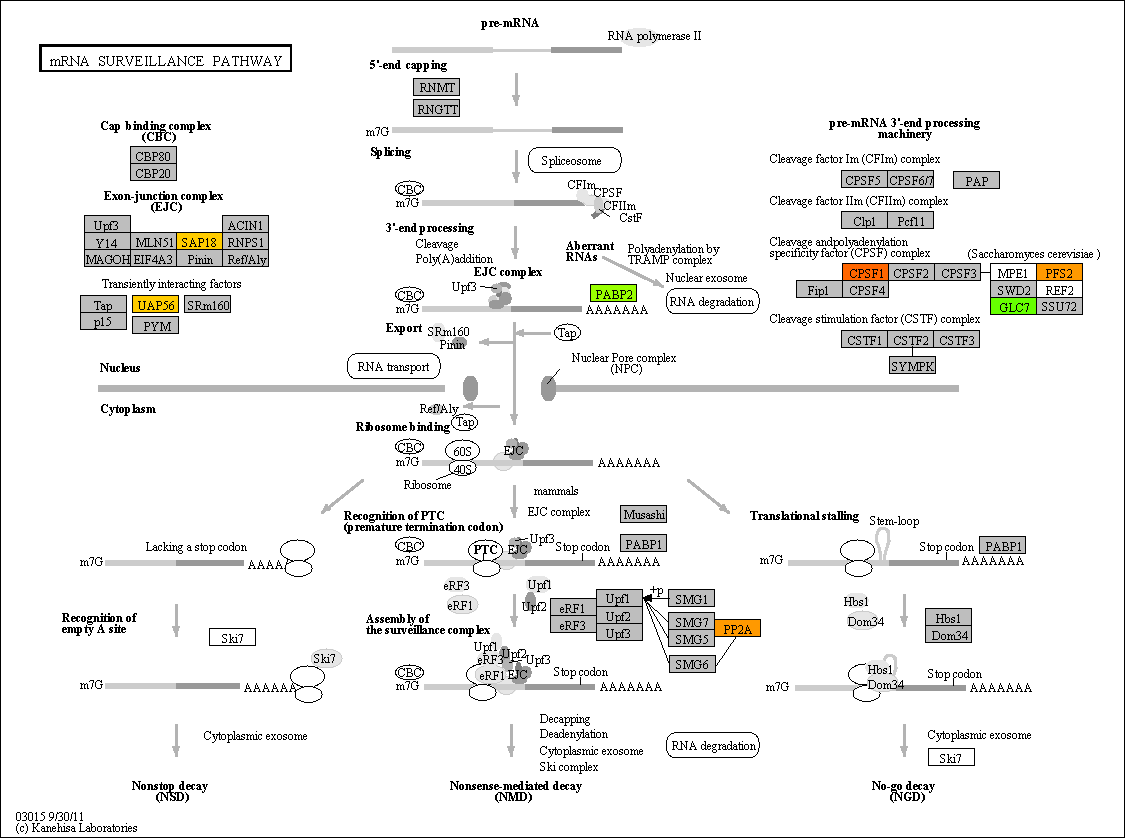

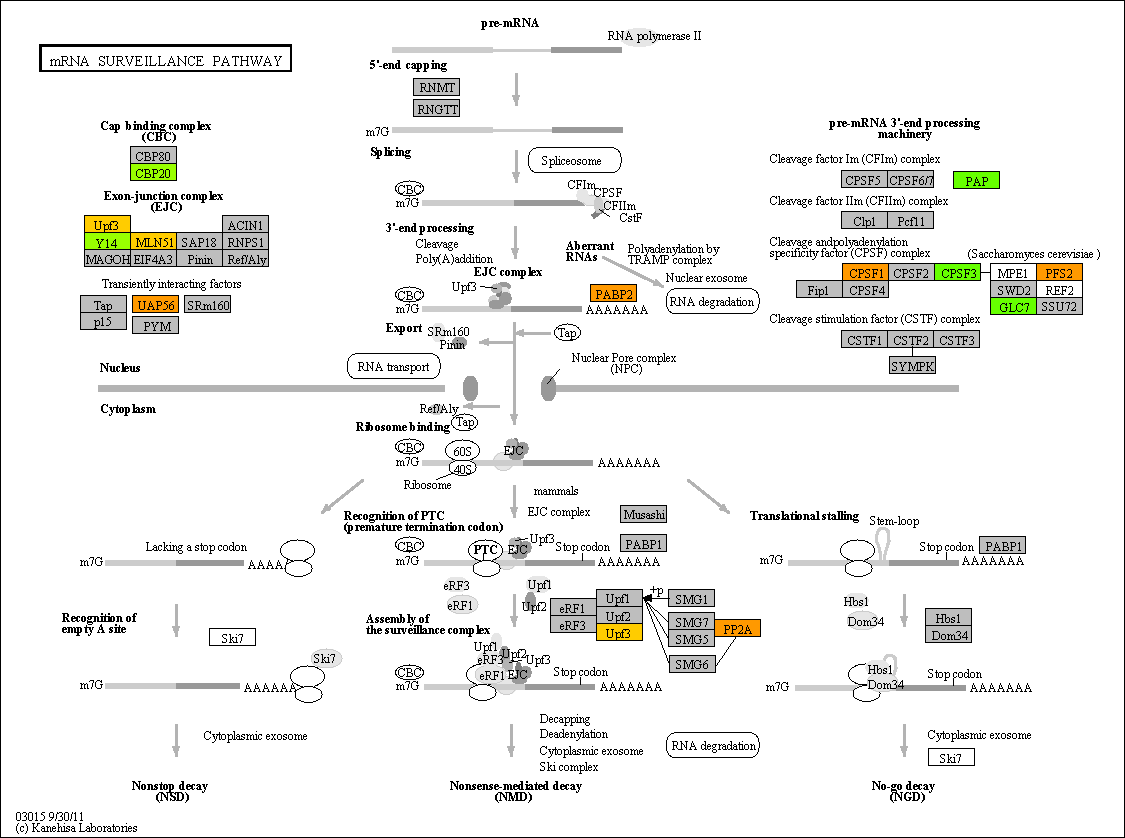


## 2.3 Folding, Sorting and Degradation

### Protein Export

-14 DAYS


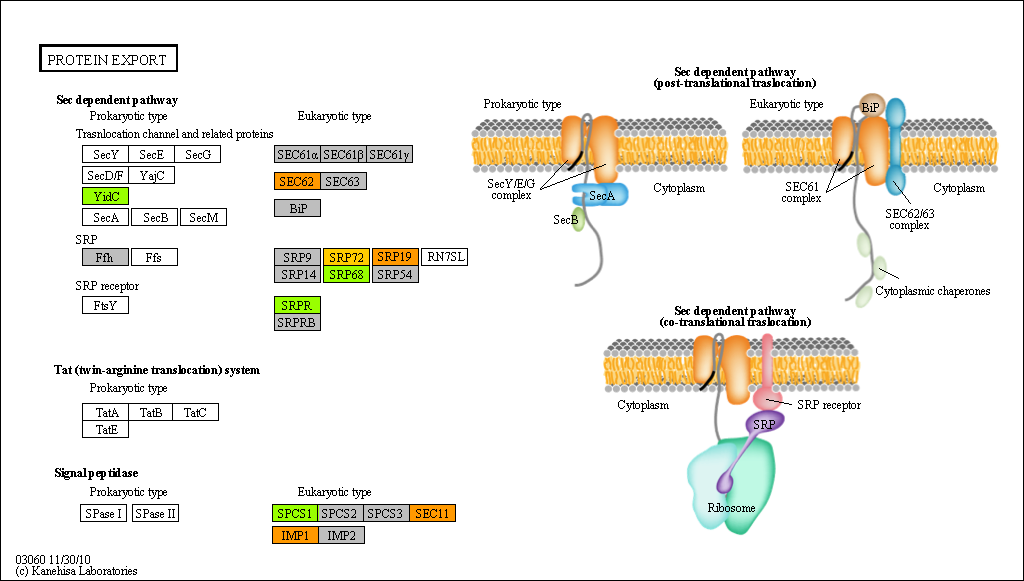


1 DAY 14 DAYS


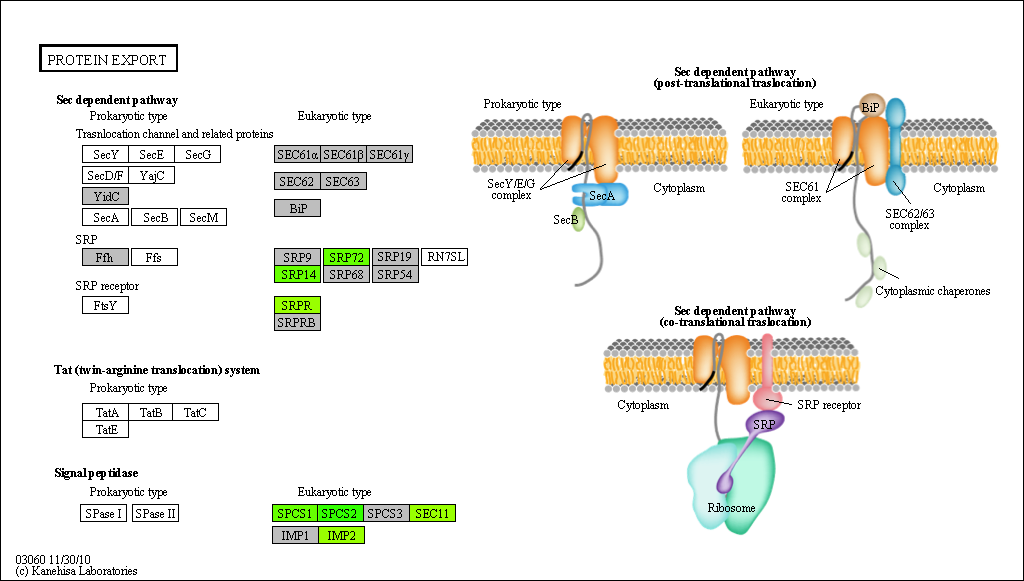

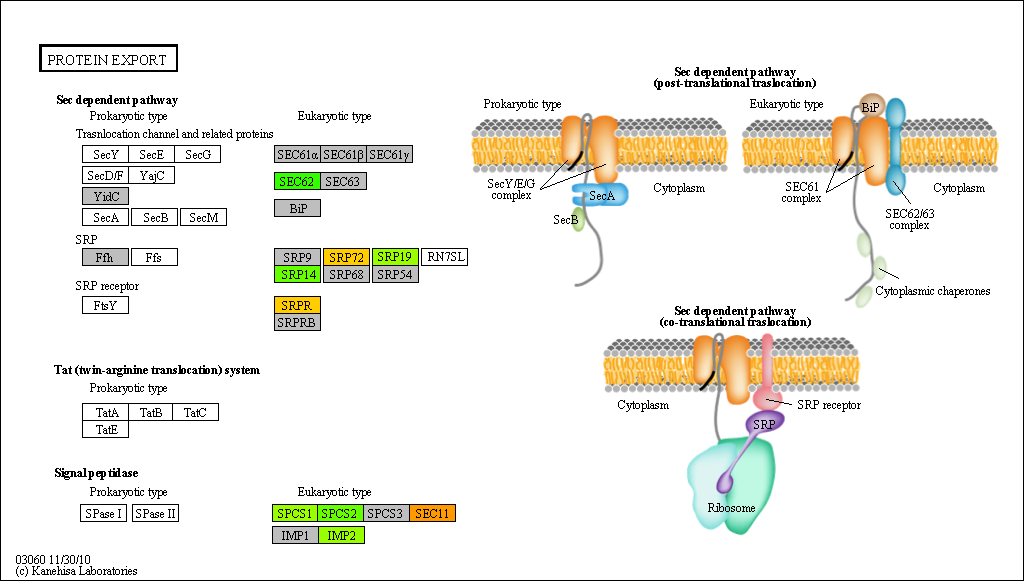


### Protein Processing in Endoplasmic Reticulum

-14 DAYS


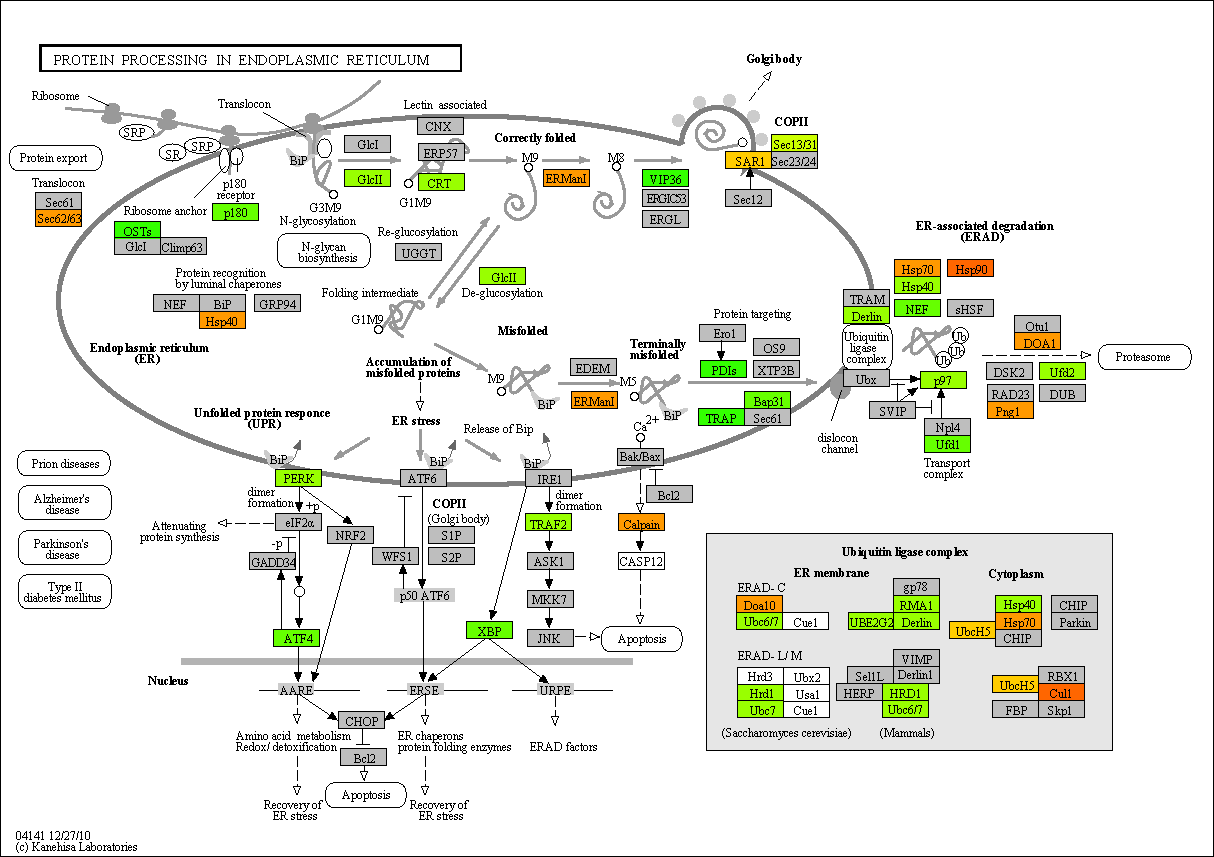


1 DAY 14 DAYS


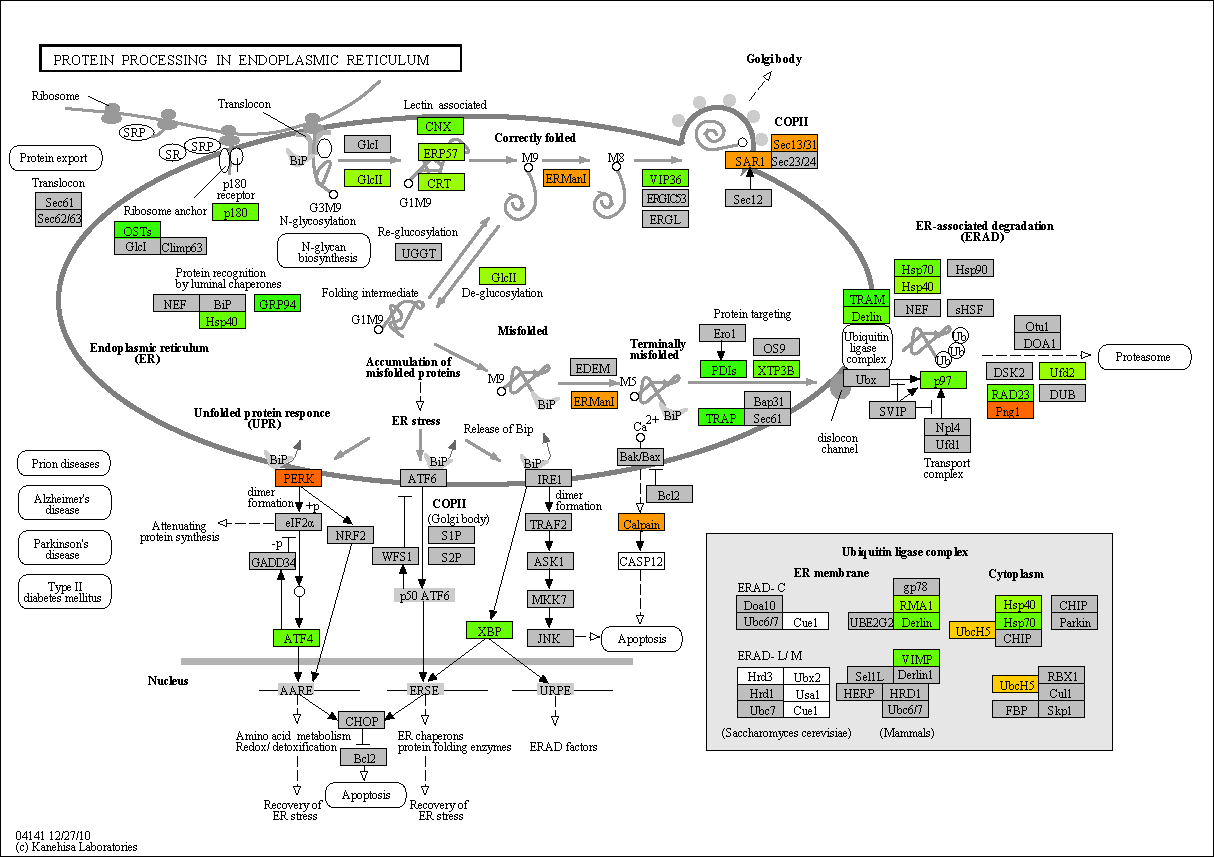

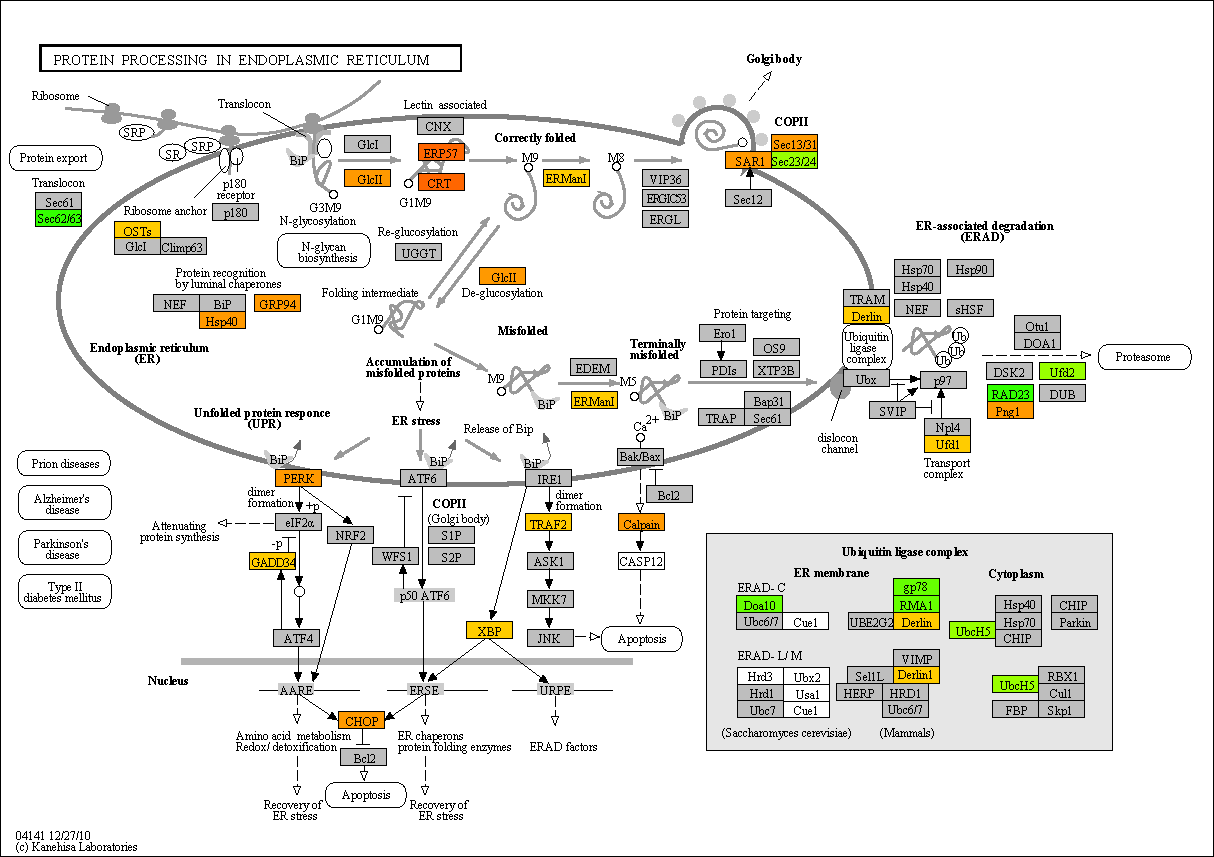


### Ubiquitin Mediated Proteolysis

-14 DAYS


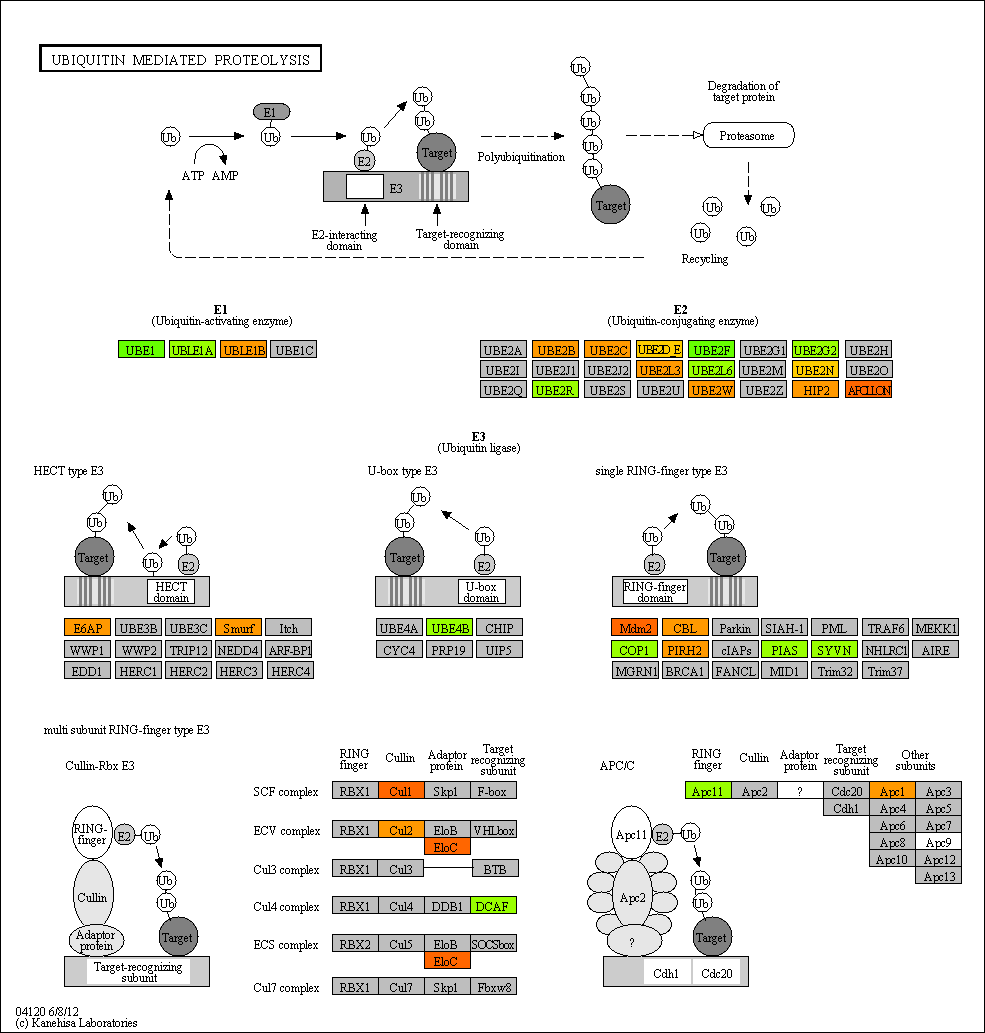


1 DAY 14 DAYS


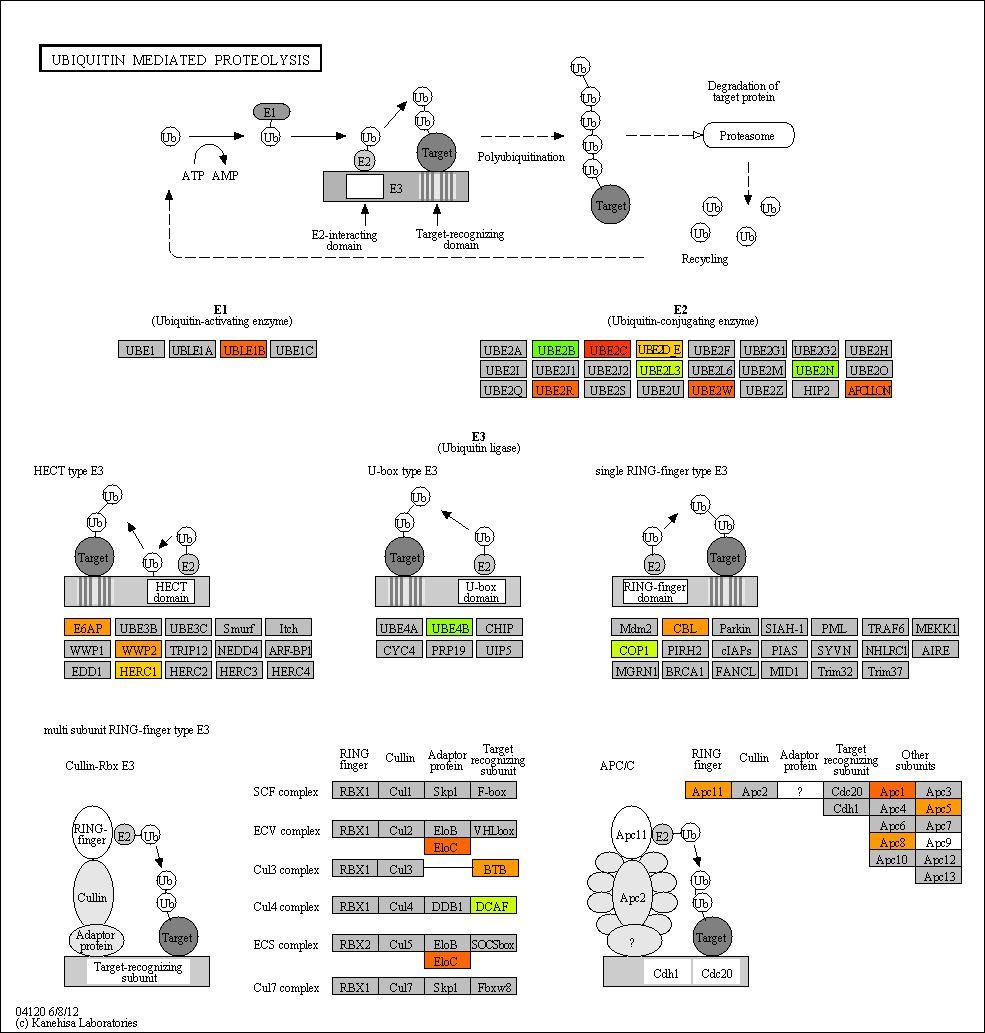

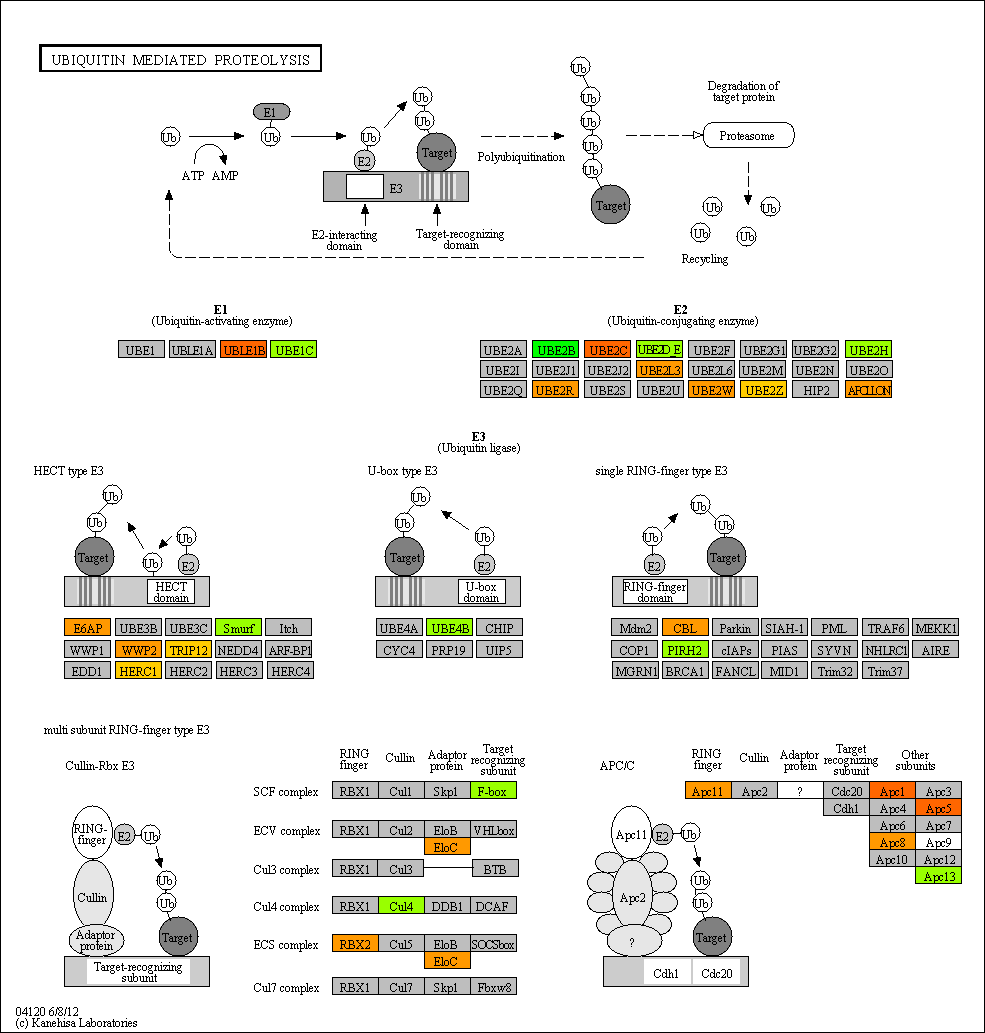


## 2.4 Replication and Repair

### Base Excision Repair

-14 DAYS


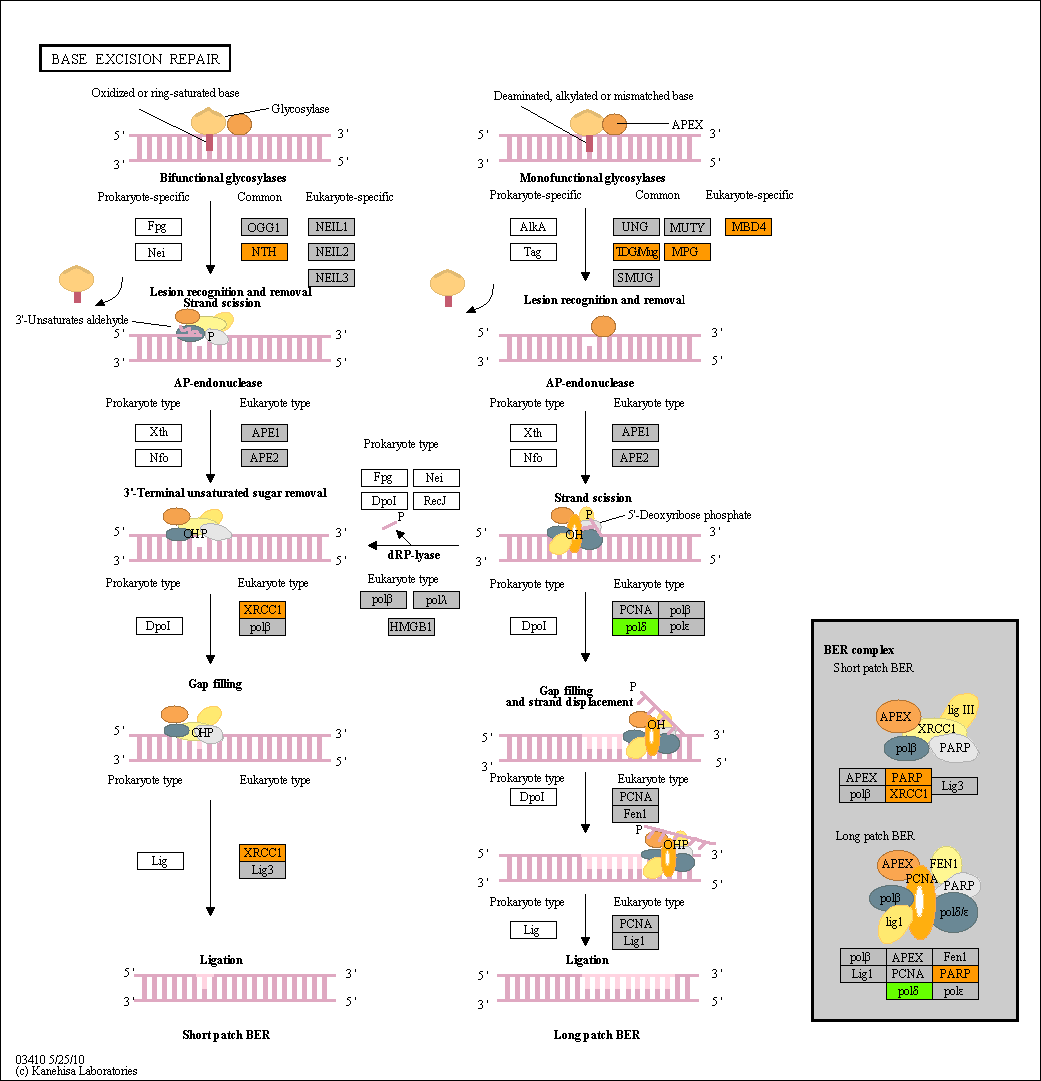


1 DAY 14 DAYS


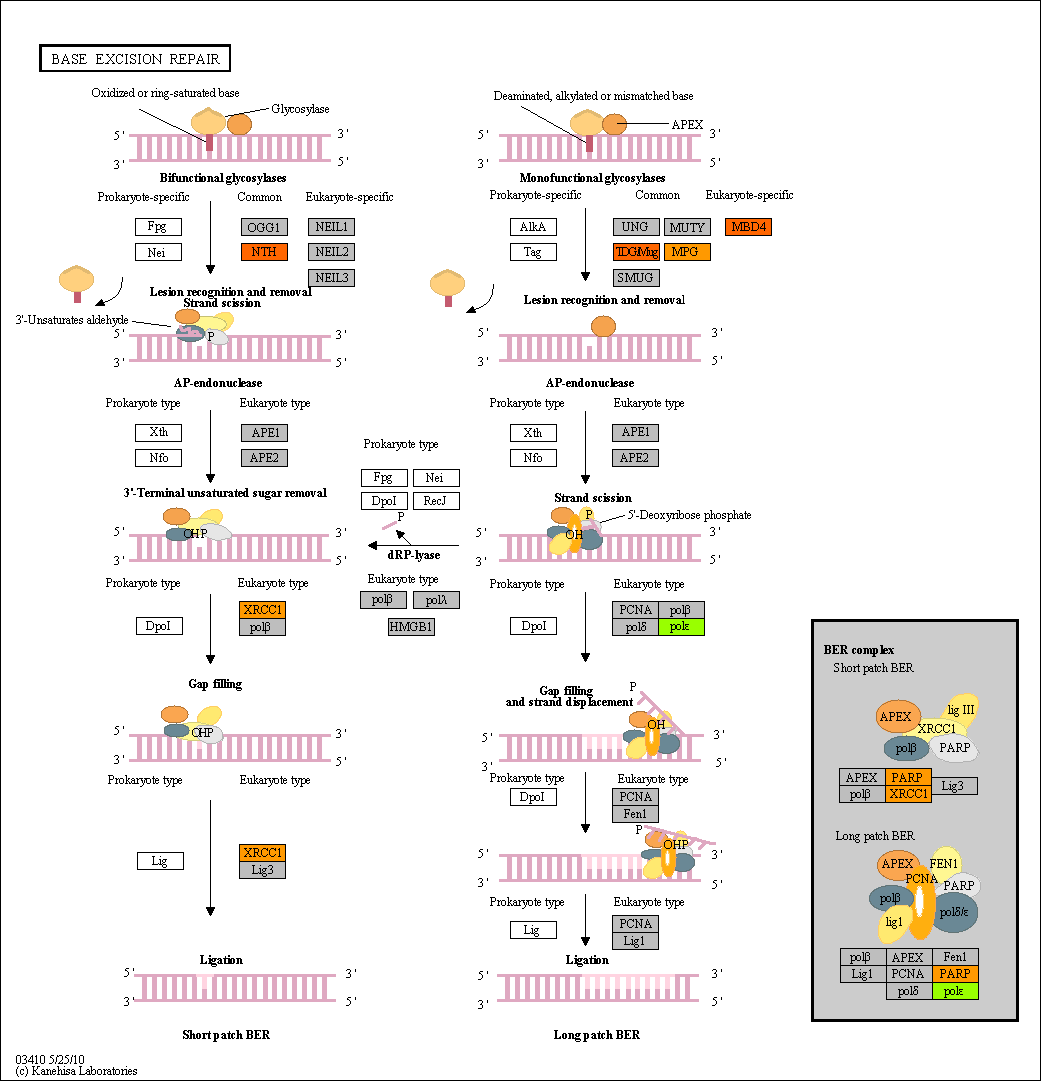

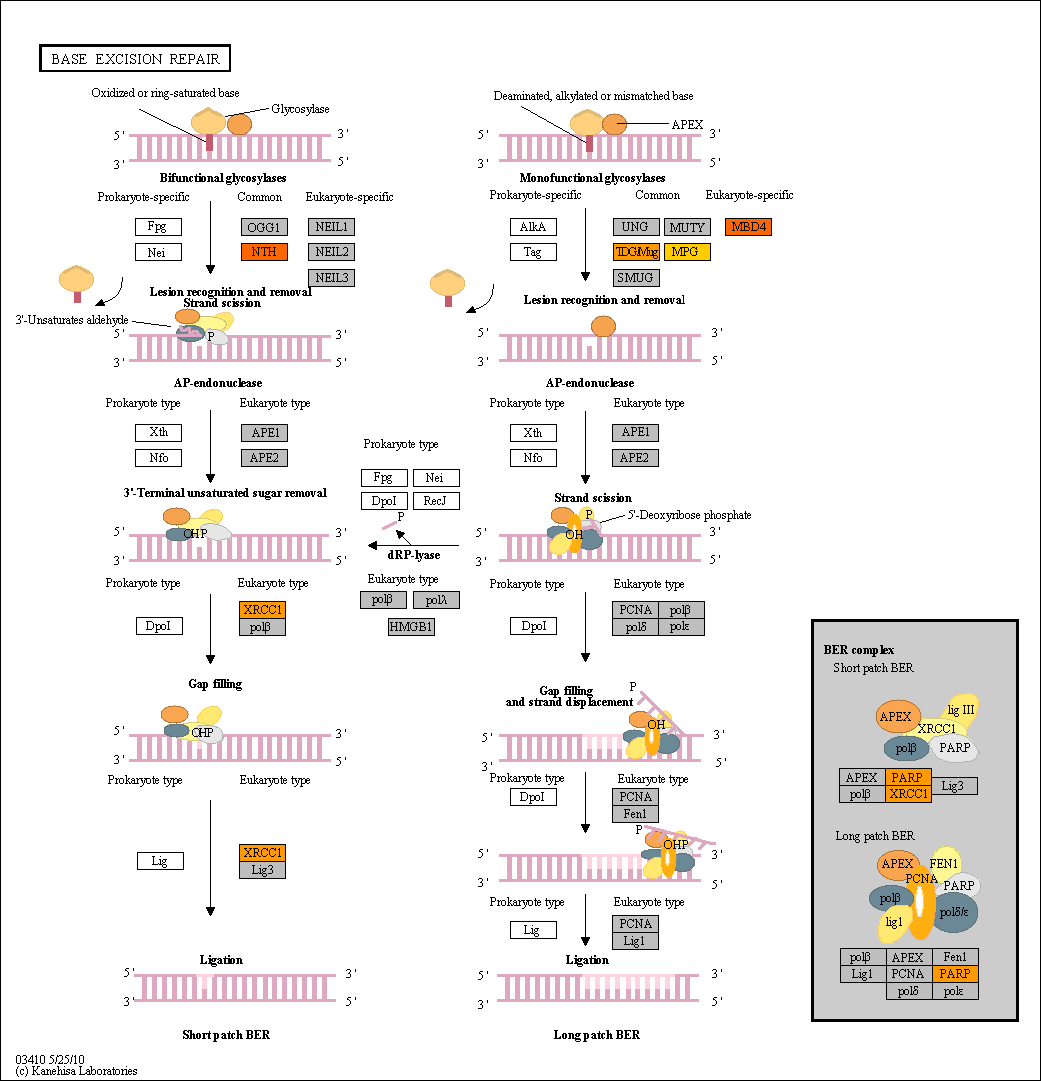


### Non-homologous end-joining

-14 DAYS


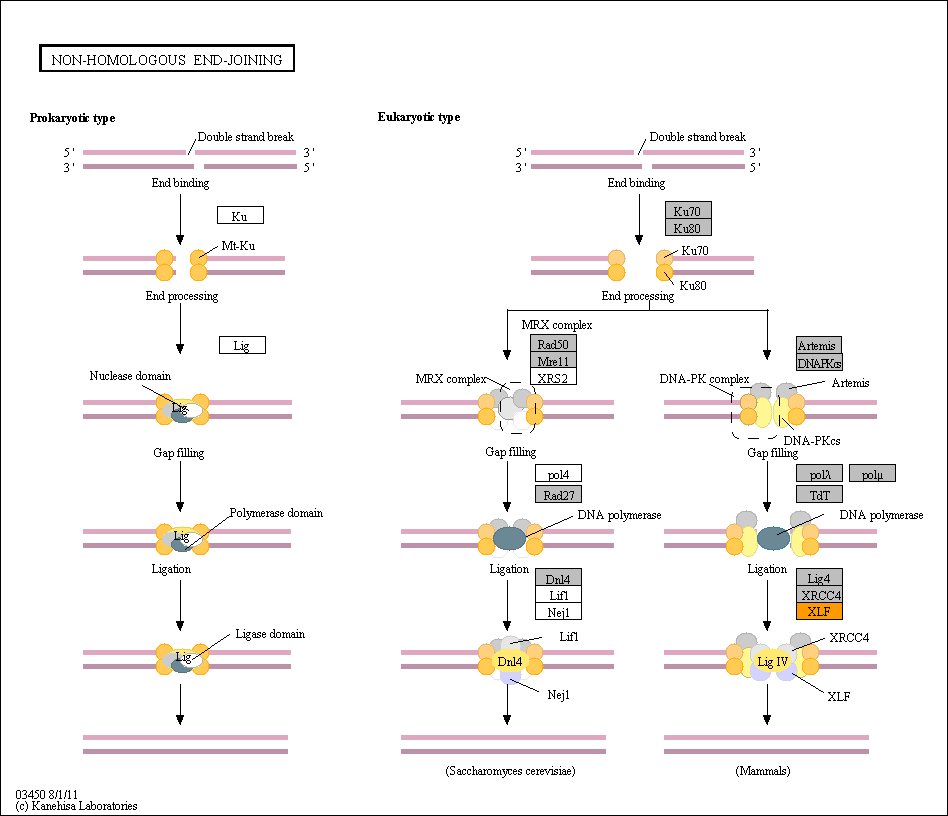


1 DAY 14 DAYS


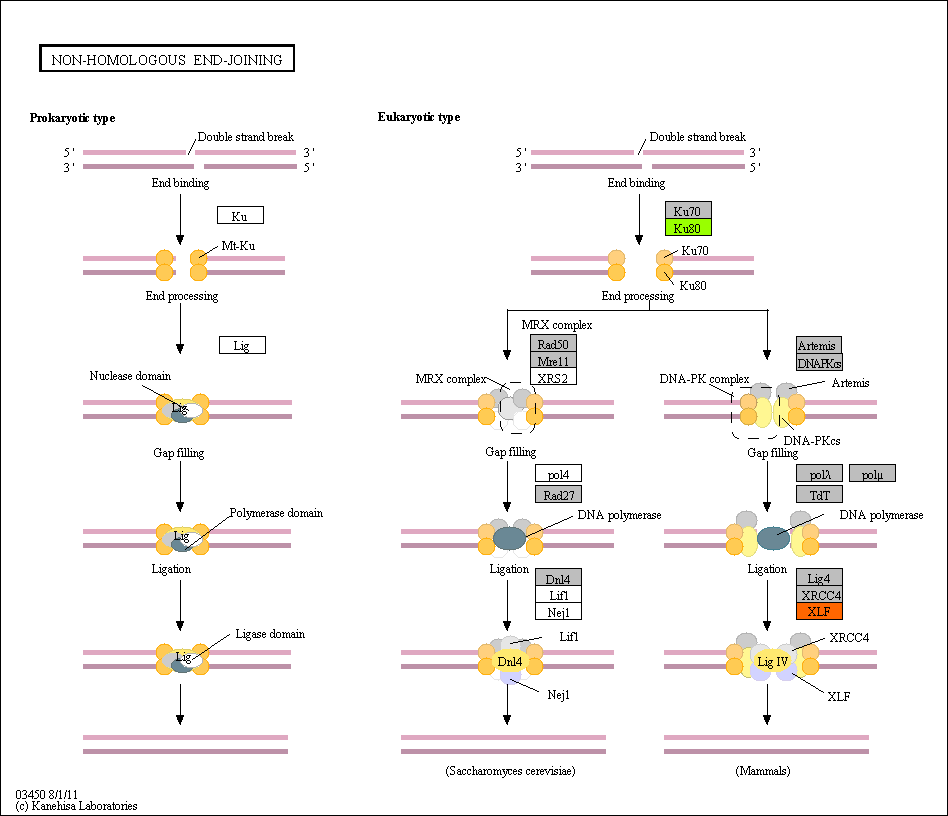

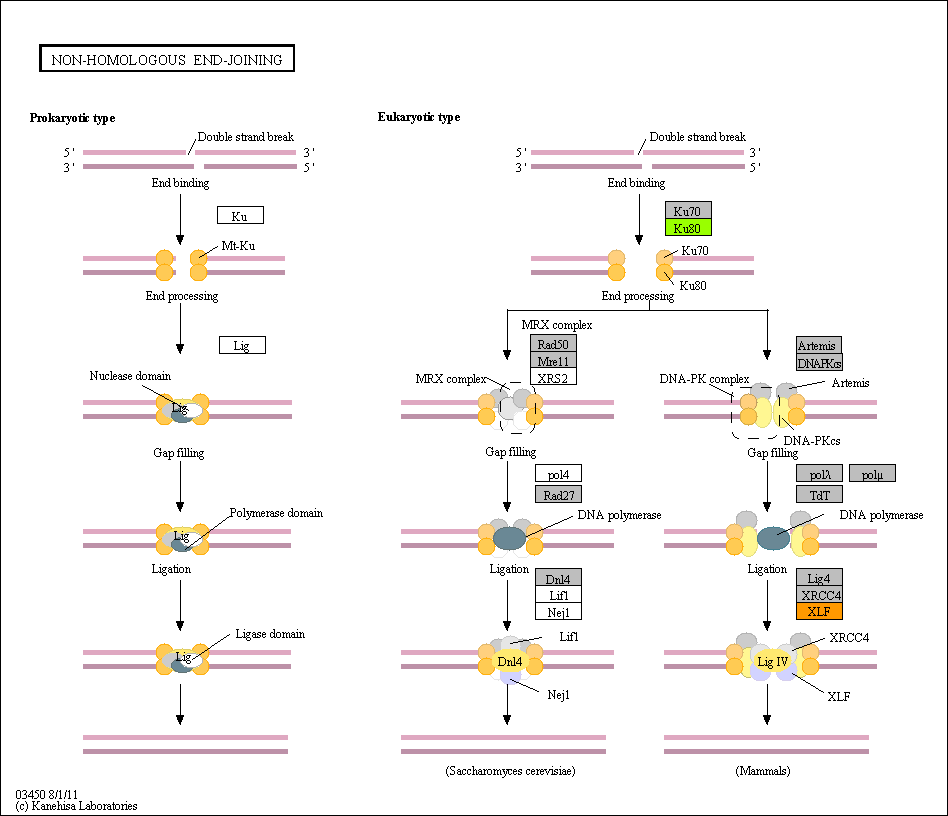


# 3. Environmental Information Processing

## 3.1 Membrane Transport

### ABC transporters


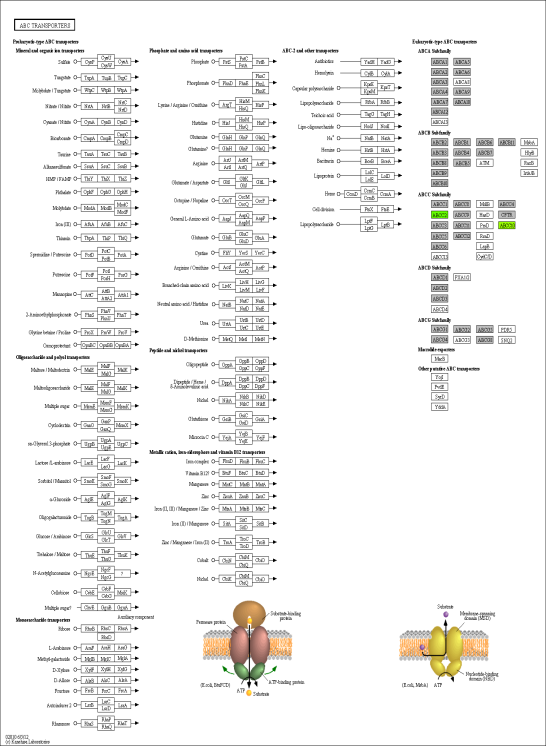
-14 DAYS 1 DAY


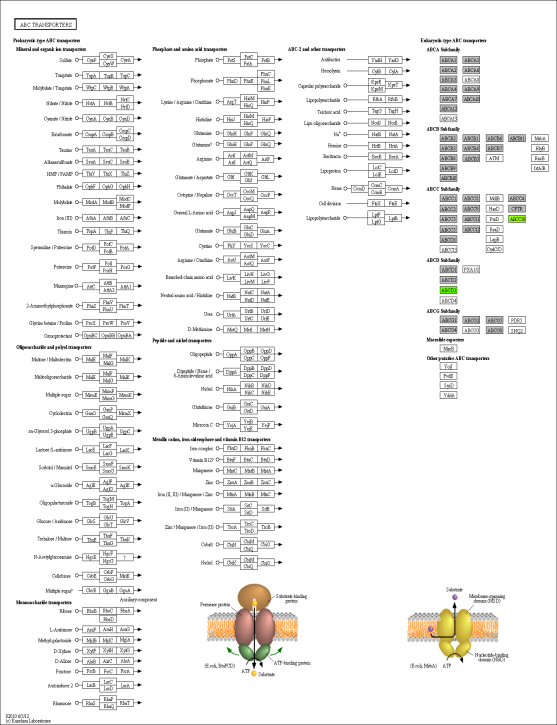

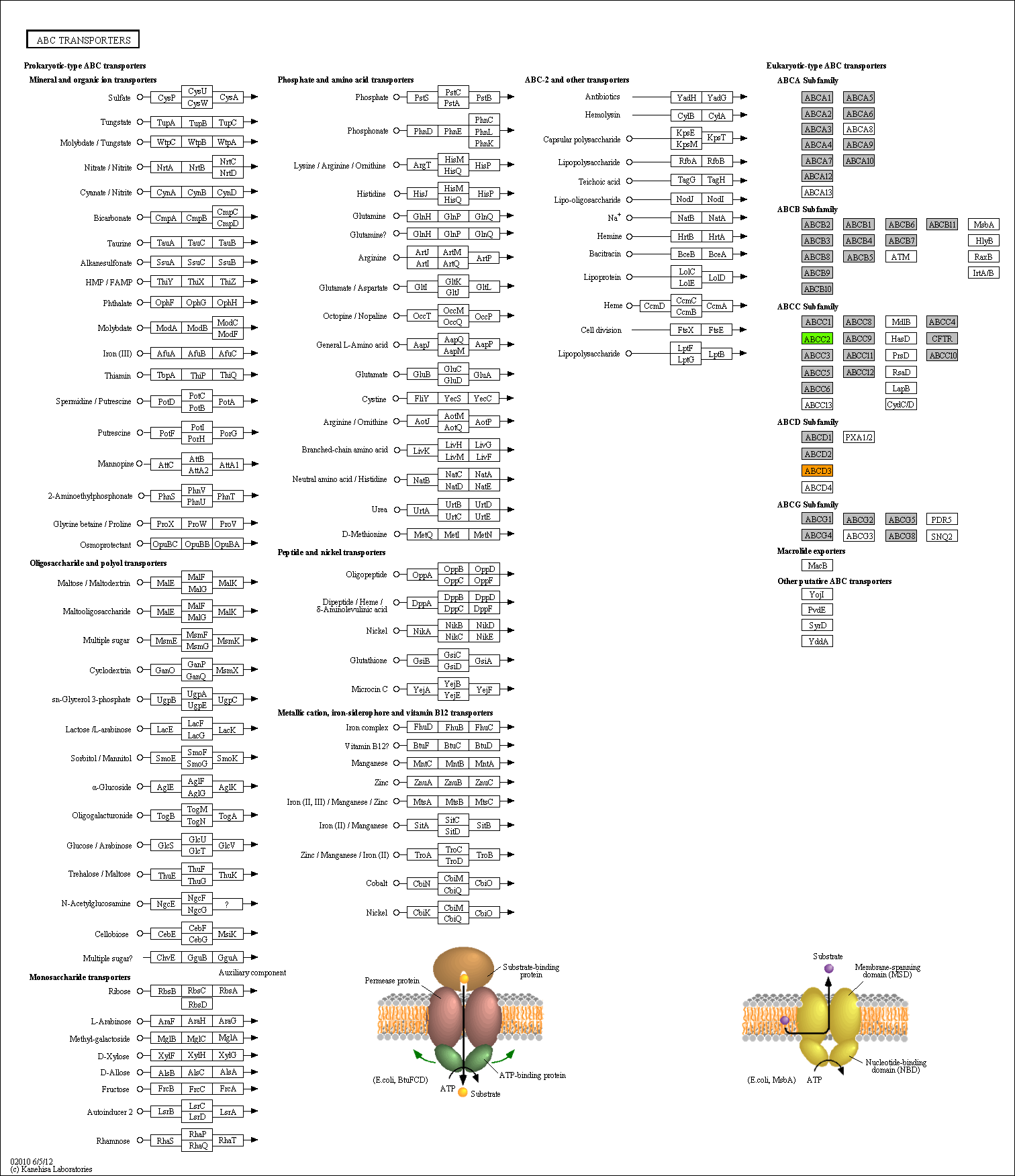
 14 DAYS

## 3.2 Signal Transduction

### Notch signaling

-14 DAYS


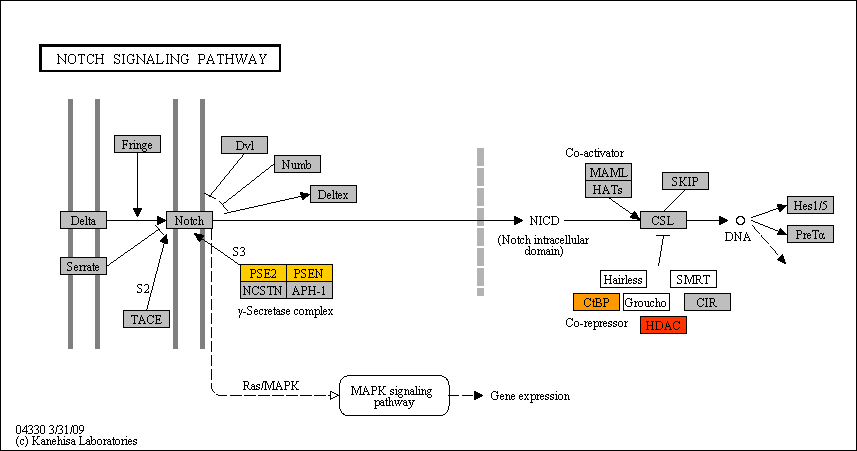


1 DAY 14 DAYS


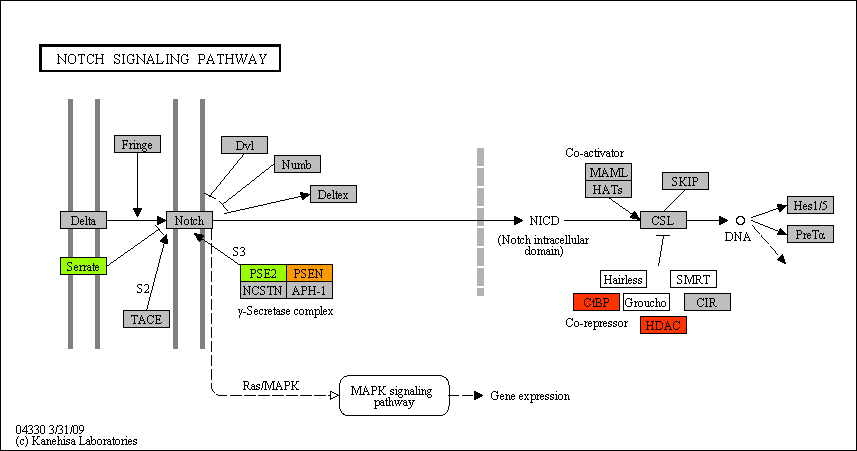

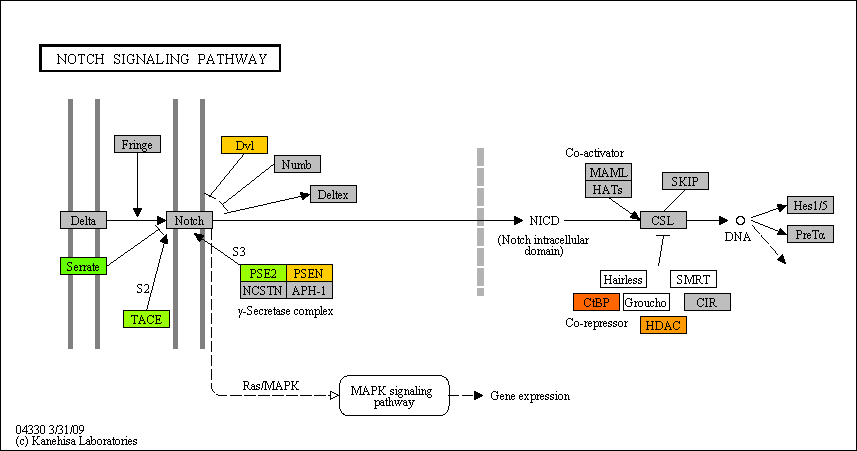


## 3.3 Signaling Molecules and Interaction

### ECM-receptor Interaction

-14 DAYS


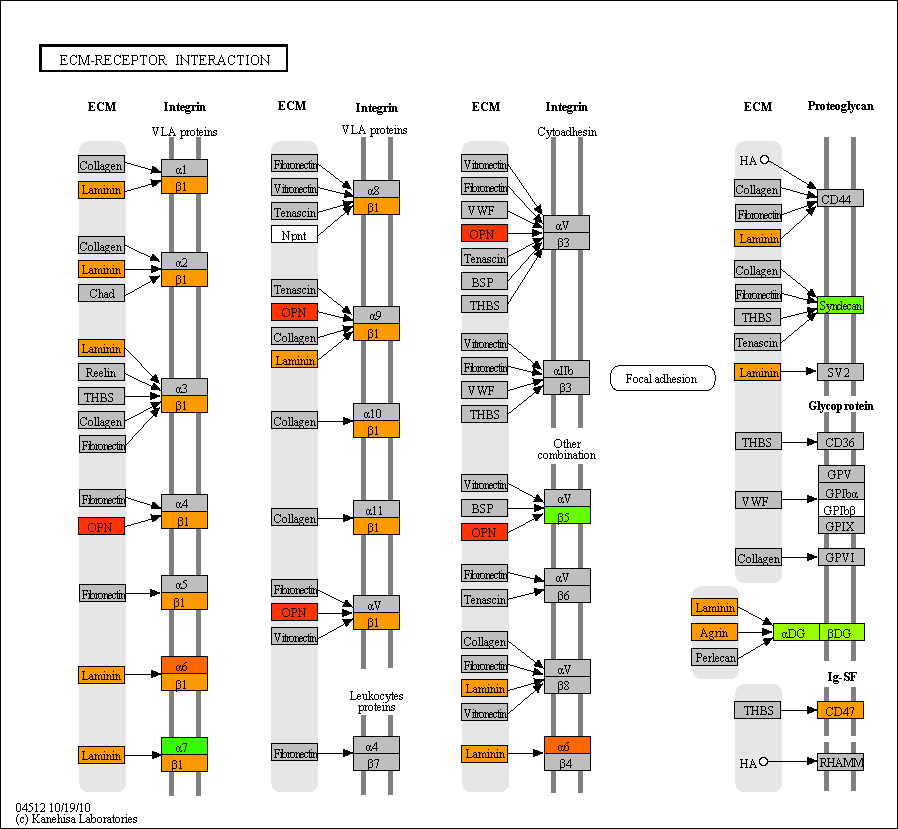


1 DAY 14 DAYS


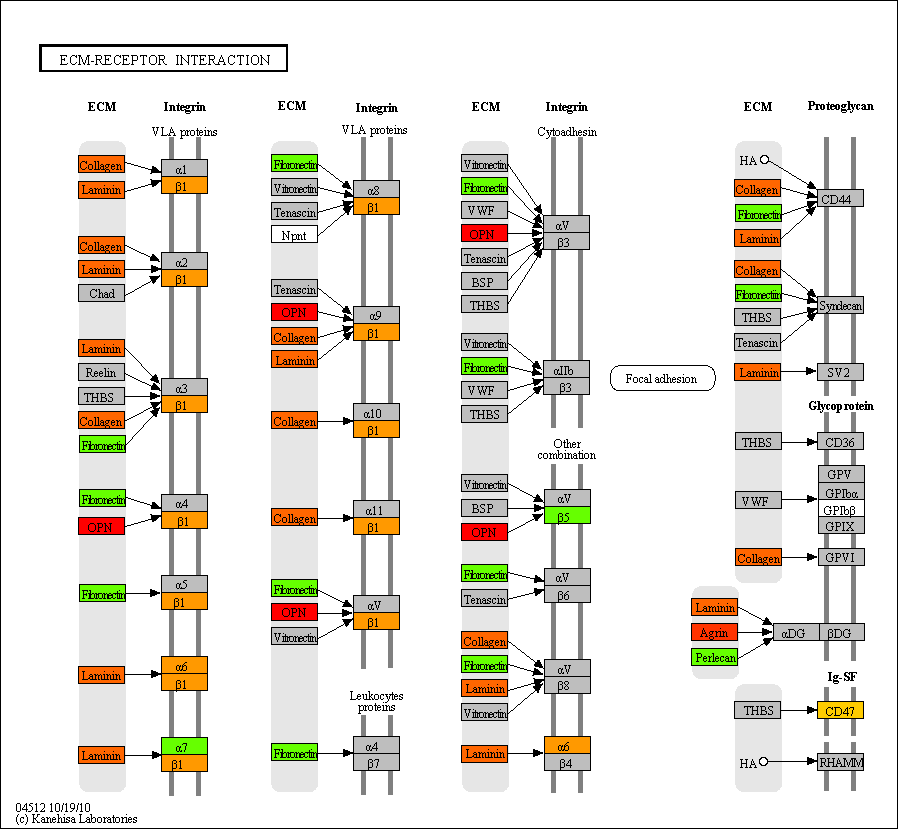

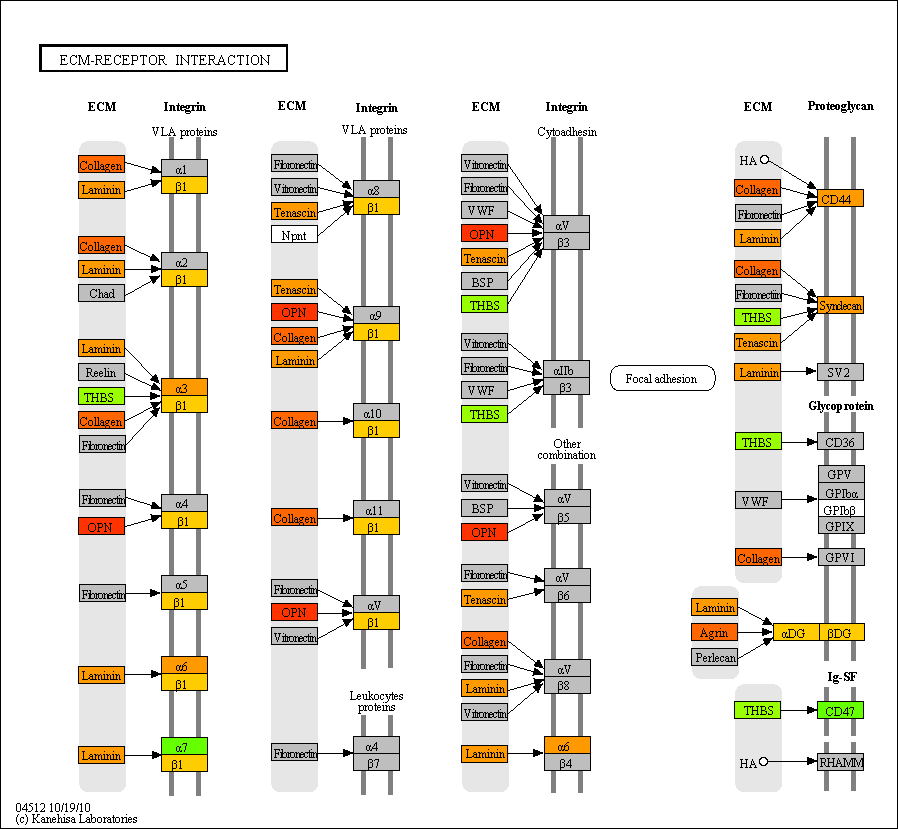


# 4. Cellular Processes

## 4.1 Transport and catabolism

### Peroxisome

-14 DAYS


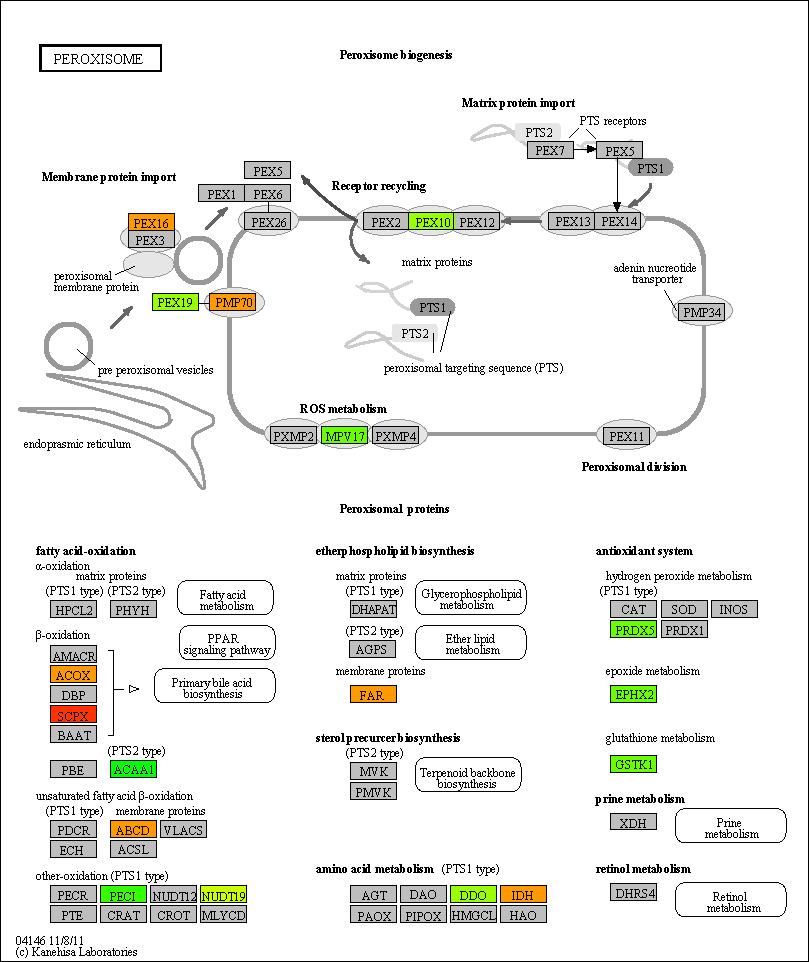


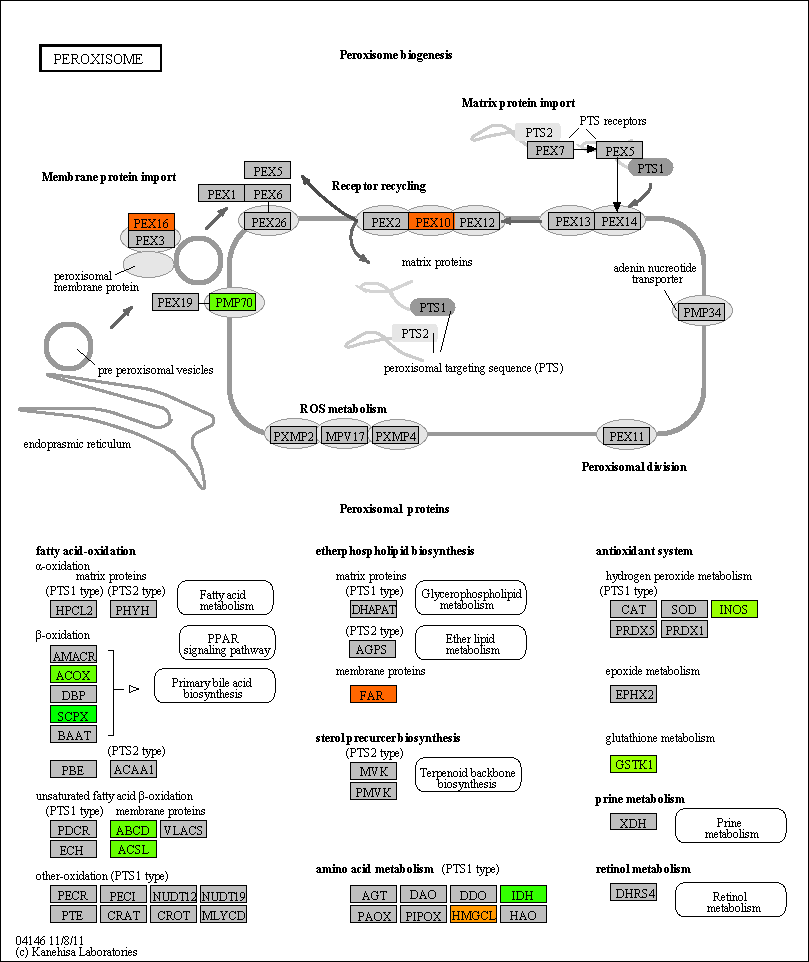

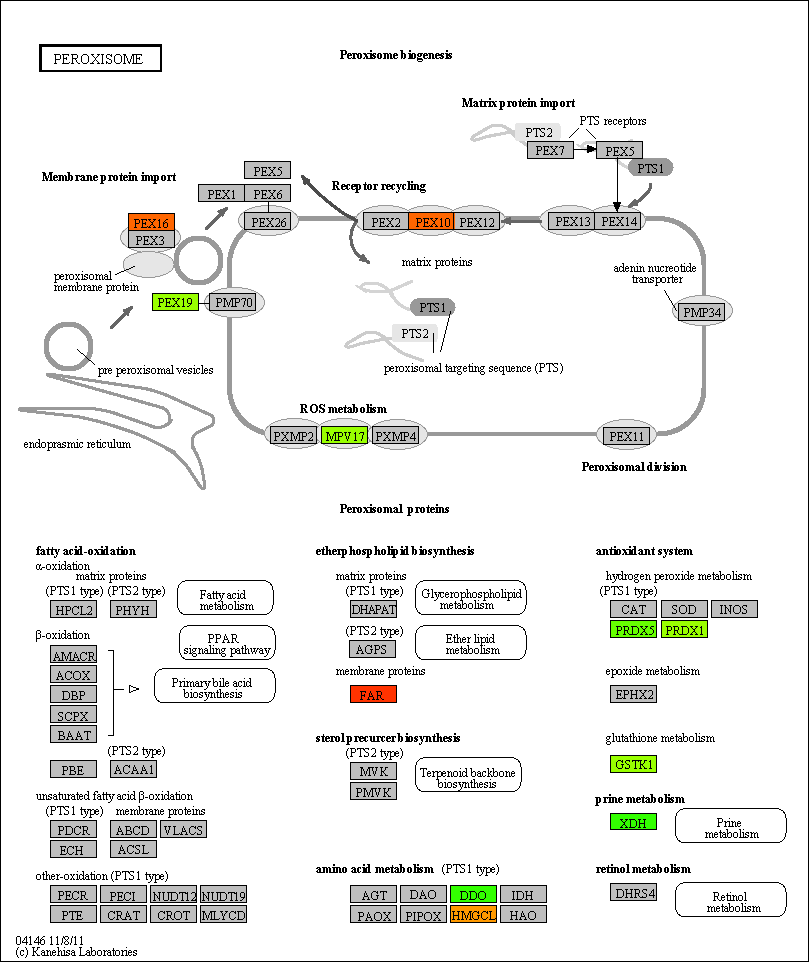
 1 DAY 14 DAYS

### Lysosome

-14 DAYS


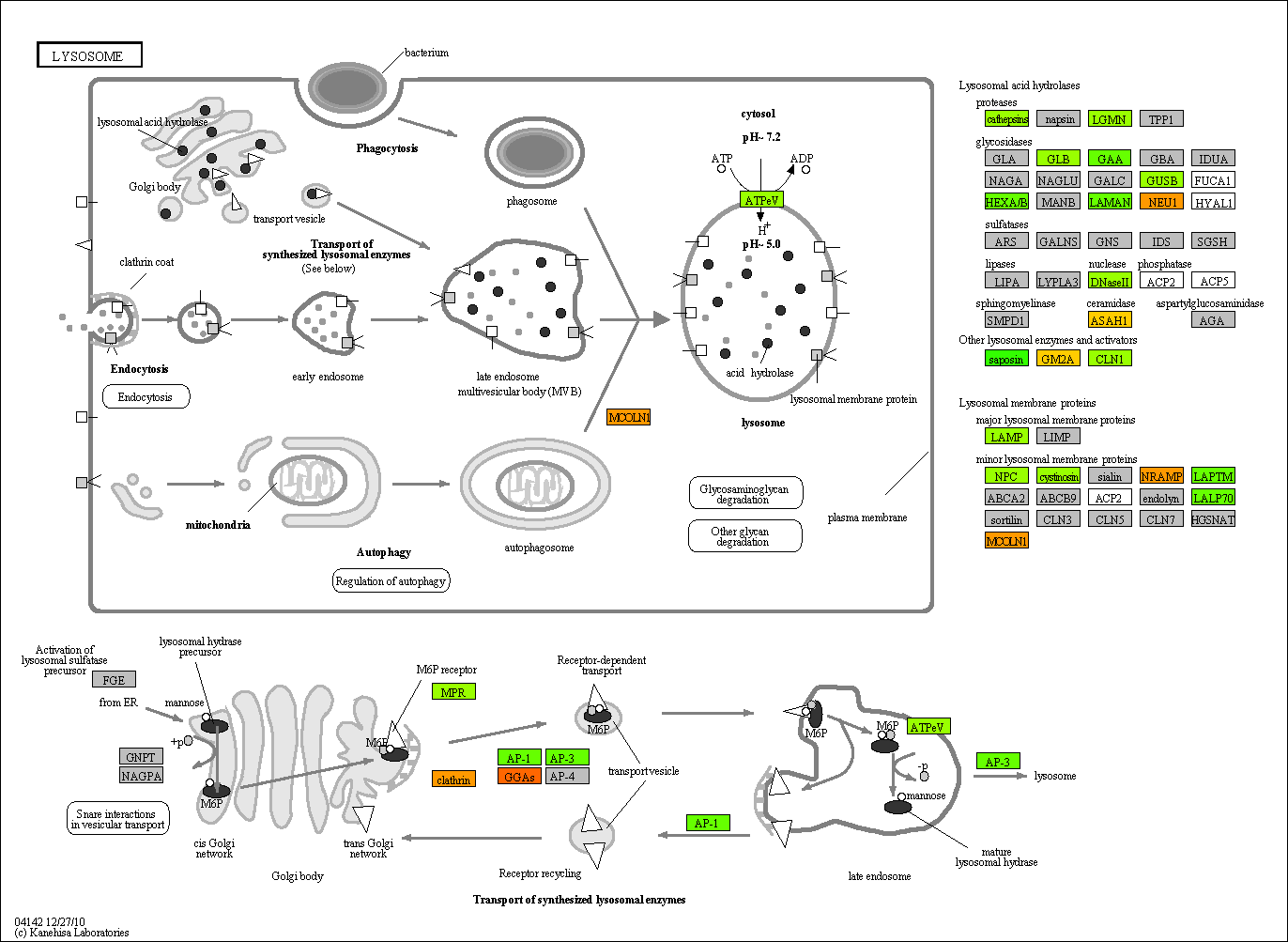


1 DAY 14 DAYS


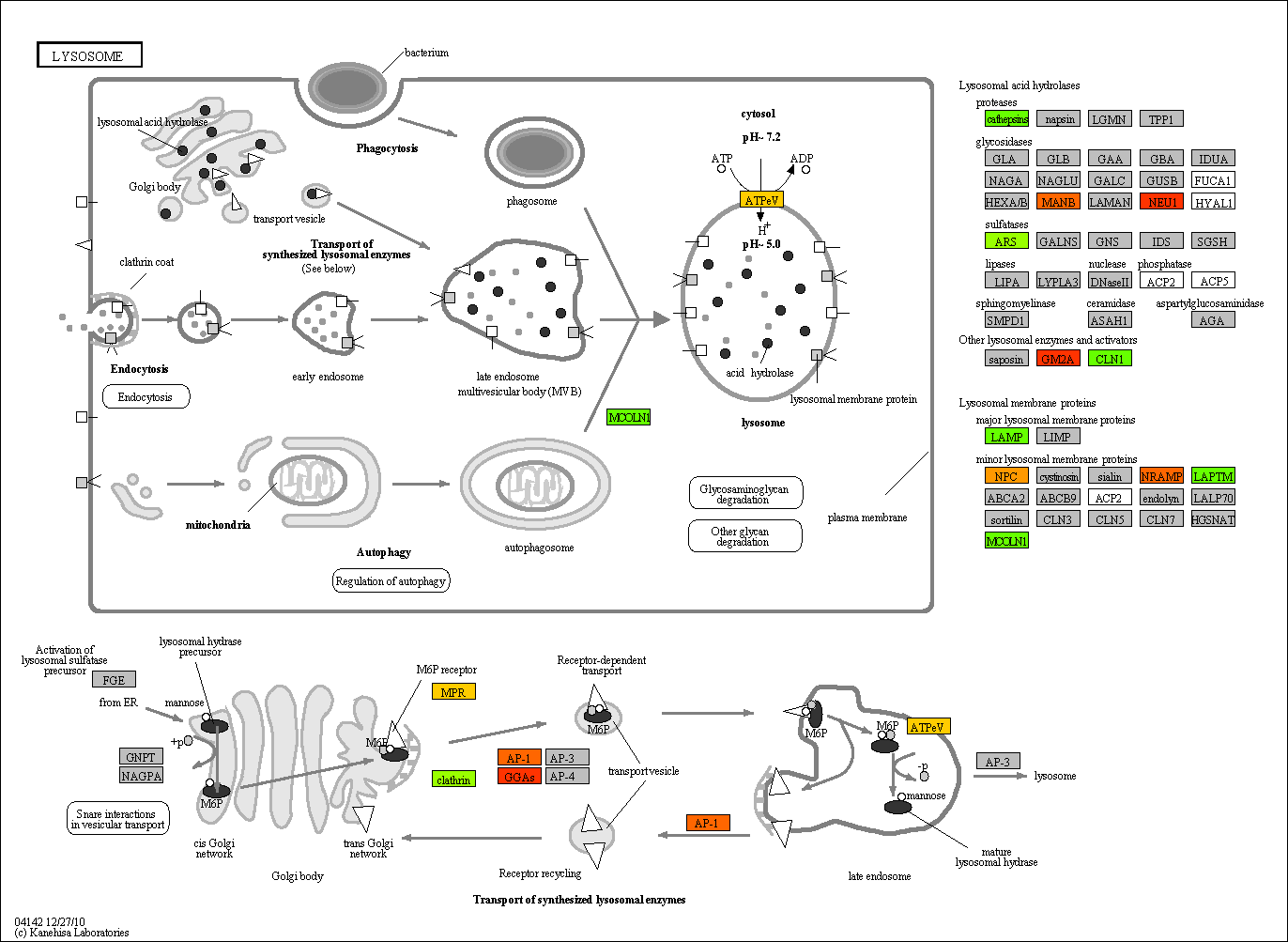


# 5. Organismal Systems

## 5.1 Immune system

### Complement and coagulation cascade

-14 DAYS

1 DAY 14 DAYS

### NOD-like receptor signaling pathway

-14 DAYS

1 DAY 14 DAYS

### Antigen processing and presentation

-14 DAYS

1 DAY 14 DAYS

## 5.2. Endocrine system

### Renin-angiotensin system

-14 DAYS

1 DAY 14 DAYS

### PPAR signaling pathway

-14 DAYS

1 DAY 14 DAYS
