# Supplementary material for: Integrative Analyses of Hepatic Differentially Expressed Genes and Blood Biomarkers during the Peripartal Period between Dairy Cows Overfed or Restricted-Fed Energy Prepartum
Source: PLoS One. 2014 Jun 10;9(6):e99757. doi: 10.1371/journal.pone.0099757 (PMC4051754; doi:10.1371/journal.pone.0099757)

**REVIGO analysis of GO biological process results from the Dynamic Impact Approach**

-14 DAYS

More expressed in OF vs. RE


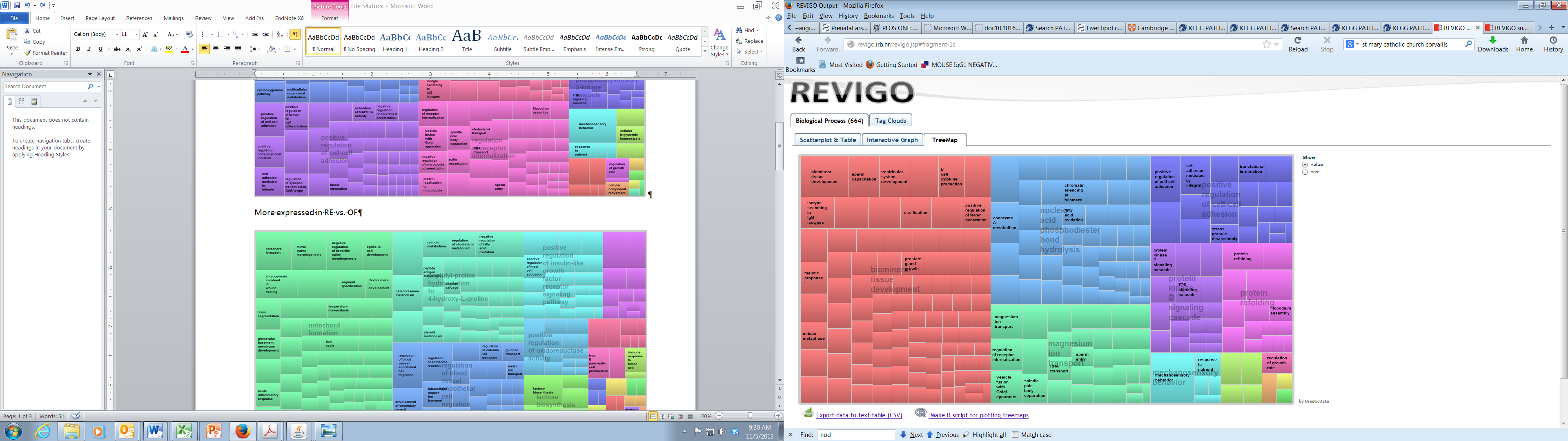


More expressed in RE vs. OF


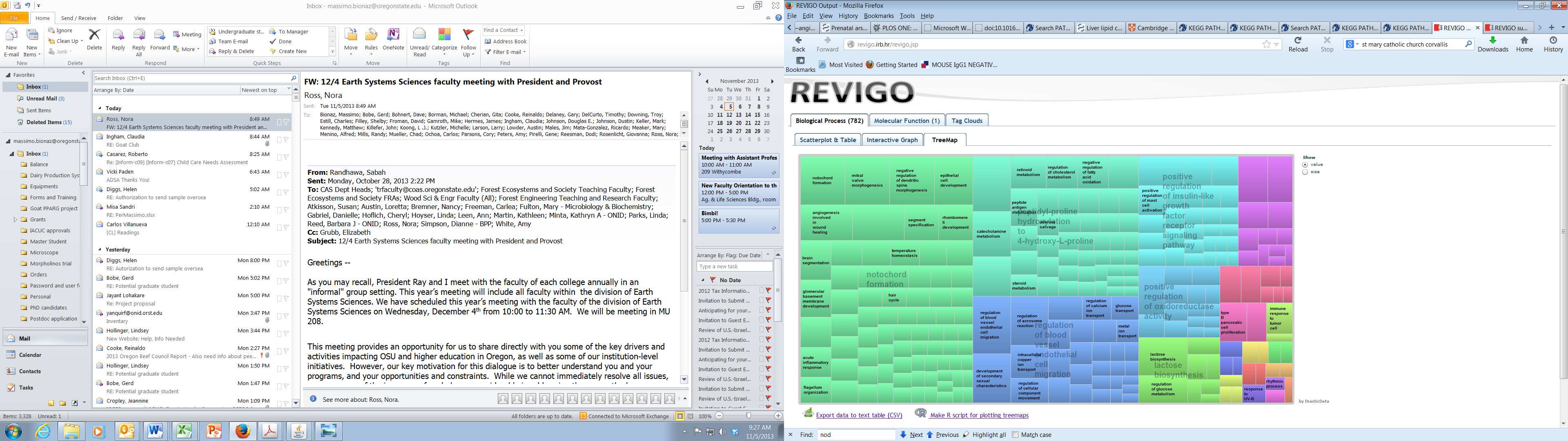


1 DAY

More expressed in OF vs. RE


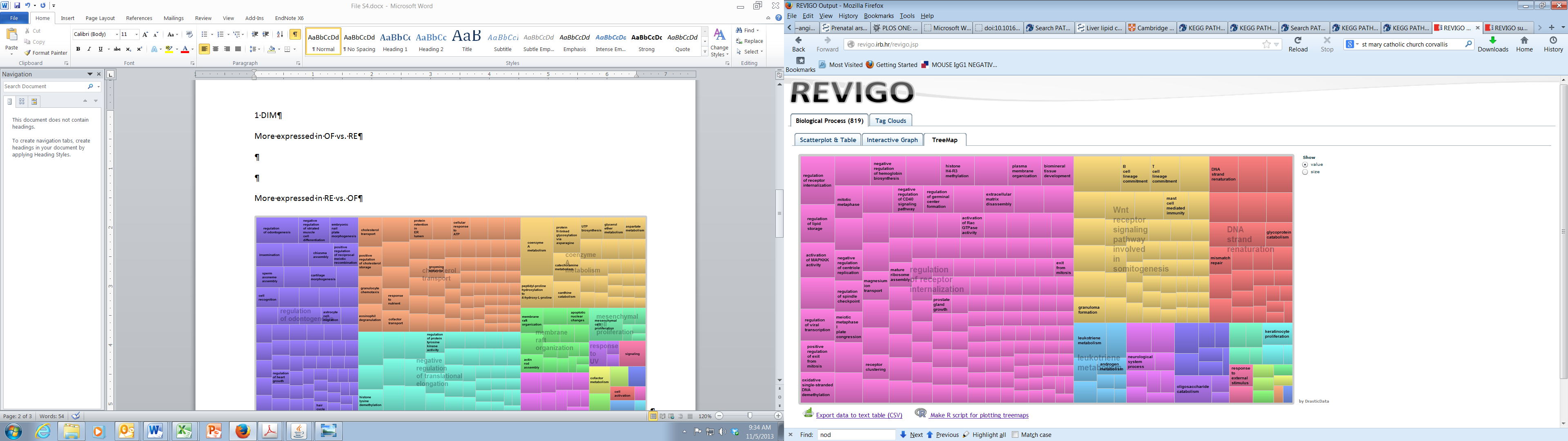


More expressed in RE vs. OF


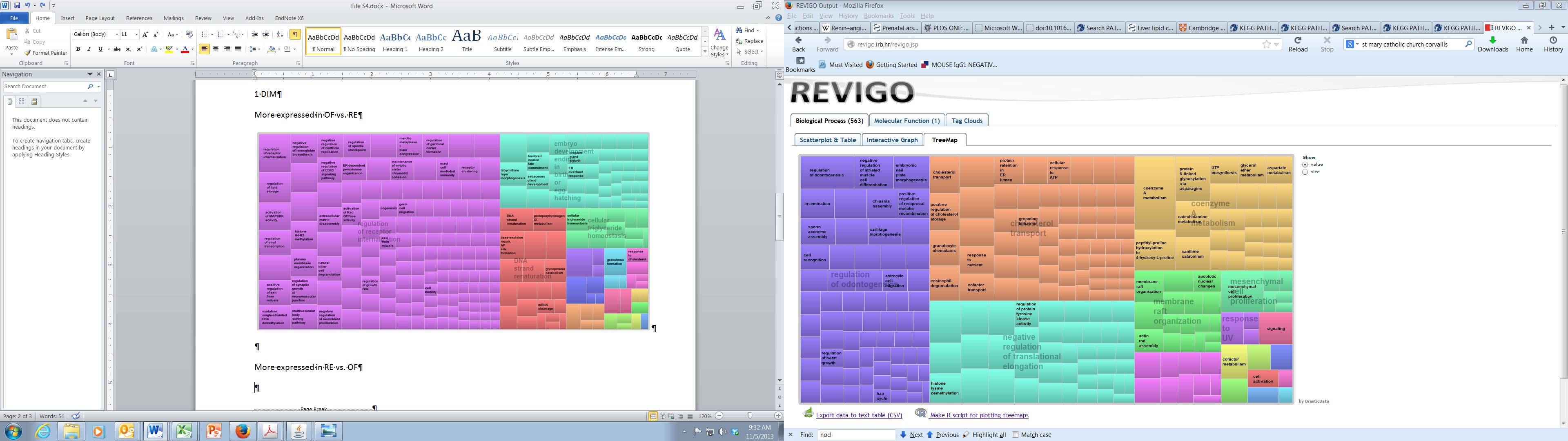


14 DAYS

More expressed in OF vs. RE


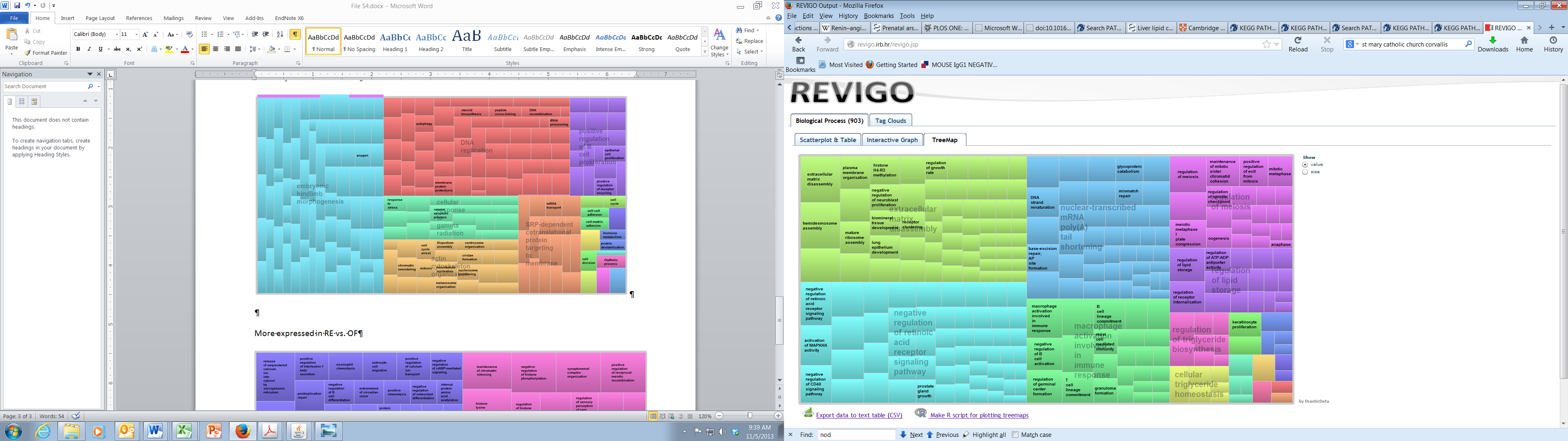


More expressed in RE vs. OF


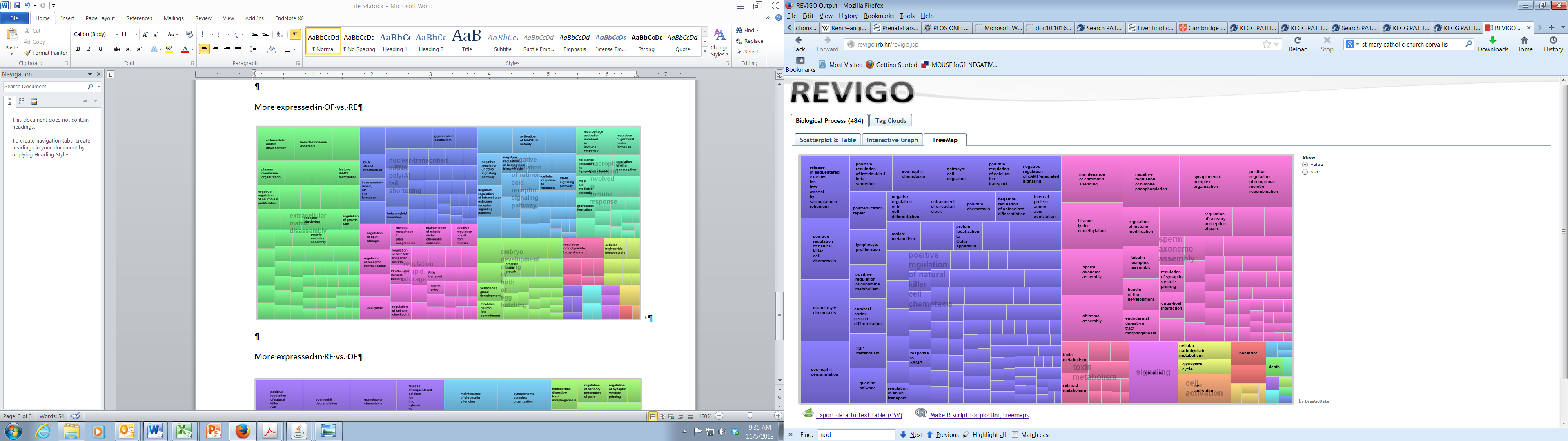

Supplement: File S4 — REVIGO ( http://revigo.irb.hr/ ) summary of the Dynamic Impact Apporach (DIA) analysis of the Gene Ontology biological processes (GO BP) affected in liver by prepartum dietary energy. The results are shown as Treemaps separated between terms more activated in OF vs. RE and the ones more activated in RE vs. OF. The dimension of each term is directly proportional to the overal induction. Same color indicate semantic and functional association. (DOCX) [file pone.0099757.s008.docx]
